# Enantioselective Route to Terminal 2,3‐Epoxy Tosylates Enabled by Cooperative Organocatalytic Desymmetrization of 2‐Substituted Glycerol Derivatives

**DOI:** 10.1002/chem.202502523

**Published:** 2025-10-23

**Authors:** Armando Astone, Sara Meninno, Alessandra Lattanzi

**Affiliations:** ^1^ Dipartimento di Chimica e Biologia “A. Zambelli” Università di Salerno Via Giovanni Paolo II Fisciano 84081 Italy

**Keywords:** cooperative catalysis, desymmetrization, epoxides, heterocyclic compounds, organocatalysis

## Abstract

A first enantioselective synthesis of challenging 2,2‐disubstituted 2,3‐epoxy tosylates has been developed via an organocatalytic desymmetrization reaction of bis tosylated 2‐substituted glycerols. The intramolecular nucleophilic substitution has been cooperatively catalyzed by commercially available mixtures of L‐diphenyl prolinol and (*R*,*R*)‐α,α,α’,α’‐tetraphenyl‐2,2 disubstituted 1,3‐dioxolane‐4,5‐dimethanol (TADDOL) or the enantiomeric couple, leading to (*R*)‐ and (*S*)‐epoxides in up to 87% yield and 76% ee. Reaction monitoring highlighted the double role of leaving group and nucleophile played by the *p*‐toluensulfonate anion, which was found to be responsible of epoxide ring‐opening back to the reagent. The latter process affects the conversion to the epoxide, but marginally the enantioselectivity. These uncommon epoxides, bearing a quaternary stereocenter, demonstrated to be versatile intermediates to prepare functionalized products and remarkably, privileged eight‐ and four‐ membered *N*‐heterocycles.

## Graphical abstract

A desymmetrization strategy from bis tosylated 2‐substituted glycerols to challenging 2,2‐disubstituted 2,3‐epoxy tosylates has been developed using commercial L‐diphenyl prolinol/(*R*,*R*)‐TADDOL system or the enantiomeric catalytic couple. (*R*)‐ or (*S*)‐ epoxides are obtained in moderate to good yield and up to 76% ee. Useful elaborations to functionalized products and remarkably to privileged eight‐ and four‐membered aza‐heterocycles, were demonstrated.

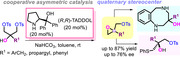

## 1 Introduction

Chiral non‐ racemic 2,3‐epoxy alcohols are a renowned class of oxiranes, broadly occurring key building‐blocks or intermediates in a great number of asymmetric syntheses of natural, nonnatural, and bioactive compounds.^[^ ^1^, ^2^ ^]^ The popular method for their preparation is the titanium‐tartrate/*tert*‐butyl hydroperoxide‐ catalyzed asymmetric epoxidation of allylic alcohols,^[^ ^3^ ^]^ a process characterized by a large substrate scope, predictable stereochemical outcome, and high level of enantioselectivity. Over the time, examples of chiral ligand vanadium‐,^[^ ^4^, ^5^, ^6^, ^7^ ^]^ niobium‐,^[^ ^8^, ^9^ ^]^ hafnium‐,^[^ ^10^, ^11^ ^]^ tungsten^[^ ^12^ ^]^‐catalyzed asymmetric epoxidation reactions of allylic alcohols widened the number of methodologies useful to access this class of functionalized oxiranes.

**Figure 1:**
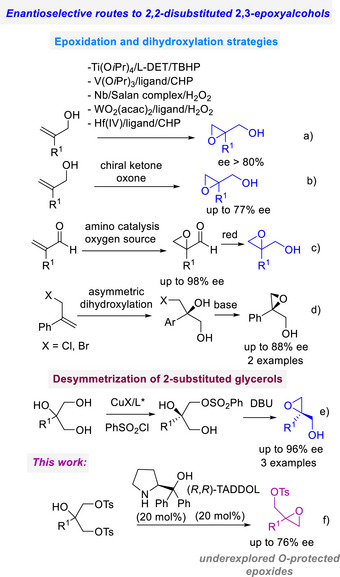
Asymmetric routes to 2,2‐disubstituted 2,3‐epoxy alcohol derivatives.

Among them, the 2,2‐disubstituted 2,3‐epoxy alcohols represent a challenging target, whose synthesis through metal‐catalyzed asymmetric epoxidation provides the products in good to high enantioselectivity (Figure 1a). Organocatalytic asymmetric oxidation by chiral dioxiranes proved to be an alternative process (Figure 1b).^[^ ^13^ ^]^ Additionally, 2,2‐disubstituted 2,3‐epoxy alcohols have been prepared through a two‐step sequence based on an amino catalyzed asymmetric epoxidation of 2‐substituted enals,^[^ ^14^ ^]^ followed by reduction of the epoxy aldehyde with NaBH_4_ (Figure 1c).^[^ ^15^ ^]^ Another oxidation strategy has been illustrated by the Sharpless‐ catalyzed asymmetric dihydroxylation of 2‐phenyl allylic chloride or bromide, followed by basic treatment to prepare representative terminal epoxy alcohols in up to 88% ee.^[^ ^16^ ^]^

A nonoxidative preparation has been developed, exploiting the desymmetrization of 2‐substituted‐1,2,3‐triol. In 2023, Gu, Hong and Liu illustrated an appealing S─O cross‐coupling reaction under Cu(I)/chiral ligand catalysis of 2‐substituted‐1,2,3‐triol and phenylsulfonyl chloride (Figure 1e).^[^ ^17^ ^]^ The monosulfonylated congested alcohols were isolated in satisfactory yield and high enantioselectivity. In showcasing the synthetic potential of these products, a base‐assisted intramolecular displacement was exemplified to obtain the corresponding terminal epoxy alcohols.

Thanks to the highly regioselective ring‐opening reactions they can undergo, terminal epoxides represent the most versatile oxiranes, of high value for synthetic applications.^[^ ^18^, ^19^, ^20^, ^21^, ^22^ ^]^

We have been interested in the development of asymmetric epoxidation reactions of functionalized alkenes, using optically active alkyl hydroperoxides^[^ ^23^ ^]^ or organocatalytic systems.^[^ ^24^, ^25^, ^26^ ^]^ The development of asymmetric processes, beyond typical asymmetric oxidation of alkenes, prompted us to investigate a conceptually different desymmetrization approach. Starting from bis‐*O*‐protected 2‐substituted glycerols, we envisioned that a bifunctional organocatalyst might selectively catalyze an intramolecular nucleophilic substitution to yield enantioenriched *O*‐protected terminal 2,3‐epoxy alcohols (Figure 1f).

The latter are uncommon dielectrophilic intermediates, susceptible of elaboration either via epoxide ring‐opening or nucleophilic displacement of the leaving group, whose reactivity has been up to now underexplored. Herein, we illustrate the synthesis of 2,2‐disubstituted 2,3‐epoxy tosylates via desymmetrization of bis‐tosylated 2‐substituted glycerols catalyzed by the commercially available L‐diphenyl prolinol and (*R*,*R*)‐TADDOL or the enantiomeric systems, in toluene at room temperature. The products, recovered in moderate to good yields and up to 76% ee, proved to be valuable intermediates to access ring‐opened derivatives and privileged *N*‐based heterocycles.

## 2 Results and Discussion

**Scheme 1:**
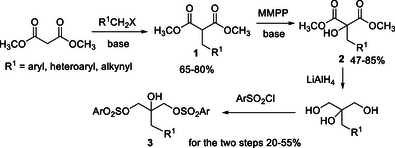
Four‐step sequence for the synthesis of bis arylsulfonyl 2‐substituted glycerols **3**.

We commenced the investigation with the synthesis of the bis tosylates **3**, through a four‐step sequence involving malonate alkylation/magnesium monoperoxyphthalate (MMPP) mediated α‐hydroxylation^[^ ^27^ ^]^/reduction/sulfonylation (Scheme 1).

A variety of bis‐sulfonylated 2‐substituted glycerols **3** has been easily prepared as the starting material for the desymmetrization process. Model reaction of compound **3a** (R^1^ = Ph, Ar = *p*‐Tol) was then carried out in the presence of common bifunctional organocatalysts, NaHCO_3_ as additive, at room temperature in toluene (Table 1). Blank experiments were first carried out in the presence of inorganic bases to check the involvement of parallel racemic processes.

**Table 1:** Catalyst optimization in the desymmetrization of compound **3a**.^[a]^

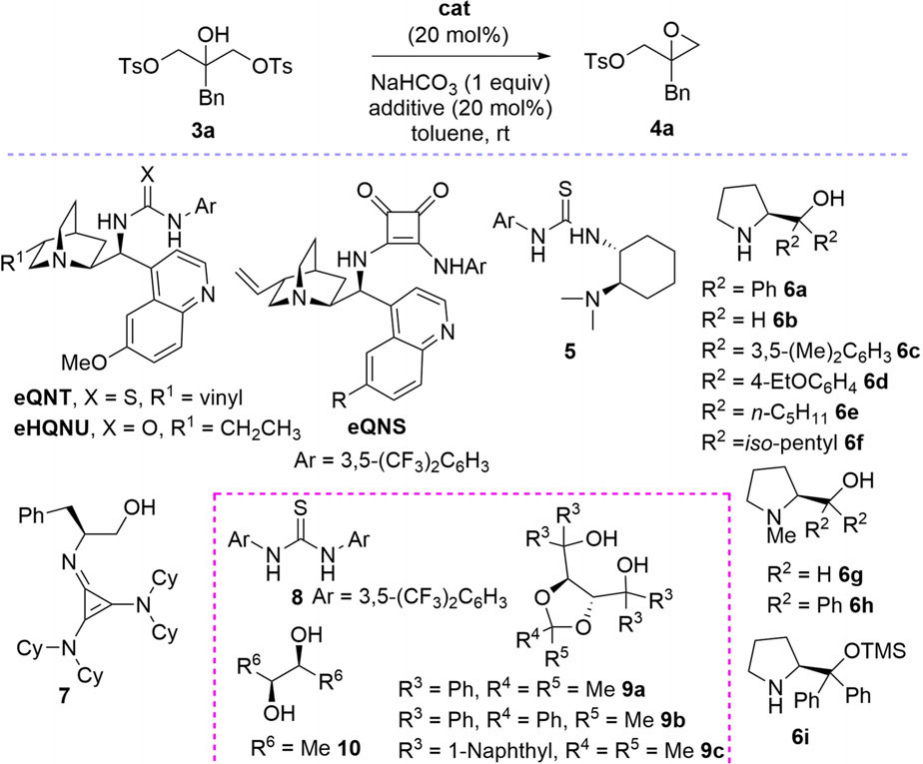					
Entry	cat	Additive	t [h]	Conv. [%]^[b]^	ee [%]^[c]^
1^[d]^	‐	‐	64	<5	‐
2^[e]^	‐	‐	88	65	‐
3	**QN**	‐	77	45	15
4	***e*QNT**	‐	72	20	58
5	***e*HQNU**	‐	70	28	43
6	***e*QNS**	‐	65	<5	‐
7	**5**	‐	240	9	−21
8	**6a**	‐	64	26	−58
9	**6b**	‐	64	33	−16
10	**6c**	‐	168	10	−27
11	**6d**	‐	144	19	−40
12	**6e**	‐	144	14	−31
13	**6f**	‐	144	25	−40
14	**6g**	‐	64	27	7
15	**6h**	‐	64	<5	‐
16	**6i**	‐	72	<5	‐
17	**7**	‐	144	16	−10
18	**6a**	**8**	120	40	−30
19	**6a**	**9a**	144	36	−76
20	**6a**	*ent*‐**9a**	96	14	−49
21	**6a**	**9b**	96	24	−69
22	**6a**	**9c**	150	19	−60
23	*ent*‐**6a**	**9a**	144	34	61
24	*ent*‐**6a**	*ent*‐**9a**	96	21	65
25	**6a**	**10**	96	60	−20
26	**6a**	*ent*‐**10**	96	55	−24
27^[f]^	**6a**	**9a**	144	31	−71
28^[g]^	**6a**	**9a**	144	38	−66

^[a]^ Reaction conditions: **3a** (0.1 mmol), **cat** (0.02 mmol), NaHCO_3_ (0.1 mmol) in anhydrous toluene (0.5 mL).

^[b]^ Yield determined by ^1^H NMR analysis of the crude reaction mixture.

^[c]^ HPLC analysis on a chiral stationary phase. The minus sign indicates the recovery of the opposite enantiomer.

^[d]^ NaHCO_3_ (1 equiv) was used.

^[e]^ K_2_CO_3_ (1 equiv) was used.

^[f]^ 30 mol% of **6a** and **9a** were used.

^[g]^ 20 mol% of **6a** and 40 mol% of **9a** were used.

NaHCO_3_ proved to be essentially inert for any catalysis as the reagent was recovered unaffected after prolonged reaction time (entry 1). On the contrary, when using stronger K_2_CO_3_, the expected product **4a** was markedly formed (entry 2). Consequently, NaHCO_3_ was chosen as acid scavenger to restore the bifunctional catalysts over the catalytic cycle. When using quinine a satisfactory conversion to **4a** was observed, but with poor enantiocontrol (entry 3). Interestingly, with the quinine‐ derived thiourea ***e*QNT**, the reaction proceeded less efficiently, but the ee raised to 58% (entry 4). The dihydroquinine urea ***e*HQNU**, the quinine‐ derived squaramide ***e*QNS** and Takemoto's catalyst **5** proved to be scarcely effective (entries 5, 6, and 7). Given the better activity showed by quinine (entry 3), we thought to use other amino alcohols as catalysts. L‐diphenyl prolinol **6a** proved to be promising, being the product obtained with 58% ee (entry 8). Less sterically demanding **6b**, more sterically demanding **6c** and *para*‐phenyl substituted **6d** provided inferior results (entries 9, 10, and 11). Unfortunately, aliphatic substituted prolinols **6e** and **6f** did not improve the process (entries 12 and 13). *N*‐methyl prolinols **6g** and **6h** led to disappointing results, indicating that catalysts, bearing the secondary amine, are more efficient (entries 14 and 15). Finally, the Hayashi–Jørgensen catalyst **6i** did not promote the desymmetrization, which clearly indicates the importance of the free OH group in the catalysis (entry 16). With a view to increase the conversion, superbase amino alcohol **7**,^[^ ^28^ ^]^ the Lambert's catalyst was used, achieving a surprisingly poor result (entry 17).

**Figure 2:**
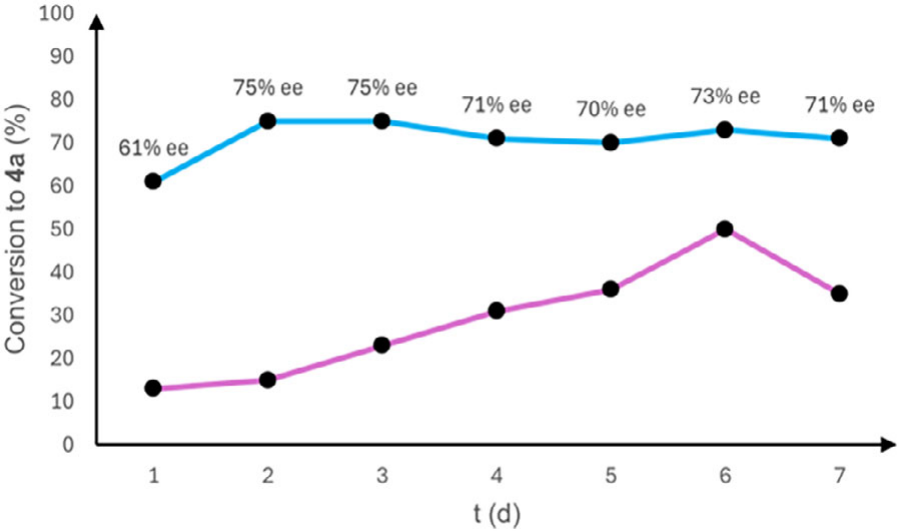
Reaction progress profile by ^1^H‐NMR of compound **3a** under conditions reported in entry 19 of Table 1, pink line (conversion to **4a**), light blue line (ee of **4a**).

To further improve the process, H‐bonding donor additives were added using catalyst **6a**. Unfortunately, the Schreiner's thiourea was not beneficial (entry 18).^[^ ^29^ ^]^ Pleasingly, the addition of (*R*,*R*)‐TADDOL **9a** gave **4a** in an acceptable conversion and 76% ee, after prolonged reaction time (entry 19). Decreased enantioselectivity (entry 20) was observed when using *ent*‐**9a**, showing a mismatching effect. The addition of more sterically demanding TADDOL **9b** was beneficial, but less effectively than compound **9a** (entry 21). Increasing further the steric features with **9c** turned out to be detrimental (entry 22). To confirm the best performing cooperative couple of catalyst/TADDOL, reactions with *ent*‐**6a/9a** and *ent* **‐6a** */ent*‐**9a** were then carried out (entries 23 and 24). Product **4a** was obtained in the opposite enantiomerically enriched form as expected for matching effects (entry 24). Simple diol additives **10** and *ent*‐**10** afforded good conversions, but at the expense of enantioselectivity (entries 25 and 26). Unexpectedly, reactions carried out increasing the loading of **6a** and **9a** to 30 mol % or doubling to 40 mol % the amount of **9a** did not improve the conversion (entries 27 and 28). These results prompted us to study the reaction outcome of compound **3a** over time, under the most effective conditions reported in entry 19 of Table 1 (Figure 2).

The conversion to product **4a** slowly increased over 6 days up to around 50% with the ee values ranging around 70%–75%. However, after 7 days, a decrement of the conversion was observed, which would suggest the occurrence of a reversible ring‐opening reaction of epoxide **4a** to reagent **3a** by the *p*‐toluensulfonate anion accumulated in the reaction vessel.

**Scheme 2:**
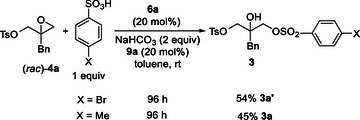
Ring‐opening reactions of epoxide **4a** by benzensulfonates under optimized conditions.

In order to confirm this hypothesis, two experiments were performed under conditions reported in entry 19 of Table 1 (Scheme 2).

4‐Bromobenzensulfonic acid was added to the reaction mixture containing racemic epoxide **4a**, in the presence of the catalytic couple **6a**/**9a** and a slight excess of NaHCO_3_ (2 equiv) to generate the sulfonate anion. After 96 hours, the mixed reagent **3a’**, obtained via epoxide ring‐opening, was isolated in 54% yield. When using *p*‐toluensulfonic acid as the reagent, compound **3a** was obtained in 45% yield. Unreacted epoxide **4a**, checked by chiral HPLC analysis, proved to be racemic, thus attesting that a process of kinetic resolution is not involved in the reverse reaction.

Solvent screening and the presence of other sulfonyl groups on model 2‐benzyl glycerol were studied (see the Supporting Information), confirming the bis‐tosylated 2‐substituted glycerols and toluene as the most effective combination.

**Scheme 3:**
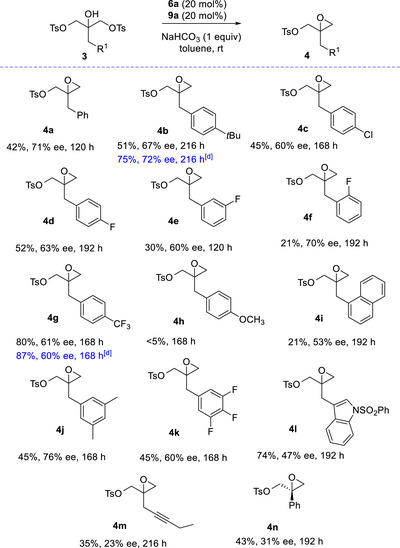
Scope of the desymmetrization to 2,2‐disubstituted 2,3‐epoxy tosylates^[a–c]^.^[a]^Reaction conditions: **3** (0.1 mmol), **6a** (0.02 mmol), **9a** (0.02 mmol), NaHCO_3_ (0.1 mmol) in anhydrous toluene (0.5 mL). ^[b]^Yield of isolated product after chromatography. ^[c]^HPLC analysis on a chiral stationary phase. ^[d]^20 mol% of *ent*‐**6a** and 20 mol% of *ent*‐**9a** was used, providing the opposite enantiomer of the product.

We next investigate the scope and limitations of the bis‐tosylated 2‐substituted glycerols **3** desymmetrization reaction, working under the optimized conditions (Scheme 3).

Bis‐Tosylated 2‐substituted glycerols **3**, bearing alkyl group and halogen atoms at the *para*‐position of the phenyl ring, afforded compounds **4b–d** with similar yield and enantioselectivity of model epoxide **4a**. Compounds **3e**,**f**, fluorine‐substituted at *meta* and especially at the *ortho*‐positions, were converted to the epoxide more slowly, although comparable level of enantioselectivity was observed. The *para*‐CF_3_ phenyl‐substituted derivative **3g** provided the epoxide **4g** in 80% yield and 61% ee, whereas the *para*‐OCH_3_ phenyl‐substituted derivative **3h** did not react. These data suggest that the nature of the substituent in the phenyl ring would affect the acidity of the free OH group, a key factor in the aminoalcohol‐catalyzed intramolecular substitution.

The slow conversion to the sterically more demanding compound **4i** was likely due to the lower solubility of reagent **3i** observed under the reaction conditions. Polysubstituted in the phenyl ring and the indolyl compounds **3j**,**k**,**l** furnished the epoxides in satisfactory yield and enantioselectivity similarly to that observed for previous epoxides. Commercial availability of the organocatalysts, enables a not obvious preparation of the epoxides with the opposite absolute configuration, exemplified with products **4b** and **4g** satisfactorily prepared, using the *ent*‐**6a**/*ent*‐**9a** system. Finally, bis‐*p*‐tolylsulfonylated 2‐substituted glycerols, bearing propargylic or phenyl moieties **3m**,**n**, proved to be less suitable substrates for the desymmetrization, being the products **4m**,**n** isolated in still acceptable yields, but significantly lower enantioselectivity. The prevalent formation of (*R*)‐**4n** was assessed by comparing the optical rotation with the literature.^[^ ^30^ ^]^

**Scheme 4:**
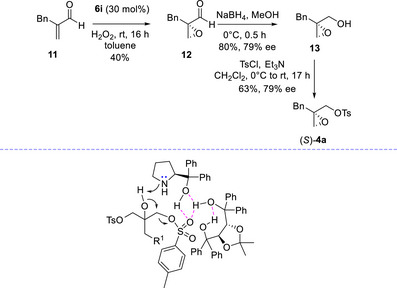
Determination of the absolute configuration of compound **4a** and hypothetical activation mode of reagent **3**.

The absolute configuration of the other 2,3‐epoxy tosylates was established by chemical correlation, taking advantage of the asymmetric epoxidation of acroleins organocatalyzed by **6i** with hydrogen peroxide,^[^ ^15^, ^31^ ^]^ followed by reduction and tosylation (Scheme 4).

The epoxidation afforded aldehyde **12** with 40% yield, which was then reduced to the epoxyalcohol **13** in 80% yield and 79% ee.^[^ ^32^ ^]^ Finally, tosylation of compound **13** afforded (*S*)‐**4a** in 63% yield and 79% ee. Comparison of the optical rotation with the literature enabled to assign the *R* absolute configuration to compound **4a**, reported in Scheme 3. The absolute configuration of compounds **4b–** **g** and **4j,k** was assigned by analogy.

^1^H‐NMR analysis of catalytic mixture of **6a** and **9a** in C_6_D_6_ showed plausible H‐bonding interactions between the OH proton of TADDOL with the prolinol. When adding **3a** to catalysts **6a** and **9a** small shifts at lower field, of the aromatic protons of the *p*‐tolyl group, were observed (see the Supporting Information). Based on this, the activation of compound **3** is supposed to occur through assistance in the OH deprotonation by the diaryl prolinol, whereas a cooperative H‐bonding donors network, comprising the diarylcabinol unit and the TADDOL hydroxyls, might assist a selective *p*‐toluensulfonate anion departure in the chiral environment (Scheme 4). This is a first example of cooperative noncovalent activation of the reagents, provided by a chiral amino alcohol and a chiral H‐bonding donor. In this context, cooperative noncovalent organocatalytic asymmetric reactions by means of chiral thioureas or chiral phosphoric acid and an achiral H‐bonding donor, have been previously reported.^[^ ^33^, ^34^, ^35^, ^36^ ^]^

**Scheme 5:**
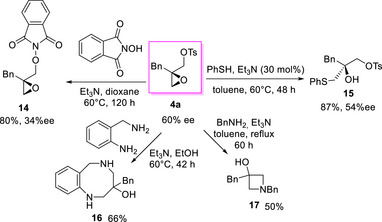
Synthetic elaborations of enantioenriched **4a** with *O*‐, *S*‐, and *N*‐based nucleophiles.

The scale up of model reaction at 1 mmol scale afforded enantioenriched compound (*R*)‐**4a**, which was subjected to nucleophilic displacements to investigate the underexplored synthetic potential of these dielectrophilic intermediates (Scheme 5).^[^ ^37^, ^38^, ^39^ ^]^

Compound **4a** treated with *N*‐hydroyl phtalimide under basic conditions at 60°C, underwent tosyl group substitution to give epoxide **14** in 80% yield and 34% ee. The significant racemization observed could be rationalized considering that the reaction is not selective, but the following processes occurred: i) prevalent displacement of the tosyl group by the *N*‐hydroyl phtalimide; ii) partial epoxide ring‐opening by the *N*‐hydroyl phtalimide, followed by intramolecular terminal epoxide formation, leading to product **14** with opposite absolute configuration.

On the contrary, the addition of thiophenol selectively proceeded with epoxide ring opening to afford functionalized alcohol **15** in 87% yield and 54% ee. With a view to access heterocyclic compounds, epoxide **4a** was then reacted with 2‐aminobenzyl amine under reflux.

Remarkably, a double nucleophilic substitution occurred, leading to an unprecedented eight‐membered heterocycle **16** in 66% yield, but unfortunately, it was not possible to determine the enantiomeric excess.^[^ ^40^ ^]^ The formation of the hexahydrobenzodiazocine **16** is noteworthy, being the cyclization process disfavored by entropic factor/transannular interactions^[^ ^41^ ^]^ and given the absence of synthetic protocols to obtain compounds **16** and derivatives thereof.

Interestingly, when compound **4a** was treated with benzylamine, a double nucleophilic substitution led to the four‐membered azetidinol **17** in 50% yield. Large and small *N*‐heterocycles **16** and **17** are recurrent motifs found in approved drugs and the access through epoxides **4** favorably adds to the limited tools available for their synthesis.^[^ ^42^ ^]^

## 3 Conclusion

In conclusion, a first enantioselective route to challenging 2,2‐disubstituted 2,3‐epoxy tosylates has been developed through a desymmetrization strategy of bis‐tosylated 2‐substituted glycerols. Commercially available diphenyl prolinol/TADDOL catalytic systems promoted an intramolecular displacement to the epoxides in both absolute configurations, obtained in moderate to satisfactory yield and up to 76% ee. The synthetic versatility of the epoxides, bearing a quaternary stereocenter, has been demonstrated through their transformations to functionalized products with oxygen and sulfur‐ based nucleophiles. An appealing preparation of valuable eight‐ and four‐membered nitrogen‐ based heterocycles has been showcased when using bis‐ and mono‐amines. Additional studies will be necessary to improve this desymmetrization strategy in terms of substrate scope and organocatalysts suitable to match an enhancement of the efficiency.

This study brought to light that the diphenyl prolinol/TADDOL couple serves as an unprecedented and readily available system to provide cooperative noncovalent organocatalysis. TADDOL appeared to enhance the chiral environment of bifunctional diphenyl prolinol, through H‐bonding interactions.^[^ ^43^ ^]^ Given the significant number of easily accessible chiral diols and bifunctional organocatalysts is likely to foresee that this or related systems will be useful to promote other catalytic reactions.

## 4 Experimental Section

### General procedure for the desymmetrization to 2,2‐Disubstituted 2,3‐Epoxy Tosylates

In a round‐bottom flask, sodium bicarbonate (8.4 mg, 0.1 mmol), **6a** (5.1 mg, 0.02 mmol) and **9a** (9.3 mg, 0.02 mmol) were added to a solution of the proper bis‐tosylated 2‐substituted glycerol **3** (0.1 mmol) in toluene (0.5 mL). The reaction was stirred at room temperature for 5–9 days (monitored by TLC, using *n*‐hexane/ethyl acetate 8/2 as eluent, visualized by UV light and phosphomolybdic acid solution). After completion, the crude mixture was purified by flash chromatography (eluent: *n*‐hexane/diethyl ether, gradient from 100/0 to 90/10) to isolate the enantioenriched epoxides **4a–n**. The synthesis of (*S*)‐**4b** and (*S*)‐**4g** was carried out by using *ent*‐**6a**/*ent*‐**9a** catalysts.

## Supporting Information

Experimental procedures and characterization data for all new compounds. The authors have cited additional references within the Supporting Information.^[^^44–61^^]^

## Conflict of Interest

The authors declare no conflict of interest.

## Supporting information



Supporting Information

## Data Availability

The data that support the findings of this study are available in the supplementary material of this article.
