# Supplementary material for: Enantioselective Route to Terminal 2,3‐Epoxy Tosylates Enabled by Cooperative Organocatalytic Desymmetrization of 2‐Substituted Glycerol Derivatives
Source: Chemistry. 2025 Oct 23;31(66):e02523. doi: 10.1002/chem.202502523 (PMC12648462; doi:10.1002/chem.202502523)
Supplement: Supplementary file 1 — Supporting Information [file CHEM-31-e02523-s001.docx]

**Supporting Information**

**Enantioselective Route to Terminal 2,3-Epoxy Tosylates Enabled by Cooperative Organocatalytic Desymmetrization of 2-Substituted Glycerol Derivatives**

Armando Astone,^a^ Sara Meninno^a^ and Alessandra Lattanzi*^a^

*^a^Dipartimento di Chimica e Biologia “A. Zambelli”, Università di Salerno, Via Giovanni Paolo II, 84081, Fisciano, Italy*

[General Methods S2](#_Toc205623650)

[Experimental Procedures and Compounds Characterization S3](#_Toc205623651)

[**General procedures for the synthesis of catalyst 6d** S3](#_Toc205623652)

[**General procedures for the synthesis of catalysts 6e and 6f** S4](#_Toc205623653)

[**Procedure for the bromination of (1-(phenylsulfonyl)-1*H*-indol-3-yl)methanol** S6](#_Toc205623654)

[**3-(bromomethyl)-1-(phenylsulfonyl)-1*H*-indole** S6](#_Toc205623655)

[**Four-step sequence for the synthesis of bis arylsolfonyl 2-substituted glycerols 3** S7](#_Toc205623656)

[**Alkylation of dimethyl malonate 1** S7](#_Toc205623657)

[**Oxidation of variously substituted dimethyl 2-benzylmalonates 2** S11](#_Toc205623658)

[**Reduction of substituted dimethyl 2-benzyl-2-hydroxymalonates** S16](#_Toc205623659)

[**General procedure for the protection of 2-substituted glycerols for the synthesis of starting materials 3** S16](#_Toc205623660)

[**Synthesis of 2-hydroxy-2-phenylpropane-1,3-diyl bis (4-methylbenzenesulfonate) 3n** S23](#_Toc205623661)

[**General procedure for the synthesis of racemic epoxides 4** S24](#_Toc205623662)

[**Table S1.** Screening of the protecting group S25](#_Toc205623663)

[**Table S2.** Solvent screening S25](#_Toc205623664)

[**Graphic S1.** Study of desymmetrization process over seven days S26](#_Toc205623665)

[**General procedure for the synthesis of enantioenriched epoxides 4o, 4p, 4q** S26](#_Toc205623666)

[**General procedure for the synthesis of enantioenriched epoxides 4a-n** S27](#_Toc205623667)

[**Determination of the absolute configuration of epoxide 4a** S28](#_Toc205623668)

[**Scale-up of the asymmetric synthesis of epoxide (*R*)-4a** S36](#_Toc205623669)

[Ring opening reaction of racemic epoxide 4a by 4-bromobenzenesulfonate S36](#_Toc205623670)

[Elaboration of the enantioenriched epoxide 4a S37](#_Toc205623671)

[**Procedure for the synthesis of (*R*)-2-((2-benzyloxiran-2-yl)methoxy)isoindole-1,3-dione 14** S37](#_Toc205623672)

[**Procedure for the synthesis of (*S*)-2-benzyl-2-hydroxy-3-(phenylthiol)propyl 4-methylbenzenesulfonate 15** S38](#_Toc205623673)

[**Procedure for the synthesis of 3-benzyl-1,2,3,4,5,6-hexahydrobenzo[*b*][1,5]diazocin-3-ol 16** S39](#_Toc205623674)

[**Procedure for the synthesis of 1,3-dibenzylazetidin-3-ol 17** S39](#_Toc205623675)

[Derivatization of 3-benzyl-1,2,3,4,5,6-hexahydrobenzo[*b*][1,5]diazocine-3-ol 16 S40](#_Toc205623676)

[^1^H NMR shifts for catalytic system activation S41](#_Toc205623677)

[NMR Spectra S42](#_Toc205623678)

[HPLC Chromatograms S179](#_Toc205623679)

# General Methods

All reactions requiring anhydrous or inert conditions were conducted in flame-dried glassware under a positive nitrogen pressure. Anhydrous THF and pyridine were purchased from Merck-Sigma-Aldrich, and used as received; all other solvents were dried over molecular sieves. Molecular sieves (Aldrich Molecular Sieves, 3Å, 1.6 mm pellets) were activated under vacuum at 200 °C overnight. Reactions were monitored by thin layer chromatography (TLC) on Merck pre-coated silica gel plates (0.25 mm) and visualized by UV light, and where indicated, by phosphomolybdic acid, or ninhydrin solutions. Flash chromatography was performed on Merck silica gel (60, particle size: 0.040–0.063 mm). ^1^H NMR, ^13^C NMR and ^19^F NMR spectra were recorded on Bruker Avance III HD 600, Bruker Avance-400 or Bruker Avance-300 spectrometer in CDCl_3_. Chemical shifts for protons are reported using residual solvent protons (*δ* = 7.26 ppm for CDCl_3_) as internal standard. Carbon spectra were referenced to the shift of the ^13^C signal of CDCl_3_ (*δ* = 77.0 ppm).

The following abbreviations are used to indicate the multiplicity in NMR spectra: s - singlet; d - doublet; t - triplet; dd – double doublet; ddd – doublet of doublet of doublet; dt – double triplet; m - multiplet; bs - broad signal.

Optical rotation of compounds was performed on a Jasco P-2000 digital polarimeter using WI (Tungsten-Halogen) lamp (λ = 589 nm). High resolution mass spectra (HRMS) were acquired using a Bruker solariX XR Fourier transform ion cyclotron resonance mass spectrometer (Bruker Daltonik GmbH, Bremen, Germany) equipped with a 7 T refrigerated actively-shielded superconducting magnet. The samples were ionized in positive ion mode using a MALDI or ESI ionization sources. Melting points were measured with a Stuart Model SMP 30 melting point apparatus and are uncorrected. All starting materials (unless otherwise noted) were purchased from Merck-Sigma Aldrich, or TCI-Europe and used as received. Enantiomeric excess of epoxide **4** were determined by HPLC (Waters-Breeze 2487, UV dual λ absorbance detector and 1525 Binary HPLC Pump) using Daicel chiral columns. The absolute configuration of **4a** was assigned as *R* by comparison with the literature.^58-59^

#

# Experimental Procedures and Compounds Characterization

## **General procedures for the synthesis of catalyst 6d**

Catalyst **6d** was prepared according to the literature.^[[1]](#footnote-1),^ ^[[2]](#footnote-2)^

In a round-bottom flask, a solution of **S1** (2.86 g, 17.3 mmol) and DMAP (2.11 g, 17.3 mmol) in CH_2_Cl_2_ (52 mL) was added. After, a solution of (Boc)_2_O (4.15 g, 19.0 mmol) in CH_2_Cl_2_ (8.60 mL) was added dropwise at 0 °C. The reaction mixture was stirred at room temperature for 24 hours. After completion, the solution was washed with deionised water (3 x 10 mL) and brine (3 x 10 mL). The organic phase was dried over Na_2_SO_4_ and concentrated under vacuum. The product **S2** was used in the following step without further purification. To a solution of the corresponding magnesium bromide (1M, 13.1 mmol, 13.1 mL), a solution of **S2** (4.36 mmol) in anhydrous THF (7.92 mL) was added dropwise in 30 minutes. The reaction mixture was refluxed for 14 hours, monitored by TLC (eluent *n*-hexane/EtOAc 7/3, revealed by UV light and phosphomolybdic solution). After completion, the solution was quenched with ammonium chloride solution and extracted with ethyl acetate (3 x 10 mL). The combined organic phases were dried over Na_2_SO_4_ and concentrated under vacuum. The crude reaction mixture was purified by flash chromatography (*n*-hexane/EtOAc, 100:0 to 70:30) to obtain the product **S3** (64% yield).

In a round-bottom flask under nitrogen, **S3** (0.56 mmol) and NaOH (223.9 mg, 5.6 mmol) in EtOH (2.8 mL) were added. The reaction mixture was refluxed for 3 hours. After completion, the solvent was evaporated, and the crude was dissolved in water (1.2 mL) and diethyl ether (1.2 mL). The layers were separated, and the aqueous phase was washed with diethyl ether (2 x 10 mL). The combined organic phases were washed with water (10 mL) and brine (10 mL), dried over Na_2_SO_4_ and concentrated under vacuum. The crude reaction mixture was purified by flash chromatography (CHCl_3_/MeOH, 100:0 to 90:10) to obtain the product **6d** (17% yield).

**(*S*)-bis(4-ethoxyphenyl)(pyrrolidine-2-yl)methanol (6d)**

Colourless oil (flash chromatography, eluent CHCl_3_/MeOH from 100/0 to 90/10), 32 mg, 17% yield. [α]^24^ = - 53.1° (C = 0.86, CHCl_3_). **^1^H NMR** (300 MHz, CDCl_3_): *δ* 7.44 (d, 2H, J = 8.84 Hz), 7.35 (d, 2H, J = 8.27 Hz), 6.83-6.78 (m, 4H), 4.17 (t, 1H, J = 7.96 Hz), 3.97 (q, 4H, J = 6.76 Hz), 2.99-2.89 (m, 2H), 1.74-1.51 (m, 5H), 1.37 (t, 6H, J = 6.50 Hz). **^13^C NMR** (62.5 MHz, CDCl_3_): *δ* 157.3, 157.2, 140.1, 137.8, 126.8, 126.5, 113.9, 113.7, 64.6, 63.2, 63.1, 46.6, 26.1, 25.3, 14.7. **HRMS (MALDI-FT ICR)** m/z [M+H]^+^ calculated for C_21_H_28_NO_3_: 342.2069, found: 342.2064.

## **General procedures for the synthesis of catalysts 6e and 6f**

Catalysts **6e** and **6f** were prepared according to the literature.^[[3]](#footnote-3)^

**T1** (1.99 g, 17.37 mmol) was dissolved in MeOH (12 mL) and thionyl chloride (1.39 mL, 19.1 mmol) was added during 5 minutes at 0 °C. The reaction mixture was stirred and refluxed for one hour. Methanol was evaporated under vacuum, yielding a yellow oil, which was then dried under vacuum. The product **T2** was used in the following step without further purification.

To a cool solution (0-5 °C) of **T2** (17.3 mmol) and DIPEA (7.55 mL, 43.25 mmol) in toluene (17.3 mL), benzyl bromide (2.25 mL, 19 mmol) was slowly added. The reaction mixture was refluxed for six hours. After completion, the reaction was quenched with saturated aqueous NaHCO_3_ solution, and the product was extracted with ethyl acetate (2 x 10 mL). The combined organic phase was dried over anhydrous Na_2_SO_4_, then evaporated under vacuum to afford a brown, dense oil, which was subsequently dried in a vacuum pump. The product **T3** was used in the following step without further purification (Spectra of **T3** are consistent with those reported in the literature).^46^

A solution of compound **T3** (10.9 mmol) in THF (19.8 mL) was cooled to 0 °C, and the corresponding alkylmagnesium bromide (1M, 32.7 mmol, 32.7 mL) was added in 10 minutes. The reaction mixture was stirred overnight at room temperature (monitored by TLC, *n*-hexane/ethyl acetate 10/1, UV light and phosphomolybdic acid solution). Then, the reaction was quenched with saturated NH_4_Cl solution (30 mL). The supernatant liquid was collected, leaving behind a white precipitate, which was stirred with CH_2_Cl_2_ (3 x 20 mL), and the organic extracts were collected. The combined organic extracts, together with supernatant, were washed with brine (20 mL) and dried over anhydrous Na_2_SO_4_. The solvent was evaporated under vacuum, yielding a very dense orange oil, which was then dried under vacuum. The product **T4** was used in the following step without further purification.

Compound **T4** (8.50 mmol) was dissolved in EtOH (4 mL), and palladium, 10% wt. on activated carbon, was added. The reaction mixture was stirred under a hydrogen atmosphere at room temperature overnight (monitored by TLC, CH_2_Cl_2_/MeOH 9/1, UV light and ninhydrin solution). The solution was filtered through a Celite pad and evaporated under vacuum. The crude reaction mixture was purified by flash chromatography (CH_2_Cl_2_/MeOH from 100:0 to 90:10), affording the products **6e** and **6f**.

**(*S*)-6- (pyrrolidin-2-yl)undecan-6-ol (6e)**

Yellow solid (flash chromatography, eluent CH_2_Cl_2_/MeOH, from 100/0 to 90/10), 425.4 mg, 17% yield, **mp**: 173.6 – 178.5 °C. [α]_D_^20^ = - 21.4° (C = 0.66, CHCl_3_). **^1^H NMR** (400 MHz, CDCl_3_): *δ* 3.55 (bs, 1H), 3.42 (bs, 1H), 3.25 (bs, 1H), 2.08-1.92 (m, 3H), 1.76-1.52 (m, 7H), 1.38-1.12 (m, 10H), 0.89 (t, 6H, J = 7.12 Hz). **^13^C NMR** (62.5 MHz, CDCl_3_): *δ* 73.5, 66.6, 45.5, 36.5, 35.2, 32.1, 32.0, 25.3, 24.2, 23.0, 22.8, 22.5, 22.4, 13.9, 13.8. **HRMS (MALDI-FT ICR)** m/z [M+H]^+^ calculated for C_15_H_32_NO: 242.2484, found: 242.2483.

**(*S*)-2,8-dimethyl-5-(pyrrolidin-2-yl)nonan-5-ol (6f)**

Yellow solid (flash chromatography, eluent CH_2_Cl_2_/MeOH, from 100/0 to 90/10), 496 mg, 24% yield, **mp**: 145.6 – 149.3 °C. [α]_D_^20^ = - 20.7° (C = 0.81, CHCl_3_). **^1^H NMR** (300 MHz, CDCl_3_): *δ* 3.57-3.41 (m, 2H), 3.27-3.26 (m, 1H), 2.12-1.95 (m, 3H), 1.79-1.39 (m, 9H), 1.34-1.23 (m, 3H), 0.93 (t, 12H, J = 7.12 Hz). **^13^C NMR** (62.5 MHz, CDCl_3_): *δ* 73.5, 66.7, 45.5, 34.3, 32.9, 32.1, 31.9, 28.3, 25.3, 24.2, 22.6, 22.3. **HRMS (MALDI-FT ICR)** m/z [M+H]^+^ calculated for C_15_H_32_NO: 242.2484, found: 242.2483.

## **Procedure for the bromination of (1-(phenylsulfonyl)-1*H*-indol-3-yl)methanol**

The alcohol (10 mmol) was dissolved in anhydrous CH_2_Cl_2_ (100 mL) and was introduced into a round-bottom flask under a nitrogen atmosphere. The solution was cooled to 0 °C and PBr_3_ (1.03 mL, 11 mmol) was added dropwise. The reaction mixture was stirred for 1 hour at 0 °C and then 48 hours at room temperature, monitored by TLC, using *n*-hexane/ethyl acetate (8:2) as the eluent, and revealed with UV light and a phosphomolybdic acid solution. After completion, the reaction was quenched with water and extracted with CH_2_Cl_2_ (3 x 20 mL). The combined organic phases were dried over Na_2_SO_4_ and concentrated under vacuum to afford the pure product **U1** (70% yield).^[[4]](#footnote-4)^

## **3-(bromomethyl)-1-(phenylsulfonyl)-1*H*-indole**

Pink solid, 2.35 g, 70% yield, **mp**: 113.7 – 116.5 °C. **^1^H NMR** (300 MHz, CDCl_3_): *δ* 7.97 (d, 1H, J = 8.15 Hz), 7.88 (d, 2H, J = 7.44 Hz), 7.64-7.63 (m, 2H), 7.57-7.52 (m, 1H), 7.47-7.42 (m, 2H), 7.39-7.25 (m, 2H), 4.61 (s, 2H). **^13^C NMR** (300 MHz, CDCl_3_): *δ* 137.9, 135.2, 133.9, 129.3, 128.9, 126.7, 125.3, 124.8, 123.4, 119.8, 118.9, 113.6, 23.4. **HRMS (MALDI-FT ICR)** m/z [M+H]^+^ calculated for C_15_H_13_BrNO_2_S: 349.9850 found: 349.9860.

## **Four-step sequence for the synthesis of bis arylsolfonyl 2-substituted glycerols 3**

### **Alkylation of dimethyl malonate 1**

A solution of sodium hydride (599.7 mg, 15 mmol) in anhydrous THF (20 mL) was stirred for 10 min. at 0°C. Then, a solution of dimethyl malonate (1.27 mL, 15 mmol) in anhydrous THF (10 mL) was added dropwise under stirring at 0°C. Later, a solution of the proper substituted benzyl bromide (13.6 mmol) in anhydrous THF (20 mL) was added. The solution was stirred overnight at room temperature, monitored by TLC (using *n*-hexane/ethyl acetate (8:2) as the eluent, and revealed by UV light and a phosphomolybdic acid solution). After completion, the solution was quenched by the addition of a saturated aqueous solution of NH_4_Cl, and the organic phase was separated. The aqueous phase was extracted with ethyl acetate (2 x 80 mL). The combined organic layers were dried over anhydrous Na_2_SO_4_, filtered and concentrated under vacuum. The crude reaction mixture was purified by flash chromatography (eluent: *n*-hexane/ethyl acetate 100:0 to 90/10), affording product **1** (60-70% yield). Data for compound **1a**, **1b**, **1c**, **1e**, **1g**, **1h**, **1i**, **1j**, **1k** are consistent with those reported in literature.

**Dimethyl 2-(benzyl)malonate (1a)**


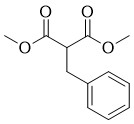


Data are consistent with those reported in the literature.^[[5]](#footnote-5)^ **^1^H NMR** (300 MHz, CDCl_3_): *δ* 7.28-7.18 (m, 5H), 3.70 (s, 6H), 3.68 (t, 1H, J = 7.90 Hz), 3.22 (d, 2H, J = 7.90 Hz).

**Dimethyl 2-(4-(*tert*-butyl)benzyl)malonate (1b)**


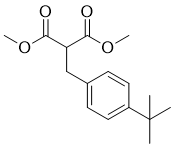


Data are consistent with those reported in the literature.^48^ **^1^H NMR** (400 MHz, CDCl_3_): *δ* 7.33-7.30 (m, 2H), 7.15-7.12 (m, 2H), 3.72 (s, 6H), 3.69 (t, 1H, J = 7.80 Hz), 3.21 (d, 2H, J = 7.80 Hz), 1.31 (s, 9H).

**Dimethyl 2-(4-chlorobenzyl)malonate (1c)**


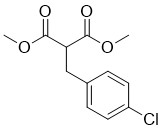


Data are consistent with those reported in the literature.^[[6]](#footnote-6)^ **^1^H NMR** (400 MHz, CDCl_3_): *δ* 7.25-7.23 (m, 2H), 7.14-7.12 (m, 2H), 3.69 (s, 6H), 3.65 (t, 1H, J = 7.60 Hz), 3.19 (d, 2H, J = 8.00 Hz).

**Dimethyl 2-(4-fluorobenzyl)malonate (1d)**


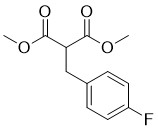


Data are consistent with those reported in the literature.^[[7]](#footnote-7)^ **^1^H NMR** (400 MHz, CDCl_3_): *δ* 7.15 (dd, 2H, J = 8.80 Hz, J = 5.30 Hz), 6.95 (dd, 2H, J = 8.80 Hz, J = 8.60 Hz), 3.68 (s, 6H), 3.63 (t, 1H, J = 7.80 Hz), 3.18 (d, 2H, J = 7.80 Hz).

**Dimethyl 2-(3-fluorobenzyl)malonate (1e)**


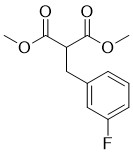


Colorless oil (flash chromatography, eluent *n*-hexane/ethyl acetate from 100/0 to 90/10), 383 mg, 85% yield. **^1^H NMR** (400 MHz, CDCl_3_): *δ* 7.31-7.26 (m, 1H), 7.02 (d, 1H, J = 7.60 Hz), 6.95 (d, 2H, J = 8.46 Hz), 3.75 (s, 6H), 3.71 (t, 1H, J = 7.58 Hz), 3.26 (d, 2H, J = 7.81 Hz). **^13^C NMR** (100 MHz, CDCl_3_): *δ* 168.9, 162.8 (d, ^1^J_C-F_ = 245.8 Hz), 140.3 (d, ^3^J_C-F_ = 6.94 Hz), 130.1 (d, ^3^J _C-F_ = 8.47 Hz), 124.5 (d, ^4^J _C-F_ = 3.08 Hz), 115.7 (d, ^2^J _C-F_ = 20.81 Hz) 113.8 (d, ^2^J _C-F_ = 20.81 Hz), 53.3, 52.6, 34.4. **^19^F NMR** (376 MHz, CDCl_3_): *δ* -113.09. **HRMS (MALDI-FT ICR)** m/z [M+Na]^+^ calculated for C_12_H_13_FO_4_Na: 263.0696, found: 263.0690.

**Dimethyl 2-(2-fluorobenzyl)malonate (1f)**


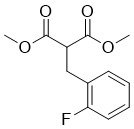


Data are consistent with those reported in the literature.^[[8]](#footnote-8)^ **^1^H NMR** (300 MHz, CDCl_3_): *δ* 7.24-7.14 (m, 2H), 7.04-6.95 (m, 2H), 3.73 (t, 1H, J = 7.80 Hz), 3.67 (s, 6H), 3.23 (d, 2H, J = 7.90 Hz).

**Dimethyl 2-(4-trifluoromethylbenzyl)malonate (1g)**

Data are consistent with those reported in the literature.^[[9]](#footnote-9)^ **^1^H NMR** (400 MHz, CDCl_3_): *δ* 7.54 (d, 2H, J = 8.10 Hz), 7.32 (d, 2H, J = 8.00 Hz), 3.71 (s, 6H), 3.68 (t, 1H, J = 7.80 Hz), 3.28 (d, 2H, J = 7.80 Hz).

**Dimethyl 2-(4-methoxybenzyl)malonate (1h)**

Data are consistent with those reported in the literature.^51^ **^1^H NMR** (400 MHz, CDCl_3_): *δ* 7.13 (d, 2H, J = 8.20 Hz), 6.84 (d, 2H, J = 8.20 Hz), 3.80 (s, 3H), 3.72 (s, 6H), 3.65 (t, 1H, J = 7.70 Hz), 3.18 (d, 2H, J = 7.80 Hz).

**Dimethyl 2-(naphthalen-1-ylmethyl)malonate (1i)**


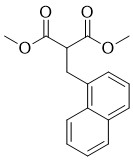


Data are consistent with those reported in the literature.^[[10]](#footnote-10)^ **^1^H NMR** (400 MHz, CDCl_3_): *δ* 7.88-7.83 (m, 3H), 7.03 (d, 1H, J = 0.70 Hz), 7.50-7.45 (m, 2H), 7.40 (dd, 1H, J = 8.50 Hz), 4.00 (t, 1H, J = 8.00 Hz), 3.60 (s, 6H), 3.26 (d, 2H, J = 7.80 Hz).

**Dimethyl 2-(3,5-dimethylbenzyl)malonate (1j)**


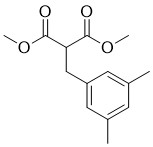


Data are consistent with those reported in the literature.^[[11]](#footnote-11)^ **^1^H NMR** (400 MHz, CDCl_3_): *δ* 6.85 (s, 1H), 6.80 (s, 2H), 3.71 (s, 6H), 3.65 (t, 1H, J = 7.80 Hz), 3.14 (d, 2H, J = 7.80 Hz), 2.27 (s, 6H).

**Dimethyl 2-(3,4,5-trifluorolbenzyl)malonate (1k)**


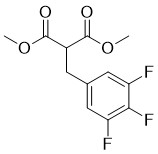


Colourless oil (flash chromatography, eluent *n*-hexane/ethyl acetate from 100/0 to 90/10), 497 mg, 72% yield. **^1^H NMR** (400 MHz, CDCl_3_): *δ* 6.83-6.80 (m, 2H), 3.71 (s, 6H), 3.59 (t, 1H, J = 7.91 Hz), 3.13 (d, 2H, J = 7.91 Hz). **^13^C NMR** (100 MHz, CDCl_3_): *δ* 168.6, 151.1 (ddd, ^1^J_C-F_ = 250.3 Hz, ^2^J_C-F_ = 10.2 Hz, J_C-F_ = 4.1 Hz), 138.8 (dt, ^1^J_C-F_ = 250.4 Hz, ^2^J_C-F_ = 15.2 Hz), 134.0 (dd, ^2^J_C-F_ = 15.2 Hz, J_C-F_ = 7.5 Hz), 113.0 (dd, ^2^J_C-F_ = 15.0 Hz, J_C-F_ = 5.7 Hz), 53.0, 52.8, 33.9. **^19^F NMR** (376 MHz, CDCl_3_): *δ* -134.16 (d, J = 20.44 Hz), -162.68 (t, J = 20.44 Hz). **HRMS (MALDI-FT ICR)** m/z [M+Na]^+^ calculated for C_12_H_11_F_3_O_4_Na: 299.0507, found: 299.0509.

**Dimethyl 2-((1-(phenylsulfonyl)-1*H*-indol-3-yl)methyl)malonte (1l)**

White wax (flash chromatography, eluent *n*-hexane/ethyl acetate from 100/0 to 80/20), 1.95 g, 70% yield. **^1^H NMR** (300 MHz, CDCl_3_): *δ* 7.97 (d, 1H, J = 7.30 Hz), 7.81 (d, 2H, J = 6.78 Hz), 7.49-7.38 (m, 5H), 7.33-7.21 (m, 2H), 3.73 (t, 1H, J = 6.96 Hz), 3.66 (s, 6H), 3.28 (d, 2H, J = 6.96 Hz).**^13^C NMR** (75 MHz, CDCl_3_): *δ* 168.9, 138.0, 135.0, 133.6, 130.2, 129.1, 126.5, 124.9, 123.9, 123.2, 119.1 118.9, 113.7, 52.6, 51.4, 24.1. **HRMS (MALDI-FT ICR)** m/z [M+K]^+^ calculated for C_20_H_19_NO_6_SK: 440.0570, found: 440.0564.

**Dimethyl 2-(pent-2-yn-1-yl)malonate (1m)**


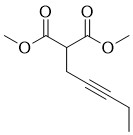


Colourless oil (flash chromatography, eluent *n*-hexane/ethyl acetate from 100/0 to 90/10), 427 mg, 79% yield. **^1^H NMR** (300 MHz, CDCl_3_): *δ* 3.73 (s, 6H), 3.52 (t, 1H, J = 7.30 Hz), 2.70 (dt, 2H, J = 7.55 Hz, J = 2.52 Hz), 2.09 (q, 2H, J = 7.25 Hz), 1.04 (t, 3H, J = 7.76 Hz). **^13^C NMR** (75 MHz, CDCl_3_): *δ* 168.4, 83.9, 74.6, 52.5, 51.4, 18.7, 13.8, 12.1. **HRMS (MALDI-FT ICR)** m/z [M+Na]^+^ calculated for C_10_H_14_O_4_Na: 221.0790, found: 221.0784.

### **Oxidation of variously substituted dimethyl 2-benzylmalonates 2**

To a solution of the proper substituted dimethyl 2-benzylmalonate **1** (5 mmol) in ethanol (10 mL), magnesium monoperoxyphthalate (2.33 g, 4 mmol) and NaHCO_3_ (420.80 mg, 5 mmol) were added. The reaction mixture was stirred overnight at room temperature, monitored by TLC using *n*-hexane/ethyl acetate (8:2) and UV light, and treated with a phosphomolybdic acid solution. After completion, the solvent was concentrated under vacuum, and the crude was dissolved in ethyl acetate. The suspension was filtered, and the organic phase was washed subsequently with NaHCO_3_ (20 mL), Na_2_SO_3_ (10% w/t, 2 x 20 mL) and water (20 mL). The organic phase was dried over anhydrous Na_2_SO_4_, filtered and concentrated under vacuum. The crude reaction mixture was purified by flash chromatography (eluent: *n*-hexane/ethyl acetate 100/0 to 80/20), affording product **2** (45-71% yield).^[[12]](#footnote-12)^

**Dimethyl 2-benzyl-2-hydroxymalonate (2a)**

Data are consistent with those reported in the literature.^55^ **^1^H NMR** (300 MHz, CDCl_3_): *δ* 7.33 – 7.15 (m, 5H), 3.80 (s, 6H), 3.71 (s, 1H), 3.35 (s, 2H).

**Dimethyl 2-(4-(*tert*-butyl)benzyl)-2-hydroxymalonate (2b)**

Colourless oil (flash chromatography, eluent *n*-hexane/ethyl acetate from 100/0 to 80/20), 454 mg, 40% yield. **^1^H NMR** (400 MHz, CDCl_3_): *δ* 7.28 (d, 2H, J = 8.16 Hz), 7.14 (d, 2H, J = 8.16 Hz), 3.80 (s, 6H), 3.69 (bs, 1H), 3.32 (s, 2H), 1.29 (s, 9H). **^13^C NMR** (100 MHz, CDCl_3_): *δ* 170.4, 150.0, 131.3, 130.0, 125.2, 79.5, 53.4, 40.4, 34.4, 31.4. **HRMS (MALDI-FT ICR)** m/z [M+Na]^+^ calculated for C_16_H_22_O_5_Na: 317.1365, found: 317.1359.

**Dimethyl 2-(4-chlorobenzyl)-2-hydroxymalonate (2c)**

Colourless oil (flash chromatography, eluent *n*-hexane/ethyl acetate from 100/0 to 80/20), 375 mg, 45% yield. **^1^H NMR** (400 MHz, CDCl_3_): *δ* 7.22 (d, 2H, J = 8.35 Hz), 7.15 (d, 2H, 8.35 Hz), 3.78 (s, 6H), 3.75 (s, 1H), 3.30 (s, 2H). **^13^C NMR** (100 MHz, CDCl_3_): *δ* 170.1, 133.2, 133.0, 131.7, 128.4, 79.2, 53.5, 40.0. **HRMS (MALDI-FT ICR)** m/z [M+Na]^+^ calculated for C_12_H_13_ClO_5_Na: 295.0349, found: 295.0369.

**Dimethyl 2-(4-fluorobenzyl)-2-hydroxymalonate (2d)**

Colourless oil (flash chromatography, eluent *n*-hexane/ethyl acetate from 100/0 to 80/20), 470.5 mg, 48% yield. **^1^H NMR** (400 MHz, CDCl_3_): *δ* 7.20-7.17 (m, 2H), 6.94 (m, 2H), 3.78 (s, 6H), 3.74 (bs, 1H), 3.31 (s, 2H). **^13^C NMR** (100 MHz, CDCl_3_): *δ* 170.2, 162.2 (d, ^1^J_C-F_ = 245.4 Hz), 131.9 (d, 2C, ^3^J_C-F_ = 7.5 Hz), 130.2 (d, J_C-F_ = 3.9 Hz), 115.1 (d, 2C, ^2^J_F_ = 21.3 Hz), 79.3, 53.4, 39.9. **^19^F NMR** (376 MHz, CDCl_3_): *δ* **–** 115.67. **HRMS (MALDI-FT ICR)** m/z [M+Na]^+^ calculated for C_12_H_13_FO_5_Na: 279.0645, found: 279.0639.

**Dimethyl 2-(3-fluorobenzyl)-2-hydroxymalonate (2e)**

Colourless oil (flash chromatography, eluent *n*-hexane/ethyl acetate from 100/0 to 80/20), 412.1 mg, 71% yield. **^1^H NMR** (400 MHz, CDCl_3_): *δ* 7.24-7.19 (m, 1H), 7.00-6.91 (m, 3H), 3.78 (s, 6H), 3.33 (s, 2H). **^13^C NMR** (100 MHz, CDCl_3_): *δ* 170.1, 162.5 (d, ^1^J_C-F_ = 245.0 Hz), 136.9 (d, ^3^J_C-F_ = 7.5 Hz), 129.5 (d, ^3^J_C-F_ = 8.5 Hz), 126.0 (d, J_C-F_ = 3.1 Hz), 117.3 (d, ^2^J_F_ = 21.6 Hz), 114.2 (d, ^2^J_F_ = 20.8 Hz), 79.2, 53.5, 40.4 (d, J_C-F_ = 1.8 Hz). **^19^F NMR** (376 MHz, CDCl_3_): *δ* - 113.52. **HRMS (MALDI-FT ICR)** m/z [M+Na]^+^ calculated for C_12_H_13_FO_5_Na: 279.0645, found: 279.0639.

**Dimethyl 2-(2-fluorobenzyl)-2-hydroxymalonate (2f)**

Colourless oil, (flash chromatography, eluent *n*-hexane/ethyl acetate from 100/0 to 80/20), 628 mg, 62% yield. **^1^H NMR** (400 MHz, CDCl_3_): *δ* 7.30-7.26 (m, 1H), 7.24-7.19 (m, 1H), 7.07-7.03 (m, 1H), 7.01-6.97 (m, 1H), 3.80 (s, 6H), 3.70 (s, 1H), 3.42 (s, 2H). **^13^C NMR** (100 MHz, CDCl_3_): *δ* 170.3, 161.4 (d, ^1^J_C-F_ = 245.8 Hz), 132.8 (d, J_C-F_ = 3.8 Hz), 129.1 (d, ^4^J_C-F_ = 8.3 Hz), 123.9 (d, J_C-F_ = 3.6 Hz), 121.4 (d, ^3^J_F_ = 15.4 Hz), 115.2 (d, ^2^J_F_ = 22.6 Hz), 78.6, 53.5, 33.7 (d, J_C-F_ = 1.5 Hz). **^19^F NMR** (376 MHz, CDCl_3_): *δ* - 118.05. **HRMS (MALDI-FT ICR)** m/z [M+Na]^+^ calculated for C_12_H_13_FO_5_Na: 279.0645, found: 279.0639.

**Dimethyl 2-(4-trifluoromethylbenzyl)-2-hydroxymalonate (2g)**

Yellow liquid (flash chromatography, eluent n-hexane/ethyl acetate from 100/0 to 85/15), 648.5 mg, 54% yield. **^1^H NMR** (400 MHz, CDCl_3_): *δ* 7.49 (d, 2H, J = 7.21 Hz), 7.33 (d, 2H, J = 7.21 Hz), 4.70, (bs, 1H), 3.75 (s, 6H), 3.37 (s, 2H). **^13^C NMR** (100 MHz, CDCl_3_): *δ* 170.1, 138.8, 130.7, 129.5 (q, ^2^J_C-F_ = 32.3 Hz) 126.8, 125.3 (q, J_C-F_ = 3.1Hz), 125.0 (q, J_C-F_ = 4.0 Hz), 124.2 (q, ^1^J_C-F_ = 271.8 Hz), 79.1, 53.4 (q, J_C-F_ = 2.8 Hz), 40.4. **^19^F NMR** (376 MHz, CDCl_3_): *δ -* 62.56. **HRMS (MALDI-FT ICR)** m/z [M+Na]^+^ calculated for C_13_H_13_F_3_O_5_Na: 329.0613, found: 329.0607.

**Dimethyl 2-(4-methoxybenzyl)-2-hydroxymalonate (2h)**

Colourless oil (flash chromatography, eluent n-hexane/ethyl acetate from 100/0 to 80/20), 1.02 g, 62% yield. **^1^H NMR** (400 MHz, CDCl_3_): *δ* 7.19 (d, 2H, J = 7.73 Hz), 6.85 (d, 2H, J = 8.63 Hz), 3.86 (s, 1H), 3.82 (s, 6H), 3.81 (s, 3H), 3.34 (s, 2H). **^13^C NMR** (100 MHz, CDCl_3_): *δ* 170.4, 158.8, 131.3, 126.4, 113.7, 79.5, 55.2, 53.3, 40.1. **HRMS (MALDI-FT ICR)** m/z [M+K]^+^ calculated for C_13_H_16_O_6_K: 307.0584, found: 307.0578.

**Dimethyl 2-(naphthalen-1-ylmethyl)-2-hydroxymalonate (2i)**

Colourless oil (flash chromatography, eluent *n*-hexane/ethyl acetate from 100/0 to 80/20), 481 mg, 55% yield. **^1^H NMR** (300 MHz, CDCl_3_): *δ* 7.81-7.71 (m, 4H), 7.47-7.37 (m, 3H), 3.81 (s, 6H), 3.54 (s, 2H). **^13^C NMR** (75 MHz, CDCl_3_): *δ* 170.2, 133.2, 132.5, 131.9, 129.0, 128.3, 127.6 (2C), 127.5, 125.8, 125.6, 79.4, 53.3, 40.9. **HRMS (MALDI-FT ICR)** m/z [M+Na]^+^ calculated for C_16_H_16_O_5_Na: 311.0895, found: 311.0889.

**Dimethyl 2-(3,5-dimethylbenzyl)-2-hydroxymalonate (2j)**

Colourless oil (flash chromatography, eluent *n*-hexane/ethyl acetate from 100/0 to 80/20), 1.92 g, 70% yield. **^1^H NMR** (300 MHz, CDCl_3_): *δ* 6.88 (s, 1H), 6.82 (s, 2H), 3.80 (s, 6H), 3.69 (s, 1H), 3.28 (s, 2H), 2.27 (s, 6H). **^13^C NMR** (75 MHz, CDCl_3_): *δ* 170.2, 137.5, 134.0, 128.8, 127.9, 79.3, 53.1, 40.6, 21.1. **HRMS (MALDI-FT ICR)** m/z [M+Na]^+^ calculated for C_14_H_18_O_5_Na: 289.1052, found: 289.1046.

**Dimethyl 2-(3,4,5-trifluorobenzyl)-2-hydroxymalonate (2k)**

Colourless oil (flash chromatography, eluent *n*-hexane/ethyl acetate from 100/0 to 80/20), 843.3 mg, 60% yield. **^1^H NMR** (400 MHz, CDCl_3_): *δ* 6.89-6.86 (m, 2H), 3.80 (s, 6H), 3.26 (s, 2H). **^13^C NMR** (100 MHz, CDCl_3_): *δ* 169.8, 150.7 (ddd, ^1^J_C-F_ = 249.5 Hz, J_C-F_ = 9.1 Hz, J_C-F_ = 3.7 Hz), 139.1 (dt, ^1^J_C-F_ = 250.8 Hz, ^2^J_C-F_ = 16.2 Hz), 130.8 (dd, ^2^J_C-F_ = 16.3 Hz, J_C-F_ = 8.5 Hz), 114.5 (dd, ^2^J_C-F_ = 14.9 Hz, J_C-F_ = 5.5 Hz), 78.9, 53.6, 39.7. **^19^F NMR** (376 MHz, CDCl_3_): *δ* - 134.9 (d, J = 20.46 Hz), - 162.38 (t, J = 20.48 Hz). **HRMS (MALDI-FT ICR)** m/z [M+Na]^+^ calculated for C_12_H_11_F_3_O_5_Na: 315.0456, found: 315.0450.

**Dimethyl 2-((1-(phenylsulfonyl)-1H-indol-3-yl)methyl)-2-hydroxymalonate (2l)**

Colourless oil (flash chromatography, eluent *n*-hexane/ethyl acetate from 100/0 to 80/20), 1.23 g, 61% yield. **^1^H NMR** (300 MHz, CDCl_3_): *δ* 7.87 (d, 1H, J = 8.66 Hz), 7.73 (d, 2H, J = 8.20 Hz), 7.49-7.32 (m, 5H), 7.26-7.12 (m, 2H), 3.63 (s, 6H), 3.36 (s, 2H). **^13^C NMR** (75 MHz, CDCl_3_): *δ* 169.9, 137.9, 134.7, 133.6, 131.1, 129.1, 126.5, 125.3, 124.6, 123.1, 119.9, 115.9, 113.4, 79.0, 53.3, 30.1. **(MALDI-FT ICR)** m/z [M+K]^+^ calculated for C_20_H_19_NO_7_SK:456.0519, found 456.0514.

**Dimethyl 2-(pent-2-yn-1-yl)-2-hydroxymalonate (2m)**

Colourless oil (flash chromatography, eluent *n*-hexane/ethyl acetate from 100/0 to 80/20), 286 mg, 49% yield. **^1^H NMR** (300 MHz, CDCl_3_): *δ* 3.95 (s, 1H), 3.81 (s, 6H), 2.91-2.90 (m, 2H), 2.12 (q, 2H, J = 8.17 Hz), 1.07 (t, 3H, J = 7.75 Hz). **^13^C NMR** (75 MHz, CDCl_3_): *δ* 169.5, 85.3, 78.0, 72.1, 53.4, 26.5, 13.9, 12.2. **HRMS (MALDI-FT ICR)** m/z [M+Na]^+^ calculated for C_10_H_14_O_5_Na: 237.0739, found: 237.0733.

### **Reduction of substituted dimethyl 2-benzyl-2-hydroxymalonates**

In a round-bottom flask under nitrogen, at 0°C, a solution of the proper substituted dimethyl-2-benzyl-2-hydroxymalonate (6.29 mmol) in anhydrous THF (6.29 mL) was added dropwise to a solution of lithium aluminum hydride (12.58 mL, 1 M in THF, 12.58 mmol). The reaction mixture was left warming at room temperature and was stirred overnight. The reaction was monitored by TLC, using *n*-hexane/ethyl acetate 1/1 as the eluent and visualized under UV light and by phosphomolybdic acid solution. After completion, the mixture was quenched by adding a solution of THF/water (5:1, 20 mL) dropwise. An aqueous solution of NaOH (2 M, 10 mL) was then added, and the reaction mixture was stirred at room temperature for 30 minutes. The solution was filtered through a Celite pad, and the ethereal filtrate was concentrated under vacuum. The product was used in the next step without further purification (60-80% yield).

### **General procedure for the protection of 2-substituted glycerols for the synthesis of starting materials 3**

In a round-bottom flask under a nitrogen atmosphere, at 0°C, the appropriate sulfonyl chloride (R^2^SO_2_Cl, R^2^ = 4-ClC_6_H_4_, 3,5-CF_3_C_6_H_3_, Me, 4-MeC_6_H_4_) (20 mmol) was added to a solution of the proper 2-substituted glycerol (5 mmol) in anhydrous pyridine (16.6 mL), containing molecular sieves (3Å). The reaction mixture was stirred overnight at room temperature (monitored by TLC *n*-hexane/ethyl acetate 8:2, UV light and phosphomolybdic acid solution). After completion, the mixture was diluted with ethyl acetate, and the solid was filtered off. The filtrate was washed in sequence with an aqueous solution of HCl (3 x 20 mL), NaHCO_3_ sat. (2 x 20 mL), and water (20 mL). The organic phase was dried over anhydrous Na_2_SO_4_, filtered and concentrated under vacuum. The crude reaction mixture was purified by flash chromatography (eluent: *n*-hexane/ethyl acetate, gradient from 100/0 to 60/40), affording product **3** (20-56% yield).

**2-benzyl-2-hydroxypropane-1,3-diyl bis(4-chlorobenzenesulfonate) (3o)**

Yellow solid (flash chromatography, eluent *n*-hexane/ethyl acetate from 100/0 to 80/20), 92.8 mg, 64% yield, **mp:** 113.1 – 114.4 °C. **^1^H NMR** (400 MHz, CDCl_3_): *δ* 7.87 (d, 4H, J = 8.76 Hz), 7.60 (d, 4H, J = 8.76 Hz), 7.32-7.29 (m, 3H), 7.13-7.11 (m, 2H), 3.96 and 3.94 (ABq, 4H, J = 9.91 Hz), 2.84 (A_2_, 2H). **^13^C NMR** (62.5 MHz, CDCl_3_): *δ* 140.9, 133.4, 133.1, 130.3, 129.7, 129.3, 128.6, 127.4, 71.7, 70.2, 39.3. **HRMS (MALDI-FT ICR)** m/z [M+Na]^+^ calculated for C_22_H_20_Cl_2_O_7_S_2_Na: 552.9925, found 552.9919.

**7b**

**2-benzyl-2-hydroxypropane-1,3-diyl bis(3,5-bis(trifluoromethyl)benzenesulfonate) (3p)**

White solid (flash chromatography, eluent *n*-hexane/ethyl acetate from 100/0 to 95/5), 105 mg, 45% yield, **mp:** 81.4 – 85.4 °C. **^1^H NMR** (600 MHz, CDCl_3_): *δ* 8.24 (s, 4H), 8.09 (s, 2H), 7.18-7.15 (m, 3H), 7.04-7.01 (m, 2H), 3.95 (A_2_, 4H), 2.76 (A_2_, 2H) 2.19 (s, 1H). **^13^C NMR** (150 MHz, CDCl_3_): *δ* 138.0, 134.8, 133.4 (q, ^2^J_C-F_ = 34.9 Hz), 132.7, 130.2, 129.4, 128.9, 128.6, 128.3 (q, J_C-F_ = 3.63 Hz), 127.8, 122.2 (q, ^1^J_CF_ = 273.55 Hz), 71.8, 71.0, 39.4. **^19^F NMR** (376 MHz, CDCl_3_): *δ* -63.00. **HRMS (ESI)** m/z [M+Na]^+^ calculated for C_26_H_18_F_12_O_7_S_2_Na: 757.0200, found 757.0214.

**2-benzyl-2-hydroxypropane-1,3-diyl dimethanesulfonate (3q)**

Yellow oil (flash chromatography, eluent *n*-hexane/ethyl acetate from 100/0 to 60/40), 63 mg, 18% yield. **^1^H NMR** (300 MHz, CDCl_3_): *δ* 7.35-7.31 (m, 5H), 4.50 and 4.40 (AXq, 4H, J = 11.85 Hz), 4.15 (s, 1H), 3.36 (A_2_, 2H), 3.12 (s, 6H). **^13^C NMR** (62.5 MHz, CDCl_3_): *δ* 132.2, 130.4, 128.9, 127.9, 88.3, 69.9, 67.0, 37.6. **HRMS (ESI)** m/z [M+Na]^+^ calculated for C_12_H_16_O_7_S_2_Na: 361.0392, found 361.0391.

**2-benzyl-2-hydroxypropane-1,3-diyl bis(4-methylbenzenesulfonate) (3a)**

White solid (flash chromatography, eluent *n*-hexane/ethyl acetate from 100/0 to 80/20), 677 mg, 40% yield, **mp:** 111.9 – 113.1 °C. **^1^H NMR** (300 MHz, CDCl_3_): *δ* 7.75 (d, 4H, J = 8.25 Hz), 7.35 (d, 4H, J=8.25 Hz), 7.21-7.20 (m, 3H), 7.07-7.05 (m, 2H), 3.86 and 3.82 (ABq, 4H, J = 9.69 Hz), 2.76 (A_2_, 2H), 2.47 (s, 6H), 2.25 (bs, 1H). **^13^C NMR** (62.5 MHz, CDCl_3_): *δ* 145.2, 133.5, 131.9, 130.3, 129.9, 128.3, 127.9, 127.1, 71.6, 70.0, 39.2, 21.6. **HRMS (MALDI-FT ICR)** m/z [M+Na]^+^ calculated for C_24_H_26_O_7_S_2_Na: 513.1018, found: 513.1012.

**2-(4-(*tert*-butyl) benzyl)-2-hydroxypropane-1,3-diyl bis(4-methylbenzenesulfonate) (3b)**

White solid (flash chromatography, eluent *n*-hexane/ethyl acetate from 100/0 to 80/20), 347 mg, 26% yield, **mp:** 123.4 – 126.8 °C. **^1^H NMR** (300 MHz, CDCl_3_): *δ* 7.76 (d, 4H, J = 8.15 Hz), 7.36 (d, 4H, J = 8.85 Hz), 7.21 (d, 2H, J = 8.28 Hz), 6.97 (d, 2H, J = 8.28 Hz), 3.86 and 3.83 (ABq, 4H, J = 10.11 Hz), 2.73 (A_2_, 2H), 2.47 (s, 6H), 2.32 (bs, 1H), 1.28 (s, 9H). **^13^C NMR** (75 MHz, CDCl_3_): *δ* 150.0, 145.2, 132.0, 130.3, 130.0, 129.9, 127.9, 125.3, 71.7, 70.1, 38.7, 34.2, 31.1, 21.6. **HRMS (MALDI-FT ICR)** m/z [M+Na]^+^ calculated for C_28_H_34_O_7_S_2_Na: 569.1644, found: 569.1638.

**2-(4-chlorobenzyl)-2-hydroxypropane-1,3-diyl bis(4-methylbenzenesulfonate) (3c)**

White solid (flash chromatography, eluent *n*-hexane/ethyl acetate from 100/0 to 80/20), 156.3 mg, 38% yield, **mp:** 123.8 – 125.4 °C. **^1^H NMR** (400 MHz, CDCl_3_): *δ* 7.73 (d, 4H, J = 8.02 Hz), 7.35 (d, 4H, J = 8.02 Hz), 7.13 (d, 2H, J = 8.28 Hz), 7.00 (d, 2H, 8.28 Hz), 3.84 and 3.78 (ABq, 4H, J = 9.91 Hz), 2.72 (A_2_, 2H), 2.47 (s, 6H). **^13^C NMR** (100 MHz, CDCl_3_): *δ* 145.5, 133.2, 132.3, 131.9, 131.8, 130.1, 128.5, 128.0, 71.8, 70.1, 38.7, 21.7. **HRMS (MALDI-FT ICR)** m/z [M+Na]^+^ calculated for C_24_H_25_ClO_7_S_2_Na: 547.0628, found: 547.0622.

**2-(4-fluorobenzyl)-2-hydroxypropane-1,3-diyl bis(4-methylbenzenesulfonate) (3d)**

White solid (flash chromatography, eluent *n*-hexane/ethyl acetate from 100/0 to 80/20), 121 mg, 24% yield, **mp:** 85.4 – 88.6 °C. **^1^H NMR** (400 MHz, CDCl_3_): *δ* 7.73 (d, 4H, J = 7.82 Hz), 7.35 (d, 4H, J = 7.82 Hz), 7.05-7.01 (m, 2H), 6.88-6.84 (m, 2H), 3.84 and 3.80 (ABq, 4H, J = 9.57 Hz), 2.73 (A_2_, 2H), 2.46 (s, 6H). **^13^C NMR** (100 MHz, CDCl_3_): *δ* 162.1 (d, ^1^J_C-F_ = 250.1 Hz), 145.5, 132.1 (d, J_C-F_ = 7.8 Hz), 132.0, 130.1, 129.5 (d, J_C-F_ = 3.0 Hz), 128.1, 115.2 (d, ^2^J_C-F_ = 21.4 Hz), 71.8, 70.1, 38.6, 21.7. **^19^F NMR** (376 MHz, CDCl_3_): *δ* – 115.35. **HRMS (MALDI-FT ICR)** m/z [M+Na]^+^ calculated for C_24_H_25_FO_7_S_2_Na: 531.0923, found: 531.0917.

**2-(3-fluorobenzyl)-2-hydroxypropane-1,3-diyl bis(4-methylbenzenesulfonate) (3e)**

White solid (flash chromatography, eluent *n*-hexane/ethyl acetate from 100/0 to 80/20), 178.1 mg, 35% yield, **mp:** 85.7 – 91.2 °C. **^1^H NMR** (400 MHz, CDCl_3_): *δ* 7.74 (d, 4H, J = 7.93 Hz), 7.35 (d, 4H, J = 7.93 Hz), 7.18-7.13 (m, 1H), 6.90-6.85 (m, 2H), 6.76 (d, 1H, J = 9.74 Hz), 3.86 and 3.80 (ABq, 4H, J = 10.06 Hz), 2.75 (A_2_, 2H), 2.46 (s, 6H). **^13^C NMR** (100 MHz, CDCl_3_): *δ* 162.6 (d, ^1^J_C-F_ = 246.6 Hz), 145.5, 136.4 (d, J _C-F_ = 6.5 Hz), 131.9, 130.1, 129.8 (d, J _C-F_ = 6.9 Hz), 128.0, 126.2, 117.4 (d, ^2^J _C-F_ = 21.6 Hz), 114.2 (d, ^2^J _C-F_ = 25.4 Hz), 71.9, 70.1, 39.1, 21.7. **^19^F NMR** (376 MHz, CDCl_3_): *δ* - 112.77. **HRMS (MALDI-FT ICR)** m/z [M+Na]^+^ calculated for C_24_H_25_FO_7_S_2_Na: 531.0923, found: 531.0917.

**2-(2-fluorobenzyl)-2-hydroxypropane-1,3-diyl bis(4-methylbenzenesulfonate) (3f)**

White solid (flash chromatography, eluent *n*-hexane/ethyl acetate from 100/0 to 80/20), 525 mg, 56% yield, **mp:** 105.6 – 107.9 °C. **^1^H NMR** (400 MHz, CDCl_3_): *δ* 7.74 (d, 4H, J = 8.05 Hz), 7.34 (d, 4H, J = 8.05 Hz), 7.22-7.18 (m, 1H), 7.12 (t, 1H, J = 7.19 Hz), 7.00-6.94 (m, 2H), 3.90 (A_2_, 4H), 2.85 (A_2_, 2H), 2.50 (s, 1H), 2.45 (s, 6H). **^13^C NMR** (100 MHz, CDCl_3_): *δ* 161.4 (d, ^1^J_C-F_ = 245.9 Hz), 145.4, 132.9 (d, J _C-F_ = 4.6 Hz), 132.1, 130.1, 129.2 (d, ^3^J _C-F_ = 7.7 Hz), 128.1, 124.2 (d, J _C-F_ = 3.8 Hz), 121.1 (d, ^2^J_F_ = 15.4 Hz), 115.4 (d, ^2^J_F_ = 22.3 Hz), 72.3, 70.6, 32.7, 21.7. **^19^F NMR** (376 MHz, CDCl_3_): *δ -* 115.78. **HRMS (MALDI-FT ICR)** m/z [M+Na]^+^ calculated for C_24_H_25_FO_7_S_2_Na: 531.0923, found: 531.0917.

**2-(4-trifluoromethylbenzyl)-2-hydroxypropane-1,3-diyl bis (4-methylbenzenesulfonate) (3g)**

White solid (flash chromatography, eluent *n*-hexane/ethyl acetate from 100/0 to 70/30), 455.2 mg, 40% yield, **mp:** 102.6 – 107.7 °C. **^1^H NMR** (400 MHz, CDCl_3_): *δ* 7.73 (d, 4H, J = 7.89 Hz), 7.39 (d, 2H, J = 7.89 Hz), 7.35 (d, 4H, J = 7.89 Hz), 7.18 (d, 2H, J = 7.89 Hz), 3.86 and 3.78 (AXq, 4H, J = 10.28 Hz), 2.81 (A_2_, 2H), 2.47 (s, 6H). **^13^C NMR** (100 MHz, CDCl_3_): *δ* 145.6, 138.1, 131.8, 130.9, 130.2, 129.4 (q, ^2^J_C-F_ = 31.7 Hz), 128.1, 125.1 (q, J_C-F_ = 4.1 Hz), 124.1 (q, ^1^J_C-F_ = 272.9 Hz), 71.9, 70.1, 39.2, 21.7. **^19^F NMR** (376 MHz, CDCl_3_): *δ* - 62.56. **HRMS (MALDI-FT ICR)** m/z [M+K]^+^ calculated for C_25_H_25_F_3_O_7_S_2_K: 597.0631, found: 597.0625.

**2-(4-methoxybenzyl)-2-hydroxypropane-1,3-diyl bis(4-methylbenzenesulfonate) (3h)**

White wax (flash chromatography, eluent n-hexane/ethyl acetate from 100/0 to 80/20), 845.7 mg, 64% yield. **^1^H NMR** (400 MHz, CDCl_3_): *δ* 7.75 (d, 4H, J = 7.40 Hz), 7.35 (d, 4H, J = 7.40 Hz), 6.97 (d, 2H, J = 7.19 Hz), 6.74 (d, 2H, J = 7.19 Hz), 3.84 and 3.82 (ABq, 4H, J = 9.77 Hz), 3.77 (s, 3H), 2.71 (A_2_, 2H), 2.47 (s, 6H). **^13^C NMR** (100 MHz, CDCl_3_): *δ* 158.9, 145.4, 132.1, 131.5, 130.1, 128.1, 125.5, 113.9, 71.9, 70.2, 55.2, 38.5, 21.7. **HRMS (MALDI-FT ICR)** m/z [M+K]^+^ calculated for C_25_H_28_O_8_S_2_K: 559.0863, found: 559.0857.

**2-(naphthalen-1-ylmethyl)-2-hydroxypropane-1,3-diyl bis(4-methylbenzenesulfonate) (3i)**

White solid (flash chromatography, eluent *n*-hexane/ethyl acetate from 100/0 to 80/20), 130 mg, 24% yield, **mp:** 128.6 – 134.0 °C. **^1^H NMR** (300 MHz, CDCl_3_): *δ* 7.80-7.66 (m, 7H), 7.55 (s, 1H), 7.48-7.45 (m, 2H), 7.30 (d, 4H, J = 8.22 Hz), 7.24-7.21 (m, 1H), 3.89 and 3.87 (ABq, 4H, J = 10.36 Hz), 2.95 (A_2_, 2H), 2.44 (s, 6H). **^13^C NMR** (75 MHz, CDCl_3_): *δ* 145.2, 133.1, 132.3, 131.8, 131.2, 129.9, 129.1, 128.3, 127.9 (2C), 127.5, 127.4, 126.0, 125.8, 71.9, 70.1, 39.4, 21.6. **HRMS (MALDI-FT ICR)** m/z [M+Na]^+^ calculated for C_28_H_28_O_7_S_2_Na: 563.1174, found: 563.1168.

**2-(3,5-dimethylbenzyl)-2-hydroxypropane-1,3-diyl bis(4-methylbenzenesulfonate) (3j)**

Yellow solid (flash chromatography, eluent *n*-hexane/ethyl acetate from 100/0 to 80/20), 631.8 mg, 40% yield, **mp:** 111.5 – 115.2 °C. **^1^H NMR** (300 MHz, CDCl_3_): *δ* 7.75 (d, 4H, J = 8.87 Hz), 7.35 (d, 4H, J = 8.87 Hz), 6.87 (s, 1H), 6.70 (s, 2H), 3.86 (A_2_, 4H), 2.70 (A_2_, 2H), 2.46 (s, 6H), 2.23 (s, 6H), 2.14 (s, 1H). **^13^C NMR** (75 MHz, CDCl_3_): *δ* 145.1, 138.1, 133.2, 132.1, 129.9, 128.9, 128.1, 127.9, 71.6, 70.1, 39.0, 21.6, 21.1. **HRMS (MALDI-FT ICR)** m/z [M+H]^+^ calculated for C_26_H_31_O_7_S_2_: 519.1511, found: 519.1506.

**2-(3,4,5-fluorobenzyl)-2-hydroxypropane-1,3-diyl bis (4-methylbenzenesulfonate) (3k)**

White solid (flash chromatography, eluent *n*-hexane/ethyl acetate from 100/0 to 80/20), 329.1 mg, 31% yield, **mp:** 138.5 – 141.5 °C. **^1^H NMR** (400 MHz, CDCl_3_): *δ* 7.75 (d, 4H, J = 7.39 Hz), 7.37 (d, 4H, J = 7.39 Hz), 6.71-6.2 (m, 2H), 3.89 and 3.77 (AXq, 4H, J = 9.30 Hz), 2.76 (s, 1H), 2.68 (A_2_, 2H), 2.47 (s, 6H). **^13^C NMR** (100 MHz, CDCl_3_): *δ* 150.6 (ddd, ^1^J_C-F_ = 253.9 Hz, ^2^J_C-F_ = 10.1 Hz, J_C-F_ = 3.4 Hz), 145.8, 138.9 (dt, ^1^J_C-F_ = 250.9, ^2^J_C-F_ = 15.1 Hz), 131.7, 130.2, 128.0, 114.6 (dd, ^2^J_C-F_ = 15.3 Hz, ^3^J_C-F_ = 6.2 Hz), 113.4 (dd, ^2^J_C-F_ = 17.6 Hz, ^3^J_C-F_ = 7.4 Hz), 71.8, 69.9, 38.6, 21.7. **^19^F NMR** (376 MHz, CDCl_3_): *δ* - 134.44 (t, J = 13.97 Hz), - 162.57 (d, J = 8.55 Hz). **HRMS (MALDI-FT ICR)** m/z [M+K]^+^ calculated for C_24_H_23_F_3_O_7_S_2_K: 583.0474, found: 583.0469.

**3-((1-(phenylsulfonyl)-1*H*-indol-3-yl)methyl)-1,5-ditosylpentan-3-ol (3l)**

Yellow wax (flash chromatography, eluent *n*-hexane/ethyl acetate from 100/0 to 80/20), 340.6 mg, 34% yield. **^1^H NMR** (300 MHz, CDCl_3_): *δ* 8.02-7.97 (m, 1H), 7.90-7.86 (m, 2H), 7.73-7.70 (m, 2H), 7.49-7.44 (m, 4H), 7.36-7.29 (m, 7H), 7.26-7.21 (m, 2H), 3.92 and 3.88 (ABq, 4H, J = 10.13 Hz), 2.89 (A_2_, 2H), 2.48 (s, 6H). **^13^C NMR** (75 MHz, CDCl_3_): *δ* 145.4, 137.8, 134.7, 133.7, 131.6, 129.9 (2C), 129.2, 127.8 (2C), 126.6, 125.5, 124.8, 123.2, 119.8, 113.4, 72.0, 70.1, 28.7, 21.6. **(HRMS (MALDI-FT ICR)** m/z [M+K]^+^ calculated for C_32_H_31_NO_9_S_3_K: 708.0798, found: 708.0792.

**2-hydroxy-2-(pent-2-yn-1-yl)propane-1,3-diyl bis (4-methylbenzenesulfonate) (3m)**

White solid (flash chromatography, eluent *n*-hexane/ethyl acetate from 100/0 to 80/20), 930.1 mg, 51% yield, **mp:** 66.7 – 70.3 °C. **^1^H NMR** (400 MHz, CDCl_3_): *δ* 7.75 (d, 4H, J = 8.50 Hz), 7.34 (d, 4H, J = 8.50 Hz), 3.97 (A_2_, 4H), 2.62 (bs, 1H), 2.44 (s, 6H), 2.38 (s, 2H), 2.03 (q, 2H, J = 6.80 Hz), 1.01 (t, 3H, J = 6.82 Hz). **^13^C NMR** (100 MHz, CDCl_3_): *δ* 145.3, 132.2, 130.0, 128.1, 86.6, 71.7, 71.3, 70.4, 25.3, 21.7, 13.8, 12.3. **HRMS (MALDI-FT ICR)** m/z [M+K]^+^ calculated for C_22_H_26_O_7_S_2_K: 505.0757, found: 505.0751.

## **Synthesis of 2-hydroxy-2-phenylpropane-1,3-diyl bis (4-methylbenzenesulfonate) 3n**

According to the literature, *p*-toluenesulfonic acid (78.0 mg, 0.41 mmol) and 2,2-dimethoxypropane (1.49 mL, 12.2 mmol) were added to a solution of tris(hydroxymethyl)aminomethane hydrochloride (TRIS^.^HCl) (1.61 g, 10.2 mmol) in DMF (3.20 mL). The resulting solution was stirred at room temperature for 18 hours. Triethylamine (80 μL, 5 mol%) was then added to the mixture, and the solvent was evaporated under reduced pressure. The viscous crude was then dissolved in triethylamine (1.2 mL), and EtOAc (24 mL) was added. The white precipitate of ammonium salt was filtered off after stirring for 30 minutes. The filtrate was evaporated under reduced pressure to give **Z1** as a colourless liquid (1.6 g, 9.92 mmol, 97% yield). A solution of NaIO_4_ (2.71 g, 12.7 mmol) in water (38 mL) was added dropwise to a cold solution (5°C) containing **Z1** (12.7 mmol) and KH_2_PO_4_ (1.73 g, 12.7 mmol) in water (41 mL). The mixture was stirred at 5 °C for 3.5 hours and then at room temperature overnight. Finally, Na_2_S_2_O_3_ (2.00 g, 12.7 mmol) was added, and the resulting solution was stirred for an additional 15 minutes. Then, the mixture was extracted with (5 x 25 mL). The combined organic layers were dried over Na_2_SO_4_, concentrated under reduced pressure, and purified by flash chromatography (CH_2_Cl_2_/Et_2_O 99:1) to afford **Z2** as a colourless volatile liquid (1.09 g, 8.42 mmol, 67% yield).^[[13]](#footnote-13)^

A solution of the ketone **Z2** (5.6 mmol) in THF (12 mL) was added, through a cannula at 0 °C, to a solution of phenylmagnesium bromide (1 M in THF, 16.8 mmol) in THF (16 mL). The reaction was stirred at 0 °C for 1 hour and then quenched with a saturated aqueous solution of NH_4_Cl. Product **Z3** was extracted with Et_2_O (3 x 20 mL). The combined organic phases were dried over anhydrous Na_2_SO_4_, filtered and concentrated under vacuum. The crude reaction mixture was purified by flash chromatography using gradients of *n*-hexane and EtOAc (from 100:0 to 95:5), which yielded the product **Z3** as a colourless liquid (470 mg, 2.25 mmol, 40% yield).

Finally, concentrated HCl (four drops) was added to a solution of acetonide **Z3** (2.25 mmol) in MeOH:H_2_O 2:1 (13.15 mL). The mixture was stirred at room temperature for 6 hours. Evaporation of the volatile materials under vacuum, followed by chromatographic purification of the crude mixture using CH_2_Cl_2_ containing 5% MeOH, yielded pure triol (354.1 mg, 2.1 mmol, 93% yield).^[[14]](#footnote-14)^

In a round-bottom flask under a nitrogen atmosphere, at 0 °C, tosyl chloride (1.60 g, 8.4 mmol) was added to a solution of 2-phenylpropane-1,2,3-triol (2.1 mmol) in anhydrous pyridine (7 mL) containing molecular sieves (3Å) and the reaction mixture was stirred overnight at room temperature. After completion (checked by TLC, using *n*-hexane/ethyl acetate 8/2 as eluent, visualised by UV light and phosphomolybdic acid solution), the mixture was diluted with ethyl acetate, and the solid was filtered off. The filtrate was washed in sequence with aqueous solution of HCl (3 x 15 mL), NaHCO_3_ (15 mL), and water (10 ml). The organic phase was dried over anhydrous Na_2_SO_4_, filtered and concentrated under vacuum. The crude reaction mixture was purified by flash chromatography (eluent: *n*-hexane/ethyl acetate 100/0 to 60/40), affording product **3n** in 32% yield.

**2-hydroxy-2-phenylpropane-1,3-diyl bis (4-methylbenzenesulfonate) (3n)**

White solid (flash chromatography, eluent *n*-hexane/ethyl acetate from 100/0 to 80/20), 151.5 mg, 53% yield, **mp:** 105.7 – 108.3 °C. **^1^H NMR** (400 MHz, CDCl_3_): *δ* 7.66 (d, 4H, J = 7.78 Hz), 7.31-7.28 (m, 9H), 4.19 (A_2_, 4H), 2.44 (s, 6H). **^13^C NMR** (100 MHz, CDCl_3_): *δ* 145.3, 138.0, 132.0, 130.0, 128.6, 128.5, 128.0, 125.5, 73.8, 72.3, 21.7. **HRMS (MALDI-FT ICR)** m/z [M+Na]^+^ calculated for C_23_H_24_O_7_S_2_Na: 499.0861, found: 499.0855.

## **General procedure for the synthesis of racemic epoxides 4**

In a round-bottom flask, potassium carbonate (13.8 mg, 0.1 mmol) was added to a solution of the proper arylsulfonyl 2-substituted glycerol **3** (0.05 mmol) in acetonitrile (0.25 mL). The reaction was stirred at 40°C overnight, monitored by TLC (using *n*-hexane/ethyl acetate (8:2) as the eluent, and visualised by UV light and a phosphomolybdic acid solution). After completion, the crude product was purified by flash chromatography (eluent: *n*-hexane/ethyl acetate, 100/0 to 90/10) to isolate the racemic epoxide **4**.

## **Table S1.** Screening of the protecting group^a^

| entry | | R^2^ | t (d) | yield **4**(%) | ee **4**(%)^b^ | **4** | |
| --- | --- | --- | --- | --- | --- | --- | --- |
| 1 | | 4-MeC_6_H_4_ | 3 | 26 | 58 | **4a** | |
| 2 | | 4-ClC_6_H_4_ | 5 | 38 | 31 | **4o** | |
| 3 | | 3,5-(CF_3_)_2_C_6_H_3_ | 3.5 | 30 | 32 | **4p** | |
| 4 | | Me | 5 | 11 | 17 | **4q** | |
|  | ^a^ *Reaction conditions*: **3** (0.1 mmol), **6a** (20 mol%) and NaHCO_3_ (0.1 mmol) in anhydrous toluene. ^b^ HPLC analysis on a chiral stationary phase. | | | | | |  |

## **Table S2.** Solvent screening^a^

| entry | solvent | conv (%)^b^ | ee (%)^c^ |
| --- | --- | --- | --- |
| 1 | chloroform | 39 | 43 |
| 2 | *m*-xylene | 11 | 73 |
| 3 | trifluorotoluene | 27 | 70 |
| 4 | esafluorobenzene | 7 | 60 |
| 5 | 2-MeTHF | 10 | 40 |
| 6 | toluene | 45 | 76 |
| ^a^ *Reaction conditions*: **3a** (0.1 mmol), **6a** (20 mol%), **9a** (20 mol%) and NaHCO_3_ (0.1 mmol). ^b^ Yield determined by ^1^H NMR analysis of crude reaction mixture. ^c^ HPLC analysis on a chiral stationary phase. | | | |

## **Graphic S1.** Study of the desymmetrization process over seven days^a, b, c^

**
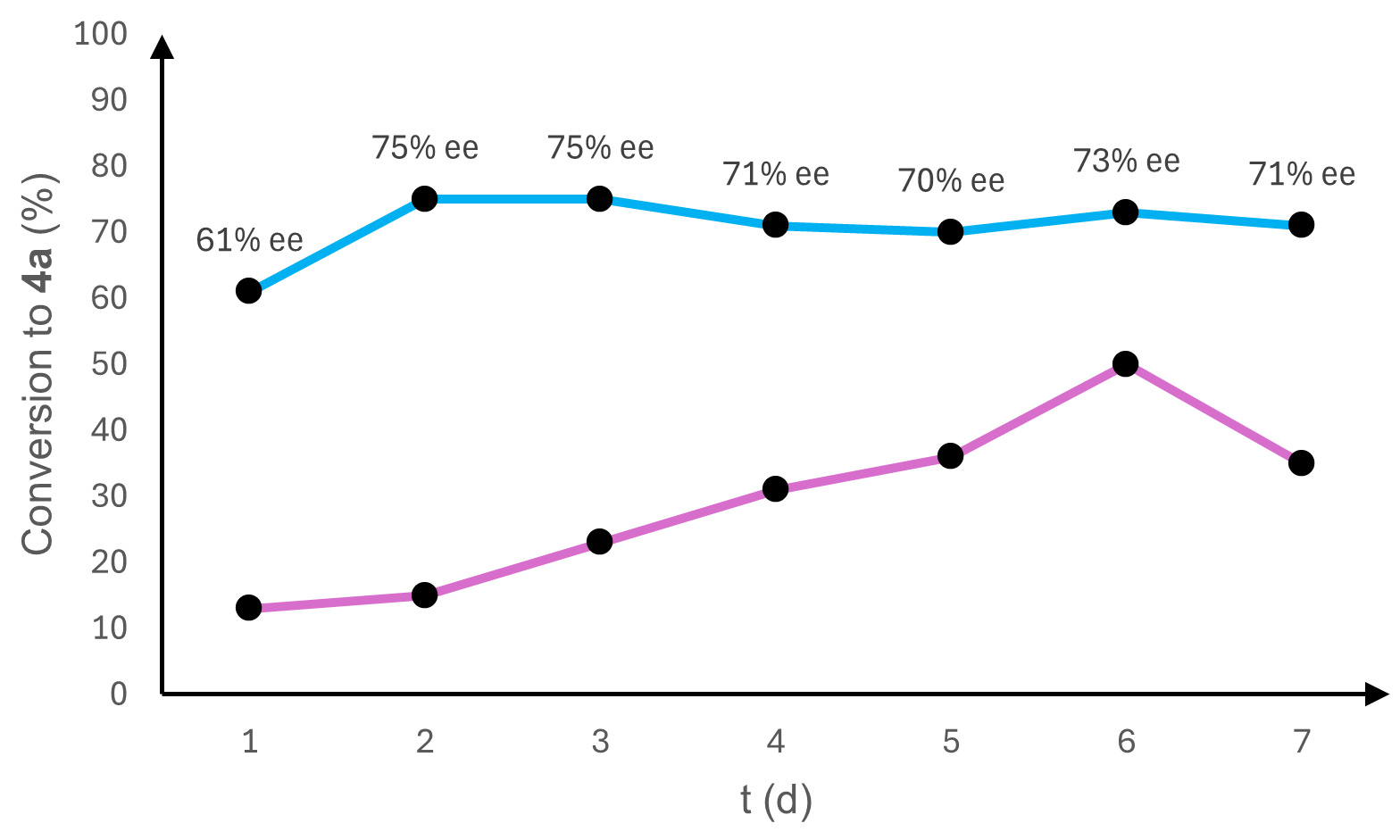
**

^a^ Data reported for each time value are referred to individual reaction. *Reaction conditions*: **3a** (0.1 mmol), **6a** (20 mol%), **9a** (20 mol%) and NaHCO_3_ (0.1 mmol) in toluene (500 μL). ^b^ Yield determined by ^1^H NMR analysis of crude reaction mixture. ^c^ HPLC analysis on a chiral stationary phase.

## **General procedure for the synthesis of enantioenriched epoxides 4o, 4p, 4q**

In a round-bottom flask, sodium bicarbonate (8.4 mg, 0.1 mmol) and **6a** (5.1 mg, 0.02 mmol) were added to a solution of the proper bis-sulfonyl 2-benzyl glycerol **3** (0.1 mmol) in toluene (0.5 mL). The reaction was stirred at room temperature for 5-7 days (monitored by TLC, using *n*-hexane/ethyl acetate 8:2 as the eluent, visualized by UV light and phosphomolybdic acid solution). After completion, the crude mixture was purified by flash chromatography (eluent: *n*-hexane/diethyl ether from 100/0 to 90/10) to afford enantioenriched epoxide **4o, 4p** or **4q**.

**2-benzyloxiran-2-yl 4-chlorobenzenesulfonate (4o)**

White wax (flash chromatography, eluent *n*-hexane/ethyl acetate from 100/0 to 90/10), 12.5 mg, 38% yield. [α]^27^ = + 4.6° (C = 0.61, CHCl_3_), 36% ee. **^1^H NMR (**250 MHz, CDCl_3_): *δ* 7.82 (d, 2H, J = 8.39 Hz), 7.52 (d, 2H, J = 8.39 Hz), 7.26-7.24 (m, 3H), 7.13-7.11 (m, 2H), 4.02 and 3.94 (ABq, 2H, J = 10.96 Hz), 3.00 and 2.88 (ABq, 2H, J = 14.68 Hz), 2.69 and 2.67 (ABq, 2H, J = 4.64 Hz). **^13^C NMR** (62.5 MHz, CDCl_3_): *δ* 140.6, 134.9, 134.0, 129.5, 129.4, 129.3, 128.5, 126.9, 71.5, 57.0, 50.3, 37.4. **HRMS (MALDI-FT ICR)** m/z [M+Na]^+^ calculated for C_16_H_15_ClO_4_SNa: 361.0277, found: 361.0272. **HPLC** (AD-H, 2-propanol/*n*-hexane = 2/98, rate = 1.0 mL/min, λ = 220 nm) t_R_ = 33.0 min (minor), 35.4 min (major).

**(2-benzyloxiran-2-yl)methyl 3,5-bis(trifluoromethylbenzenesulfonate (4p)**

White solid (flash chromatography, eluent n-hexane/ethyl acetate from 100/0 to 90/10), 13.2 mg, 30% yield, **mp:** 61.1 – 63.5 °C. [α]^27^ = - 3.1° (C = 0.62, CHCl_3_), 30% ee. **^1^H NMR** (600 MHz, CDCl_3_): *δ* 8.26 (s, 2H), 8.08 (s, 1H), 7.19-7.15 (m, 3H), 7.04 (d, 2H, J = 6.95 Hz), 4.13 and 3.97 (AX, 2H, J = 12.20 Hz), 2.95 and 2.79 (AX, 2H, J = 14.23 Hz), 2.66 and 2.64 (ABq, 2H, J = 4.77 Hz). **^13^C NMR** (150 MHz, CDCl_3_): *δ* 138.7, 134.8, 133.2 (q, ^2^J_C-F_ = 34.8 Hz), 129.4, 128.7, 128.2 (q, J_C-F_ = 3.6 Hz), 127.5 (q, J_C-F_ = 3.8 Hz), 127.2, 122.2 (q, ^1^J_C-F_ = 273.5 Hz), 72.6, 57.0, 50.4, 37.6. **^19^F NMR** (376 MHz, CDCl_3_): *δ* - 62.94. (**HRMS (MALDI-FT ICR)** m/z [M+K]^+^ calculated for C_18_H_14_F_6_O_4_SK: 479.0154, found: 479.0148. **HPLC** (IC, 2-propanol/*n*-hexane = 10/90, rate = 1.0 mL/min, λ = 220 nm) t_R_ = 5.3 min (major), 6.2 min (minor).

**(2-benzyloxiran-2-yl)methyl methanesulfonate (4q)**

White wax (flash chromatography, eluent *n*-hexane/ethyl acetate from 100/0 to 90/10), 6.5 mg, 26% yield. [α]^22^ = - 7.2° (C = 0.69, CHCl_3_), 58% ee. **^1^H NMR** (600 MHz, CDCl_3_): *δ* 7.26-7.24 (m, 2H), 7.21-7.16 (m, 3H), 4.19 and 4.03 (AX, 2H, J = 9.97 Hz), 3.05 and 2.87 (AX, 2H, J = 15.25 Hz), 2.96 (s, 3H), 2.75 and 2.69 (AX, 2H, J = 4.24 Hz). **^13^C NMR** (150 MHz, CDCl_3_): *δ* 135.1, 129.6, 128.7, 127.2, 70.8, 57.4, 50.4, 37.8, 37.7. **HRMS (MALDI-FT ICR)** m/z [M+Na]^+^ calculated for C_11_H_14_O_4_SNa: 265.0510, found: 265.0505. **HPLC** (AS-H, 2-propanol/*n*-hexane = 20/80, rate = 0.8 mL/min, λ = 220 nm) t_R_ = 23.5 min (major), 26.4 min (minor).

## **General procedure for the synthesis of enantioenriched epoxides 4a-n**

In a round-bottom flask, sodium bicarbonate (8.4 mg, 0.1 mmol), **6a** (5.1 mg, 0.02 mmol) and **9a** (9.3 mg, 0.02 mmol) were added to a solution of the proper bis-tosylated 2-substituted glycerol **3** (0.1 mmol) in toluene (0.5 mL). The reaction was stirred at room temperature for 5-9 days (monitored by TLC, using *n*-hexane/ethyl acetate 8/2 as eluent, visualized by UV light and phosphomolybdic acid solution). After completion, the crude mixture was purified by flash chromatography (eluent: *n*-hexane/diethyl ether, gradient from 100/0 to 90/10) to isolate the enantioenriched epoxides **4a-n**. The synthesis of (*S*)-**4b** and (*S*)-**4g** was carried out by using *ent*-**6a** and *ent*-**9a**.

## **Determination of the absolute configuration of epoxide 4a**

In a round-bottom flask under nitrogen, catalyst **6i** (179.0 mg, 0.55 mmol) and hydrogen peroxide (50% w/w, 4.10 mmol) were added to a solution of compound **11** (2.74 mmol) in toluene (5.48 mL). The reaction was stirred at room temperature overnight (monitored by TLC, *n*-hexane/ethyl acetate 9/1, UV light and phosphomolybdic acid solution). After completion, the reaction was quenched with aqueous Na_2_S_2_O_3_ and the aqueous phase was extracted with chloroform (30 mL x 3). The organic phases were combined, dried over Na_2_SO_4_, and concentrated under reduced pressure. The crude material was purified by flash chromatography (eluent: *n*-hexane/diethyl ether, 100/0 to 98/2) to afford product **12** (45% yield).^[[15]](#footnote-15)^

A solution of compound **12** (0.74 mmol) in methanol (2.96 mL) was cooled at 0 °C, and sodium borohydride (112.0 mg, 2.96 mmol) was added. The reaction was stirred at room temperature for 30 minutes (monitored by TLC using an 8:2 *n*-hexane/ethyl acetate mixture, visualised by UV light, and a phosphomolybdic acid solution). After completion, the reaction was quenched with water and extracted with ethyl acetate (25 x 3 mL). The combined organic phases were dried over Na_2_SO_4_ and concentrated under vacuum to afford product (*R*)-**13** (80% yield) (**HPLC**: AD-H 2-propanol/*n*-hexane = 3/97, rate = 1.0 mL/min, λ = 210 nm) t_R_ = 17.1 min (minor), 19.0 min (major). [α]_D_^20^ = + 24.3° (C = 2.00, CH_2_Cl_2_), 79% ee.).^[[16]](#footnote-16)^

In a round-bottom flask under nitrogen, to a solution of epoxyalcohol (*R*)-**13** (0.12 mmol) in anhydrous dichloromethane (1.20 mL) at 0 °C, triethylamine (0.031 mL, 0.21 mmol) and *p*-toluensulfonyl chloride (26.1 mg, 0.13 mmol) were added. The reaction was stirred at 0 °C for 5 hours, monitored by TLC (eluent: *n*-hexane/ethyl acetate, 8/2, visualized by UV light, and phosphomolybdic acid solution). After completion, the reaction was diluted with water and extracted with dichloromethane (10 mL x 3). The combined organic phases were washed with brine and dried over Na_2_SO_4_. The mixture was filtered and concentrated under vacuum. The crude mixture was purified by flash chromatography (eluent: *n*-hexane/diethyl ether from 100/0 to 90/10) to afford product (*S*)-**4a** in 33% yield (**HPLC**: AS-H 2-propanol/*n*-hexane = 10/90, rate = 1.0 mL/min, λ = 220 nm t_R_ = 19.7 min (minor), 21.3 min (major); [α]_D_^20^ = + 5.0° (C = 0.83, CHCl_3_), 79% ee.).^[[17]](#footnote-17)^ Comparison of the optical rotation of epoxide (*S*)-**4a** obtained using catalyst **6i** with that of **4a** synthesized using **6a**/**9a** catalytic system ([α]_D_^21^ = - 5.1° (C = 0.84, CHCl_3_), 72% ee) allows the assignment of the absolute configuration as (*R*). The absolute configurations of the epoxides **4b-h** and **4j**,**k** were assigned by analogy.

**(*R*)-(2-benzyloxiran-2-yl)methyl 4-methylbenzenesulfonate (4a)**

White solid (flash chromatography, eluent *n*-hexane/diethyl ether from 100/0 to 90/10), 13 mg, 40% yield, **mp:** 89.7 – 92.3 °C. [α]_D_^21^ = - 5.1° (C = 0.84, CHCl_3_), 72% ee. **^1^H NMR** (300 MHz, CDCl_3_): *δ* 7.80 (d, 2H, J = 8.48 Hz), 7.37 (d, 2H, J = 8.48 Hz), 7.29-7.26 (m, 3H), 7.16-7.13 (m, 2H), 3.98 and 3.95 (ABq, 2H, J = 13.7 Hz), 3.01 and 2.92 (ABq, 2H, J = 10.68 Hz), 2.71 and 2.67 (ABq, 2H, J = 14.05 Hz), 2.49 (s, 3H). **^13^C NMR**: (75 MHz, CDCl_3_): *δ* 144.9, 135.0, 132.5, 129.8, 129.5, 128.4, 127.8, 126.9, 71.2, 57.0, 50.3, 37.4, 21.5. **HRMS (MALDI-FT ICR)** m/z [M+K]^+^ calculated for C_17_H_18_O_4_SK: 357.0563, found: 357.0557. **HPLC** (AS-H, 2-propanol/*n*-hexane = 10/90, rate = 1.0 mL/min, λ = 220 nm) t_R_ = 19.9 min (major), 22.2 min (minor).

**(*R*)-(2-(4-(*tert*-butyl)benzyl)oxiran-2-yl)methyl 4-methylbenzenesulfonate (4b)**

White solid (flash chromatography, eluent *n*-hexane/diethyl ether from 100/0 to 90/10), 19 mg, 51% yield, **mp:** 99.6 – 103.8 °C. [α]_D_^20^ = - 7.6° (C = 0.89, CHCl_3_), 67% ee. **^1^H NMR** (400 MHz, CDCl_3_): *δ* 7.78 (d, 2H, J = 8.98 Hz), 7.34 (d, 2H, J = 8.98 Hz), 7.25 (d, 2H, J = 8.16 Hz), 7.03 (d, 2H, J = 8.16 Hz), 3.97 and 3.93 (ABq, 2H, J = 10.08 Hz), 2.95 and 2.83 (AX, 2H, J = 14.40 Hz), 2.66 (A_2_, 2H), 2.46 (s, 3H), 1.29 (s, 9H). **^13^C NMR** (100 MHz, CDCl_3_): *δ* 149.9, 145.0, 132.7, 132.1, 129.9, 129.3, 128.1, 125.4, 71.4, 57.3, 50.5, 37.1, 34.5, 31.4, 21.7. **HRMS (MALDI-FT ICR)** m/z [M+K]^+^ calculated for C_21_H_26_O_4_SK: 413.1189, found: 413.1186. **HPLC** (AD-H, 2-propanol/*n*-hexane = 10/90, rate = 1.0 mL/min, λ = 230 nm) t_R_ = 7.2 min (minor), 8.1 min (major).

**(*S*)-(2-(4-(*tert*-butyl)benzyl)oxiran-2-yl)methyl 4-methylbenzenesulfonate (4b)**

White solid (flash chromatography, eluent *n*-hexane/diethyl ether from 100/0 to 90/10), 28 mg, 75% yield. [α]_D_^25^ = + 4.9° (C = 0.88, CHCl_3_), 72% ee. **HPLC** (AD-H, 2-propanol/*n*-hexane = 10/90, rate = 1.0 mL/min, λ = 230 nm) t_R_ = 7.2 min (major), 8.1 min (minor).

**(*R*)-(2-(4-chlorobenzyl)oxiran-2-yl)methyl 4-methylbenzenesulfonate (4c)**

Yellow liquid (flash chromatography, eluent *n*-hexane/diethyl ether from 100/0 to 90/10), 15.9 mg, 45% yield. [α]_D_^22^ = - 9.8° (C = 0.73, CHCl_3_), 60% ee. **^1^H NMR** (400 MHz, CDCl_3_): *δ* 7.75 (d, 2H, J = 7.70 Hz), 7.34 (d, 2H, J = 7.34 Hz), 7.20 (d, 2H, J = 9.41 Hz), 7.05 (d, 2H, J = 9.41 Hz), 3.91 (A_2_, 2H), 2.92 (A_2_, 2H), 2.67 and 2.60 (AX, 2H, J = 3.70 Hz), 2.46 (s, 3H). **^13^C NMR** (62.5 MHz, CDCl_3_): *δ* 145.1, 133.4, 132.9, 132.4, 130.9, 129.8, 128.5, 127.9, 71.1, 56.7, 50.4, 36.6, 21.5. **HRMS (MALDI-FT ICR)** m/z [M+K]^+^ calculated for C_17_H_17_ClO_4_SK: 391.0173, found: 391.0167. **HPLC** (AD-H, 2-propanol/*n*-hexane = 10/90, rate = 1.0 mL/min, λ = 230 nm) t_R_ = 14.5 min (minor), 16.1 min (major).

**(*R*)-(2-(4-fluorobenzyl)oxiran-2-yl)methyl 4-methylbenzenesulfonate (4d)**

White solid (flash chromatography, eluent *n*-hexane/diethyl ether from 100/0 to 90/10), 17.4 mg, 52% yield, **mp:** 92.3 – 94.1 °C. [α]_D_^22^ = - 3.8° (C = 0.65, CHCl_3_), 63% ee. **^1^H NMR** (400 MHz, CDCl_3_): *δ* 7.76 (d, 2H, J = 8.40 Hz), 7.34 (d, 2H, J = 8.40 Hz), 7.10-7.06 (m, 2H), 6.94-6.90 (m, 2H), 3.92 (A_2_, 2H), 2.93 (A_2_, 2H), 2.67 and 2.61 (ABq, 2H, J = 4.65 Hz), 2.46 (s, 3H). **^13^C NMR** (100 MHz, CDCl_3_): *δ* 162.1 (d, ^1^J_C-F_ = 245.1 Hz), 145.2, 132.5, 131.2 (d, ^3^J_C-F_ = 7.9 Hz), 130.8 (d, J_C-F_ = 3.5 Hz), 129.9, 128.0, 115.3 (d, ^2^J_C-F_ = 21.2 Hz), 71.3, 57.1, 50.5, 36.6, 21.7. **^19^F NMR** (376 MHz, CDCl_3_): *δ* -115.7. **HRMS (MALDI-FT ICR)** m/z [M+K]^+^ calculated for C_17_H_17_FO_4_SK: 375.0469, found: 375.0463. **HPLC** (AD-H, 2-propanol/*n*-hexane = 10/90, rate = 1.0 mL/min, λ = 230 nm) t_R_ = 15.1 min (minor), 17.0 min (major).

**(*R*)-(2-(3-fluorobenzyl)oxiran-2-yl)methyl 4-methylbenzenesulfonate (4e)**

White solid (flash chromatography, eluent *n*-hexane/diethyl ether from 100/0 to 90/10), 10.2 mg, 30% yield, **mp:** 78.3 – 80.9 °C. [α]_D_^22^ = - 8.3° (C = 0.60, CHCl_3_), 60% ee. **^1^H NMR** (400 MHz, CDCl_3_): *δ* 7.76 (d, 2H, J = 7.76 Hz), 7.34 (d, 2H, J = 7.76 Hz), 7.23-7.18 (m, 1H), 6.93-6.90 (m, 2H), 6.79 (d, 1H, J = 11.01 Hz), 3.92 (A_2_, 2H), 2.94 (A_2_, 2H), 2.69 and 2.63 (AX, 2H, J = 4.73 Hz), 2.46 (s, 3H). **^13^C NMR** (100 MHz, CDCl_3_): *δ* 162.7 (d, ^1^J_C-F_ = 246.6 Hz), 145.2, 137.6 (d, ^3^J_C-F_ = 7.5 Hz), 132.5, 130.0 (d, ^3^J_C-F_ = 7.7 Hz), 129.9, 128.0, 125.4 (d, J_C-F_ = 2.7 Hz), 116.6 (d, ^2^J_C-F_ = 21.2 Hz), 114.0 (d, ^2^J_C-F_ = 20.9 Hz), 71.2, 56.9, 50.6, 37.2, 21.7. **^19^F NMR** (376 MHz, CDCl_3_): *δ* -112.9. **HRMS (MALDI-FT ICR)** m/z [M+K]^+^ calculated for C_17_H_17_FO_4_SK: 375.0469, found: 375.0463. **HPLC** (AS-H, 2-propanol/*n*-hexane = 10/90, rate = 1.0 mL/min, λ = 230 nm) t_R_ = 19.1 min (major), 21.8 min (minor).

**(*R*)-(2-(2-fluorobenzyl)oxiran-2-yl)methyl 4-methylbenzenesulfonate (4f)**

White solid (flash chromatography, eluent *n*-hexane/diethyl ether from 100/0 to 90/10), 7.1 mg, 21% yield, **mp:** 74.3 – 76.1 °C. [α]_D_^21^ = - 11.8° (C = 0.71, CHCl_3_), 70% ee. **^1^H NMR** (400 MHz, CDCl_3_): *δ* 7.78 (d, 2H, J = 8.04 Hz), 7.33 (d, 2H, J = 8.04 Hz), 7.22-7.19 (m, 1H), 7.17-7.13 (m, 1H), 7.05-6.96 (m, 2H), 4.09 and 3.91 (AX, 2H, J = 12.31 Hz), 3.10 and 2.92 (AX, 2H, J = 15.15 Hz), 2.65 and 2.63 (ABq, 2H, J = 16.10 Hz), 2.45 (s, 3H). **^13^C NMR** (100 MHz, CDCl_3_): *δ* 161.3 (d, ^1^J_C-F_ = 245.4 Hz), 145.1, 132.6, 132.1 (d, J_C-F_ = 4.2 Hz), 129.9, 129.0 (d, J_C-F_ = 8.1 Hz), 128.0, 124.2 (d, J_C-F_ = 3.5 Hz), 122.0 (d, ^3^J_C-F_ = 15.4 Hz), 115.4 (d, ^2^J_C-F_ = 23.1 Hz), 71.5, 56.5, 50.2, 30.7, 21.7. **^19^F NMR** (376 MHz, CDCl_3_): *δ* -116.8. **HRMS (MALDI-FT ICR)** m/z [M+K]^+^ calculated for C_17_H_17_FO_4_SK: 375.0469, found: 375.0463. **HPLC** (OD-H, 2-propanol/*n*-hexane = 10/90, rate = 1.0 mL/min, λ = 228 nm) t_R_ = 10.6 min (major), 11.4 min (minor).

**(*R*)-2-(4-(trifluoromethyl)benzyl)oxiran-2-yl 4-methylbenzenesulfonate (4g)**

White wax (flash chromatography, eluent *n*-hexane/diethyl ether from 100/0 to 90/10), 29.7 mg, 80% yield. [α]_D_^21^ = - 14.8° (C = 0.78, CHCl_3_), 61% ee. **^1^H NMR** (400 MHz, CDCl_3_): *δ* 7.75 (d, 2H, J = 7.27 Hz), 7.48 (d, 2H, J = 7.27 Hz), 7.33 (d, 2H, J = 7.27 Hz), 7.24 (d, 2H, J = 7.27 Hz), 3.91 (A_2_, 2H), 3.02 and 2.99 (ABq, 2H, J = 15.45 Hz), 2.68 and 2.61 (AX, 2H, J = 4.41 Hz), 2.45 (s, 3H). **^13^C NMR** (100 MHz, CDCl_3_): *δ* 145.3, 139.3, 132.4, 130.1, 130.0, 129.3 (q, ^2^J_C-F_ = 32.6 Hz), 128.0, 125.4 (q, J_C-F_ = 3.9 Hz), 124.1 (q, ^1^J_C-F_ = 271.5 Hz), 71.2, 56.8, 50.6, 37.2, 21.6. **^19^F NMR** (376 MHz, CDCl_3_): *δ* - 62.49. **HRMS (MALDI-FT ICR)** m/z [M+K]^+^ calculated for C_18_H_17_F_3_O_4_SK: 425.0437, found: 425.0432. **HPLC** (AD-H, 2-propanol/*n*-hexane = 10/90, rate 1.0 mL/min, λ = 230 nm) t_R_ = 12.6 min (minor), 14.3 min (major).

**(*S*)-2-(4-(trifluoromethyl)benzyl)oxiran-2-yl 4-methylbenzenesulfonate (4g)**

White wax (flash chromatography, eluent *n*-hexane/diethyl ether from 100/0 to 90/10), 32.5 mg, 87% yield. [α]_D_^23^ = + 12.7° (C = 0.78, CHCl_3_), 60% ee. **HPLC** (AD-H, 2-propanol/*n*-hexane = 10/90, rate 1.0 mL/min, λ = 230 nm) t_R_ = 11.8 min (major), 13.1 min (minor).

**(2-(naphtalen-1-ylmethyl)oxiran-2-yl)methyl 4-methylbenzenesulfonate (4i)**

White solid (flash chromatography, eluent *n*-hexane/diethyl ether from 100/0 to 90/10), 8.0 mg, 22% yield, **mp:** 98.0 – 100.5 °C. [α]_D_^22^ = + 4.3° (C = 0.51, CHCl_3_), 53% ee. **^1^H NMR** (400 MHz, CDCl_3_): *δ* 7.81-7.78 (m, 1H), 7.74-7.72 (m, 4H), 7.58 (s, 1H), 7.49-7.45 (m, 2H), 7.28-7.25 (m, 3H), 3.96 (A_2_, 2H), 3.14 and 3.08 (AX, 2H, J = 14.64 Hz), 2.71 and 2.69 (ABq, 2H, J = 4.35 Hz), 2.41 (s, 3H). **^13^C NMR** (62.5 MHz, CDCl_3_): *δ* 144.9, 133.2, 132.6, 132.4, 132.3, 129.7 (2C), 128.2, 128.0, 127.8, 127.6, 127.5, 126.0, 125.6, 71.2, 57.1, 50.5, 37.6, 21.5. **HRMS (MALDI-FT ICR)** m/z [M+K]^+^ calculated for C_21_H_20_O_4_SK: 407.0719, found: 407.0713. **HPLC** (IC, 2-propanol/*n*-hexane = 10/90, rate = 1.0 mL/min, λ = 225 nm) t_R_ = 9.2 min (minor), 12.8 min (major).

**(*R*)-(2-(3,5-dimethylbenzyl)oxiran-2-yl)methyl 4-methylbenzenesulfonate (4j)**

Yellow wax (flash chromatography, eluent *n*-hexane/diethyl ether from 100/0 to 90/10), 15.5 mg, 45% yield. [α]_D_^20^ = - 5.8° (C = 0.76, CHCl_3_), 76% ee. **^1^H NMR** (300 MHz, CDCl_3_): *δ* 7.78 (d, 2H, J = 7.46 Hz), 7.34 (d, 2H, J = 7.46 Hz), 6.86 (s, 1H), 6.74 (s, 2H), 3.97 and 3.93 (ABq, 2H, J = 10.68 Hz), 2.93 and 2.80 (ABq, 2H, J = 15.03 Hz), 2.67 (A_2_, 2H), 2.45 (s, 3H), 2.26 (s, 6H). **^13^C NMR** (75 MHz, CDCl_3_): *δ* 144.9, 137.9, 134.9, 132.6, 129.7, 128.5, 127.9, 127.3, 71.2, 57.1, 50.4, 37.3, 21.5, 21.1. **HRMS (MALDI-FT ICR)** m/z [M+K]^+^ calculated for C_19_H_22_O_4_SK: 385.0876, found: 385.0870. **HPLC** (IC, 2-propanol/*n*-hexane = 10/90, rate = 1.0 mL/min, λ = 230 nm) t_R_ = 33.3 min (minor), 40.4 min (major).

**(*R*)-(2-(3,4,5-trifluorobenzyl)oxiran-2-yl)methyl 4-methylbenzenesulfonate (4k)**

Yellow wax (flash chromatography, eluent *n*-hexane/diethyl ether from 100/0 to 90/10), 17.0 mg, 45% yield. [α]_D_^21^ = - 14.1° (C = 0.35, CHCl_3_), 60% ee. **^1^H NMR** (600 MHz, CDCl_3_): *δ* 7.76 (d, 2H, J = 7.84 Hz), 7.35 (d, 2H, J = 7.84 Hz), 6.73 (dd, 2H, J = J = 7.12 Hz), 3.93 and 3.87 (AX, 2H, J = 9.65 Hz), 2.94 and 2.81 (AX, 2H, J = 15.44 Hz), 2.71 and 2.62 (AX, 2H, J = 3.86 Hz), 2.46 (s, 3H). **^13^C NMR** (150 MHz, CDCl_3_): *δ* 150.9 (ddd, ^1^J_C-F_ = 250.9 Hz, ^2^J_C-F_ = 9.6 Hz, J_C-F_ = 3.8 Hz), 145.5, 138.9 (dt, ^1^J_C-F_ = 250.7 Hz, ^2^J_C-F_ = 14.3 Hz), 132.3, 131.3 (dd, ^2^J_C-F_ = 13.3 Hz, ^3^J_C-F_ = 9.3 Hz), 130.0, 127.9, 113.7 (dd, ^2^J_C-F_ = 17.1 Hz, ^3^J_C-F_ = 4.2 Hz), 70.9, 56.4, 50.7, 36.7, 21.6. **^19^F NMR** (564 MHz, CDCl_3_): *δ* - 134.14 (d, J = 27.0 Hz), -162.47 (t, J = 20.64 Hz). **HRMS (MALDI-FT ICR)** m/z [M+K]^+^ calculated for C_17_H_15_F_3_O_4_SK: 411.0280, found: 411.0274. **HPLC** (OD-H, 2-propanol/*n*-hexane = 5/95, rate = 1.0 mL/min, λ = 235 nm) t_R_ = 20.9 min (minor), 22.5 min (major).

**(2-((1-(phenylsulfonyl)-1*H*-indol-3-yl)methyl)oxiran-2-yl)methyl 4-methylbenzenesulfonate (4l)**

Yellow wax (flash chromatography, eluent *n*-hexane/diethyl ether from 100/0 to 80/20), 36.7 mg, 74% yield. [α]^22^ = + 1.7° (C = 0.64, CHCl_3_), 47% ee. **^1^H NMR** (250 MHz, CDCl_3_): *δ* 7.95 (d, 1H, J = 9.03 Hz), 7.85 (d, 2H, J = 6.87 Hz), 7.72 (d, 2H, J = 7.60 Hz), 7.52-7.21 (m, 9H), 3.93 and 3.89 (ABq, 2H, J = 11.04 Hz), 3.06 and 2.97 (ABq, 2H, J = 15.52 Hz), 2.68 and 2.64 (ABq, 2H, J = 5.02 Hz), 2.43 (s, 3H). **^13^C NMR** (62.5 MHz, CDCl_3_): *δ* 145.2, 137.8, 134.9, 133.7, 132.2, 131.1, 130.6, 129.8,129.2, 127.8, 126.6, 124.8, 123.3, 119.8, 116.4, 113.5, 71.0, 56.1, 50.9, 27.1, 21.6. (**HRMS (MALDI-FT ICR)** m/z [M+K]^+^ calculated for C_25_H_23_NO_6_S_2_K: 536.0604, found: 536.0601. **HPLC** (IC, 2-propanol/*n*-hexane = 20/80, rate = 1.0 mL/min, λ = 220 nm) t_R_ = 61.9 min (minor), 70.9 min (major).

**(2-(pent-2-yn-1-yl)oxiran-2-yl)methyl 4-methylbenzenesulfonate (4m)**

Colourless oil (flash chromatography, eluent n-hexane/diethyl ether from 100/0 to 90/10), 10.5 mg, 35% yield. [α]_D_^22^ = + 1.1° (C = 0.51, CHCl_3_), 23% ee. **^1^H NMR** (300 MHz, CDCl_3_): *δ* 7.79 (d, 2H, J = 7.85 Hz), 7.34 (d, 2H, J = 7.34 Hz), 4.18 and 4.06 (AX, 2H, J = 10.02 Hz), 2.76 and 2.65 (AX, 2H, J = 5.84 Hz), 2.61 and 2.41 (AX, 2H, J = 17.50 Hz), 2.44 (s, 3H), 2.12-2.04 (m, 2H), 1.05 (t, 3H, J = 7.50 Hz). **^13^C NMR** (300 MHz, CDCl_3_): *δ* 144.9, 132.4, 129.8, 127.9, 84.9, 72.2, 70.8, 55.7, 50.4, 22.4, 21.5, 13.8, 12.2. **HRMS (MALDI-FT ICR)** m/z [M+K]^+^ calculated for C_15_H_18_O_4_SK: 333.0563, found: 333.0557. **HPLC** (AD-H, 2-propanol/*n*-hexane = 10/90, rate = 1.0 mL/min, λ = 230 nm) t_R_ = 9.9 min (minor), 10.7 min (major).

**(*R*)-(2-phenyloxiran-2-yl)methyl 4-methylbenzene sulfonate (4n)**

Data are consistent with those reported in the literature.^[[18]](#footnote-18)^ White wax (flash chromatography, eluent *n*-hexane/diethyl ether from 100/0 to 90/10), 15.9 mg, 45% yield. [α]^21^ = + 1.3° (C = 1.05, CHCl_3_), 37% ee. **^1^H NMR** (300 MHz, CDCl_3_): *δ* 7.72 (d, 2H, J = 8.61 Hz), 7.30 (s, 6H), 7.26 (s, 1H), 4.48 and 4.32 (AX, 2H, J = 12.34 Hz), 3.09 and 2.81 (AX, 2H, J = 5.37 Hz), 2.44 (s, 3H). **^13^C NMR** (62.5 MHz, CDCl_3_): *δ* 144.9, 135.6, 132.4, 129.7, 128.4, 128.2, 127.8, 125.9, 71.3, 57.5, 53.2, 21.5. **HRMS (MALDI-FT ICR)** m/z [M]^+^ calculated for C_16_H_16_O_4_S: 304.0769, found: 304.0764. **HPLC** (AS-H, 2-propanol/*n*-hexane = 10/90, rate = 1.0 mL/min, λ = 230 nm) t_R_ = 27.8 min (minor), 33.4 min (major).

## **Scale-up of the asymmetric synthesis of epoxide (*R*)-4a**

In a round-bottom flask, sodium bicarbonate (84.2 mg, 1 mmol), **6a** (50.7 mg, 0.2 mmol), **9a** (93.3 mg, 0.2 mmol) were added to a solution of 2-benzyl-2-hydroxypropane-1,3-diyl bis(4-methylbenzenesulfonate) **3a** (490.6 mg, 1 mmol) in toluene (0.5 mL). The reaction mixture was stirred at room temperature for five days (monitored by TLC, eluent: *n*-hexane/ethyl acetate 8/2, visualized by UV light and phosphomolybdic acid solution). After completion, the crude product was purified by flash chromatography (eluent: *n*-hexane/diethyl ether, gradient from 100:0 to 90:10) to afford product **4a** in 37% yield (117.8 mg) and 60% ee. Catalyst **6a** (90% yield) and catalyst **9a** (95% yield) were recovered at the end of the reaction by column chromatography and found to be pure by^1^H NMR analysis.

# Ring opening reaction of racemic epoxide 4a by 4-bromobenzenesulfonate

In a round-bottom flask, 4-bromobenzenesulfonic acid (25.5 mg, 0.1 mmol) was added to the reaction mixture containing racemic (2-benzyloxiran-2-yl)methyl 4-methylbenzensulfonate **4a** (0.1 mmol), the catalytic couple **6a** (5.07 mg, 0.02 mmol)/**9a** (9.33 mg, 0.02 mmol), a slight excess of NaHCO_3_ (16.8 mg, 0.2 mmol), and molecular sieves (3Å) in toluene (0.5 mL). The reaction was stirred at room temperature for four days (checked by TLC, *n*-hexane/ethyl acetate 8:2 as eluent, revealed by UV light and phosphomolybdic acid solution). The crude reaction mixture was purified by flash chromatography using *n*-hexane/ethyl acetate as eluent (gradient from 100:0 to 60:40), to isolate compound **3a’** in 54% yield. The analogous reaction carried out with *p*-toluensulfonic acid afforded compound **3a** in 45% yield. Unreacted **4a** was recovered and found racemic after chiral HPLC analysis.

**2-benzyl-2-hydroxy-3-(tosyloxy)propyl 4-bromobenzenesulfonate (3a’)**

White wax (flash chromatography, eluent *n*-hexane/ethyl acetate from 100/0 to 60/40), 29.9 mg, 54% yield. **^1^H NMR** (600 MHz, CDCl_3_): *δ* 7.78-7.73 (m, 6H), 7.38 (d, 2H, J = 8.35 Hz), 7.26-7.24 (m, 3H), 7.09 (d, 2H, J = 6.56 Hz), 3.94-3.86 (m, 4H), 2.80 (A_2_, 2H), 2.50 (s, 3H). **^13^C NMR** (150 MHz, CDCl_3_): *δ* 145.4, 134.2, 133.5, 132.8, 132.0, 130.4, 130.1, 129.5, 129.4, 128.6, 128.0, 127.4, 71.8, 70.5, 70.1, 39.4, 21.7. **HRMS (MALDI-FT ICR)** m/z [M+K]^+^ calculated for C_23_H_23_BrO_7_S_2_K: 592.9706, found: 592.9702.

# Elaboration of the enantioenriched epoxide 4a

## **Procedure for the synthesis of (*R*)-2-((2-benzyloxiran-2-yl)methoxy)isoindole-1,3-dione 14**

*N*-Hydroxyphthalimide (48.9 mg, 0.3 mmol) and triethylamine (0.062 mL, 0.45 mmol) were added to a solution of (*R*)-(2-benzyloxiran-2-yl)methyl 4-methylbenzensulfonate **4a** (0.15 mmol) in 1,4-dioxane (0.1 mL). The solution was stirred for five days at 60 °C (monitored by TLC, *n*-hexane/ethyl acetate 8/2, UV light and phosphomolybdic acid solution). After completion, the crude product was purified by flash chromatography (eluent: *n*-hexane/diethyl ether from 100/0 to 85/15) to afford product **14**.

**(*R*)-2-((2-benzyloxiran-2-yl)methoxy)isoindole-1,3-dione (14)**

White wax (flash chromatography, eluent *n*-hexane/diethyl ether from 100/0 to 85/15), 37.5 mg, 80% yield. [α]_D_^25^ = - 23.9° (C = 0.85, CHCl_3_), 34% ee. **^1^H NMR** (400 MHz, CDCl_3_): *δ* 7.91 (m, 2H), 7.82 (m, 2H), 7.40-7.31 (m, 5H), 4.22 (A_2_, 2H), 3.49 and 3.23 (AX, 2H, J = 14.93 Hz), 2.77 and 2.73 (ABq, 2H, J = 4.00 Hz). **^13^C NMR** (100 MHz, CDCl_3_): *δ* 163.3, 135.9, 134.6, 130.1, 128.9, 128.5, 126.9, 123.7, 79.6, 57.6, 49.9, 37.5. **HRMS (MALDI-FT ICR)** m/z [M+K]^+^ calculated for C_18_H_15_NO_4_K: 348.0638, found: 348.0633. **HPLC** (OD-H, 2-propanol/*n*-hexane = 10/90, rate = 1.0 mL/min, λ = 230 nm) t_R_ = 16.5 min (minor), 18.2 min (major).

## **Procedure for the synthesis of (*S*)-2-benzyl-2-hydroxy-3-(phenylthiol)propyl 4-methylbenzenesulfonate 15**

In a round-bottom flask under nitrogen, thiophenol (0.024 mL, 0.24 mmol) and triethylamine (0.004 mL, 0.03 mmol) were added to a solution of (*R*)-(2-benzyloxiran-2-yl)methyl 4-methylbenzensulfonate **4a** (0.15 mmol) in toluene (1.5 mL). The reaction was stirred at 60 °C for 48 hours (monitored by TLC, eluent: *n*-hexane/ethyl acetate 8/2, visualized by UV light and phosphomolybdic acid solution). After completion, the crude product was purified by flash chromatography (eluent: *n*-hexane/diethyl ether from 100/0 to 75/25) to afford product **15**.

**(*S*)-2-benzyl-2-hydroxy-3-(phenylthiol)propyl 4-methylbenzenesulfonate (15)**

White wax (flash chromatography, eluent *n*-hexane/diethyl ether from 100/0 to 75/25), 56.0 mg, 87% yield. [α]_D_^24^ = + 2.6° (C = 0.84, CHCl_3_), 54% ee. **^1^H NMR** (400 MHz, CDCl_3_): *δ* 7.81 (d, 3H, J = 9.00 Hz), 7.40-7.23 (m, 11H), 3.96 and 3.92 (ABq, 2H, J = 10.39 Hz), 3.18 and 3.12 (ABq, 2H, J = 12.53 Hz), 2.97 (A_2_, 2H), 2.52 (s, 3H), 2.45 (bs, 1H). **^13^C NMR** (100 MHz, CDCl_3_): *δ* 145.1, 135.9, 134.9, 132.4, 130.6, 129.9, 129.7, 129.2, 128.5, 128.1, 127.1, 126.7, 73.2, 71.9, 42.0, 41.4, 21.7. **HRMS (MALDI-FT ICR)** m/z [M+K]^+^ calculated for C_23_H_24_O_4_S_2_K: 467.0753, found: 467.0772. **HPLC** (IA-3, 2-propanol/*n*-hexane = 10/90, rate = 1.0 mL/min, λ = 230 nm) t_R_ = 28.2 min (minor), 35.7 min (major).

## **Procedure for the synthesis of 3-benzyl-1,2,3,4,5,6-hexahydrobenzo[*b*][1,5]diazocin-3-ol 16**

In a round-bottom flask under nitrogen, 2-amminomethylaniline (28.1 mg, 0.23 mmol) and triethylamine (0.021 mL, 0.15 mmol) were added to a solution of (*R*)-(2-benzyloxiran-2-yl)methyl 4-methylbenzensulfonate **4a** (0.15 mmol) in ethanol (0.75 mL). The reaction was stirred at 60 °C for 42 hours (monitored by TLC, eluent: *n*-hexane/ethyl acetate 6/4, visualized by UV light and ninhydrin solution). After completion, the crude product was purified by flash chromatography (eluent: *n*-hexane/ethyl acetate from 100/0 to 40/60) to afford product **16**. The product could not be separated by extensive chiral HPLC analysis.

**3-benzyl-1,2,3,4,5,6-hexahydrobenzo[*b*][1,5]diazocine-3-ol (16)**

Colourless oil (flash chromatography, eluent *n*-hexane/ethyl acetate from 100/0 to 60/40), 26.8 mg, 66% yield. [α]^25^ = - 1.6° (C = 0.89, CHCl_3_), ee not determined. **^1^H NMR** (300 MHz, CDCl_3_): *δ* 7.38-7.19 (m, 5H), 7.12-7.07 (m, 2H), 7.00-6.98 (m, 1H), 6.68-6.62 (m, 2H), 3.62 (s, 2H), 3.40 (dd, 2H, J = 6.20 Hz, J = 1.67 Hz), 3.04 (s, 2H), 2.94 (d, 2H, J = 8.39 Hz). **^13^C NMR** (75 MHz, CDCl_3_): *δ* 146.6, 136.6, 129.9, 129.7, 128.4, 128.3, 126.7, 122.1, 117.5, 115.3, 70.4, 65.6 (2C), 62.4, 44.6. **HRMS (MALDI-FT ICR)** m/z [M+H]^+^ calculated for C_17_H_21_N_2_O: 269.1654, found: 269.1656.

## **Procedure for the synthesis of 1,3-dibenzylazetidin-3-ol 17**

In an ACE. tube, under nitrogen, benzylamine (0.016 mL, 0.15 mmol) and triethylamine (0.021 mL, 0.15 mmol) were added to a solution of (2-benzyloxiran-2-yl)methyl 4-methylbenzenesulfonate **4a** (0.1 mmol) in toluene (1 mL). The reaction was refluxed for 60 hours (monitored by TLC, eluent: *n*-hexane/ethyl acetate 6/4, visualized by UV light and ninhydrin solution). After completion, the crude product was purified by flash chromatography (eluent: *n*-hexane/ethyl acetate from 100/0 to 50/50) to afford product **17**.

**1,3-dibenzylazetidin-3-ol (17)**

Colourless oil (flash chromatography, eluent *n*-hexane/ethyl acetate from 100/0 to 60/40), 12.6 mg, 50% yield. **^1^H NMR** (400 MHz, CDCl_3_): *δ* 7.29-7.17 (m, 10H), 3.73 and 3.71 (ABq, 2H, J = 12.95 Hz), 3.10 and 2.83 (AX, 2H, J = 14.38 Hz), 2.79 and 2.65 (AX, 2H, J = 13.46 Hz), 2.77 (s, 1H), 2.58 (d, 1H, J = 4.99 Hz). **^13^C NMR** (62.5 MHz, CDCl_3_): *δ* 140.1, 136.7, 129.5, 128.2 (2C), 127.9, 126.8, 126.5, 59.5, 53.7, 51.4, 50.5, 39.3. **HRMS (MALDI-FT ICR)** m/z [M+H]^+^ calculated for C_17_H_20_NO: 254.1545, found: 254.1539.

# Derivatization of 3-benzyl-1,2,3,4,5,6-hexahydrobenzo[*b*][1,5]diazocine-3-ol 16

In a two neck flask, under nitrogen, containing 3-benzyl-1,2,3,4,5,6-hexahydrobenzo[*b*][1,5]diazocine-3-ol **16** (0.069 mmol) in dichloromethane (0.14 mL), triethylamine (0.015 mL, 0.103 mmol) was added. The solution was cooled at 0 °C and a solution of (Boc)_2_O (19.1 mg, 0.083 mmol) in dichloromethane (0.034 mL) was added dropwise. The reaction was stirred at room temperature for 16 hours (monitored by TLC, eluent: *n*-hexane/ethyl acetate 9/1, visualized by UV light and phosphomolybdic acid solution). After completion, the mixture was quenched with an aqueous solution of NH_4_Cl (10 mL). The aqueous phase was extracted with dichloromethane (2 x 10 mL). The organic phases combined were dried over Na_2_SO_4_ and concentrated under vacuum. The crude product was purified by flash chromatography (eluent: *n*-hexane/ethyl acetate from 100/0 to 80/20) to afford product **18**. The product could not be separated by extensive chiral HPLC analysis.

**di-*tert*-butyl 3-benzyl-3-hydroxy-3,4-dihydrobenzo[*b*][1,5]diazocine-1,5(*2H*, *6H*)-dicarboxilate (18)**

White wax (flash chromatography, eluent *n*-hexane/ethyl acetate from 100/0 to 80/20), 10.0 mg, 30% yield. **^1^H NMR** (400 MHz, CDCl_3_): *δ* 7.30-7.24 (m, 5H), 7.16 (d, 2H, J = 8.85 Hz), 7.02 (d, 1H, J = 7.78 Hz), 6.89 (t, 1H, J = 6.82 Hz), 3.71 (s, 2H), 3.56 (d, 2H, J = 8.60 Hz), 3.38 (s, 2H), 3.08 (d, 2H, J = 9.14 Hz), 1.55 (s, 9H), 1.50 (s, 9H). **^13^C NMR** (62.5 MHz, CDCl_3_): *δ* 153.1, 151.3, 138.9, 135.9, 129.8, 129.4, 128.4, 128.2, 126.7, 123.7, 121.7, 118.7, 82.4,79.6, 75.9, 63.0 (2C), 61.8, 40.3, 28.3(3C), 27.6 (3C).

# ^1^H NMR shifts for catalytic system activation


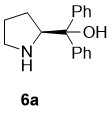

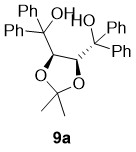

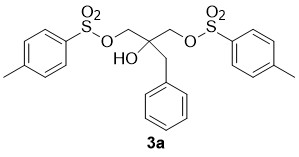

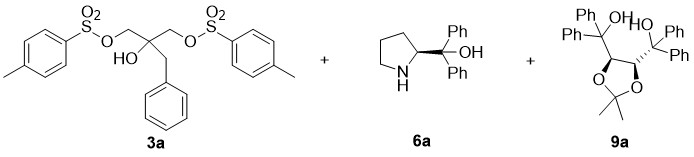

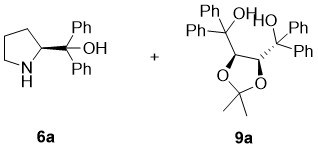

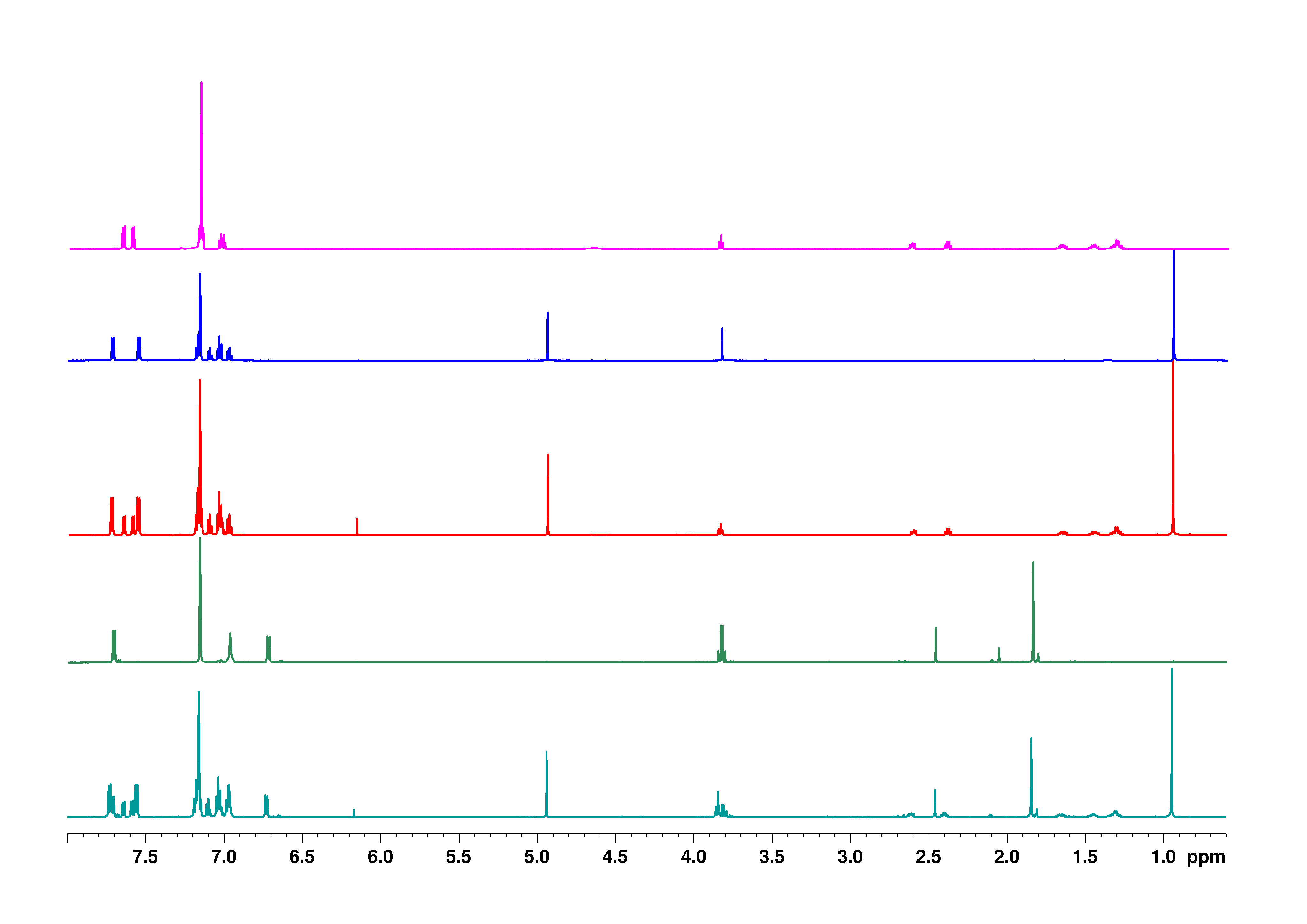


**E**

**D**

**C**

**A**

**B**

**Figure S1.** ^1^H-NMR spectra in C_6_D_6_ at 600 MHz of A) Catalyst **6a**; B) Catalyst **9a**; C) 1/1 mixture of **6a** and **9a**; D) **3a**; E) 1/1/1 mixture of **6a**, **9a** and **3a.**

# NMR Spectra

^1^H NMR in CDCl3 (300 MHz)


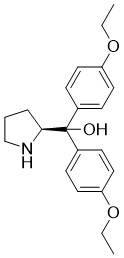

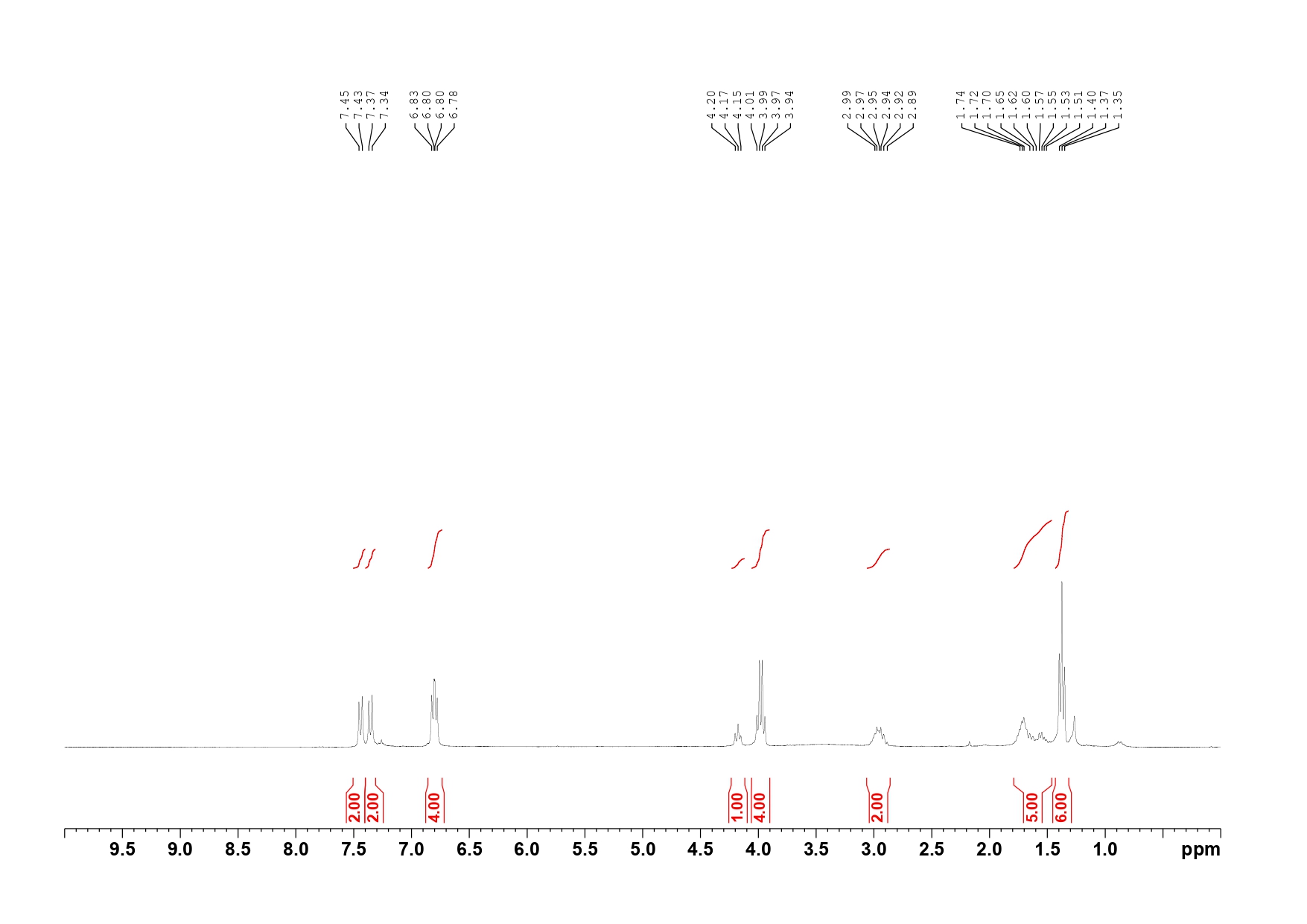


**6d**

^13^C NMR in CDCl3 (62.5 MHz)


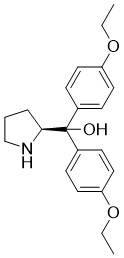

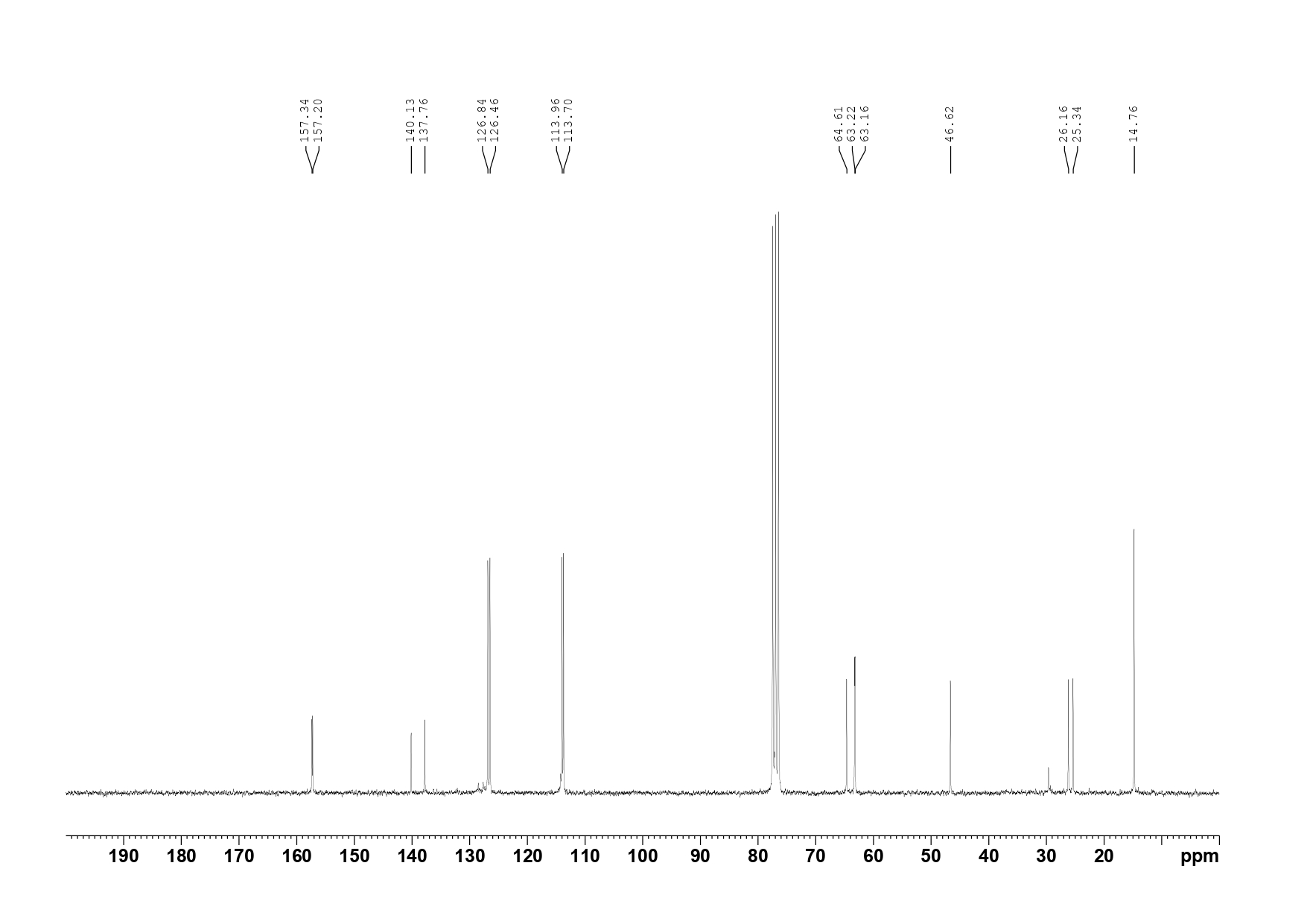


**6d**

^1^H NMR in CDCl_3_ (400 MHz)

_
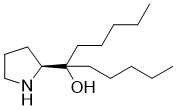
_

^
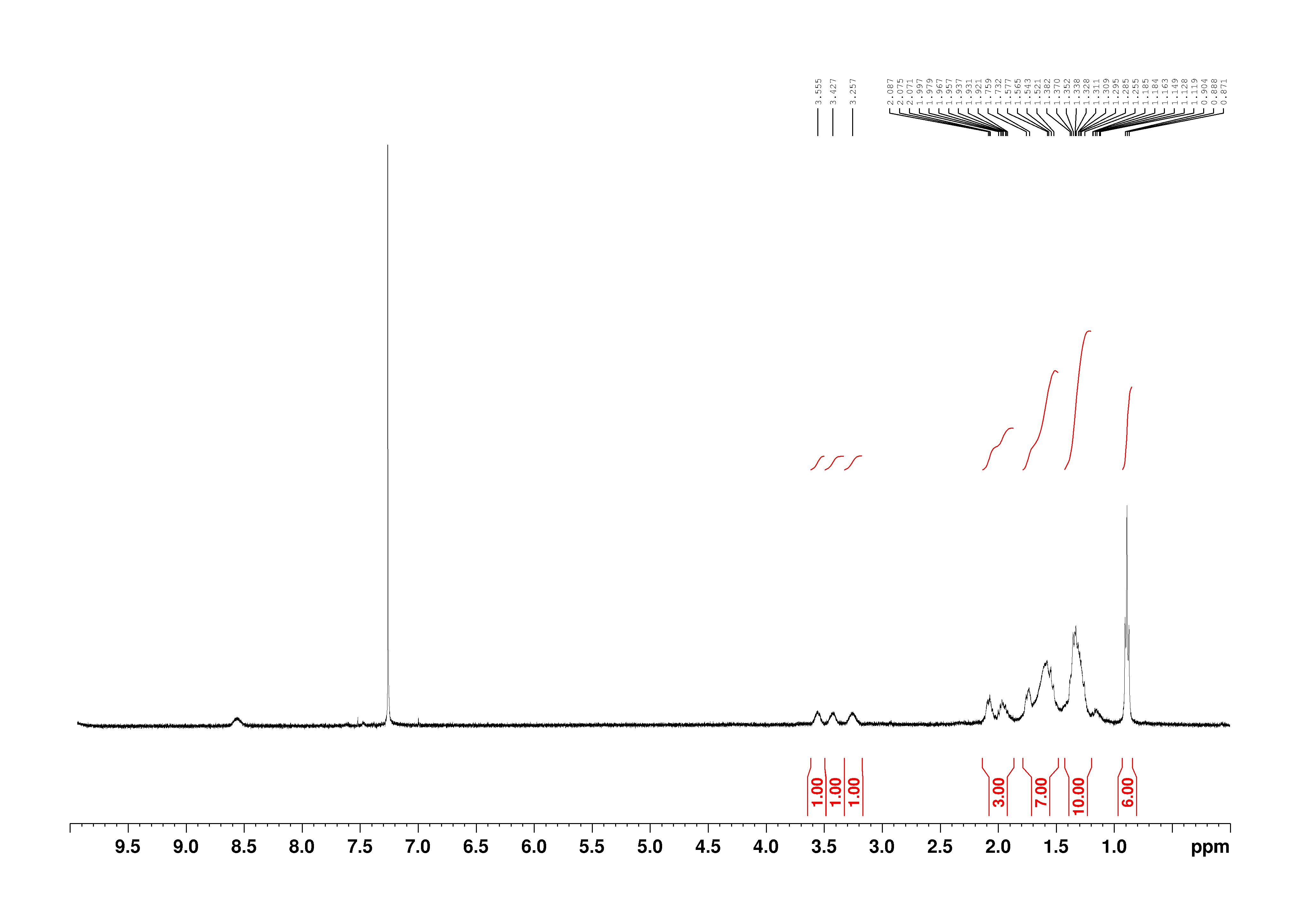
^

**6e**

^13^C NMR in CDCl_3_ (62.5 MHz)

_
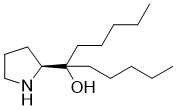
_

^
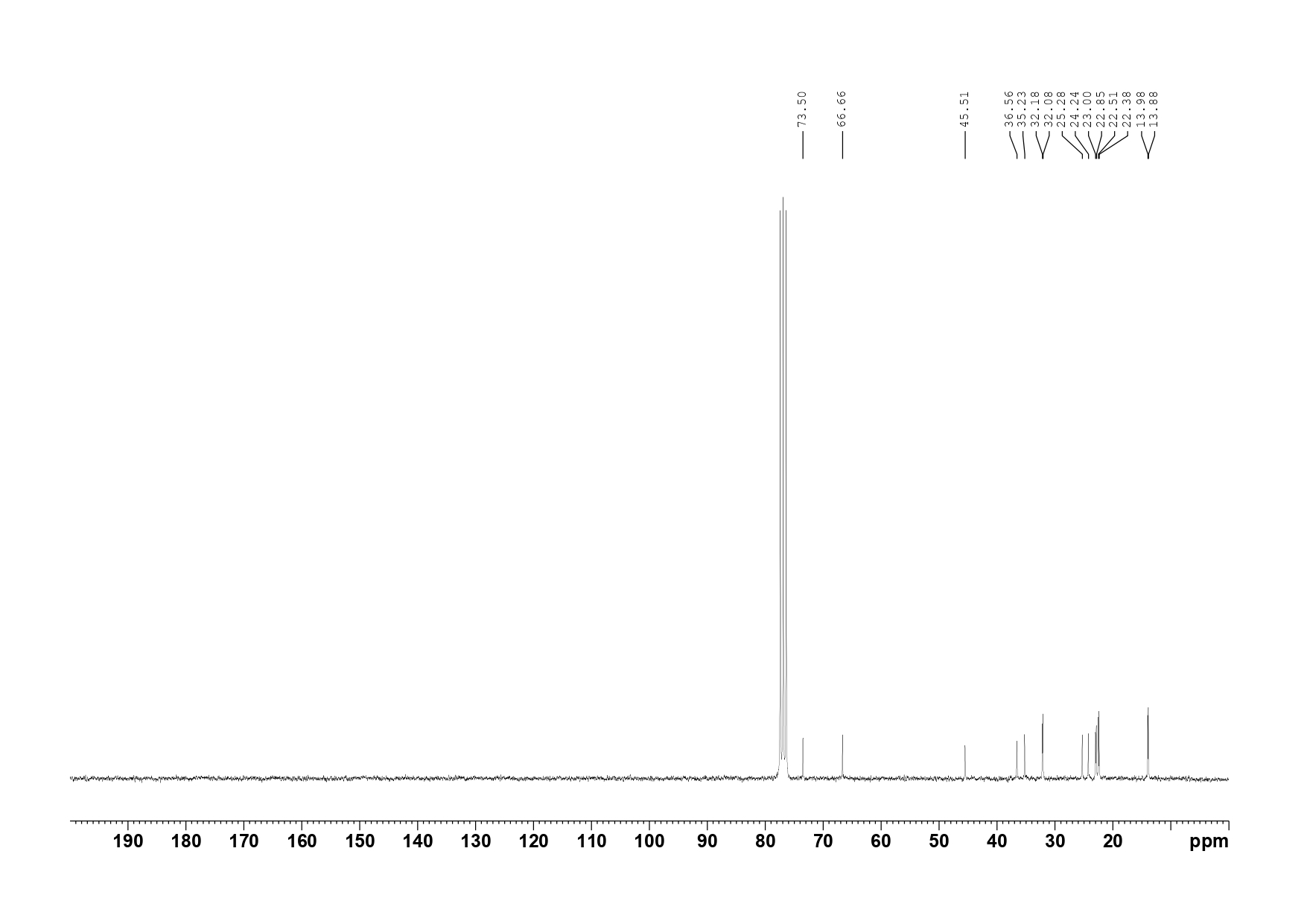
^

**6e**

^1^H NMR in CDCl_3_ (300 MHz)


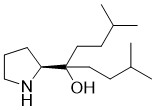


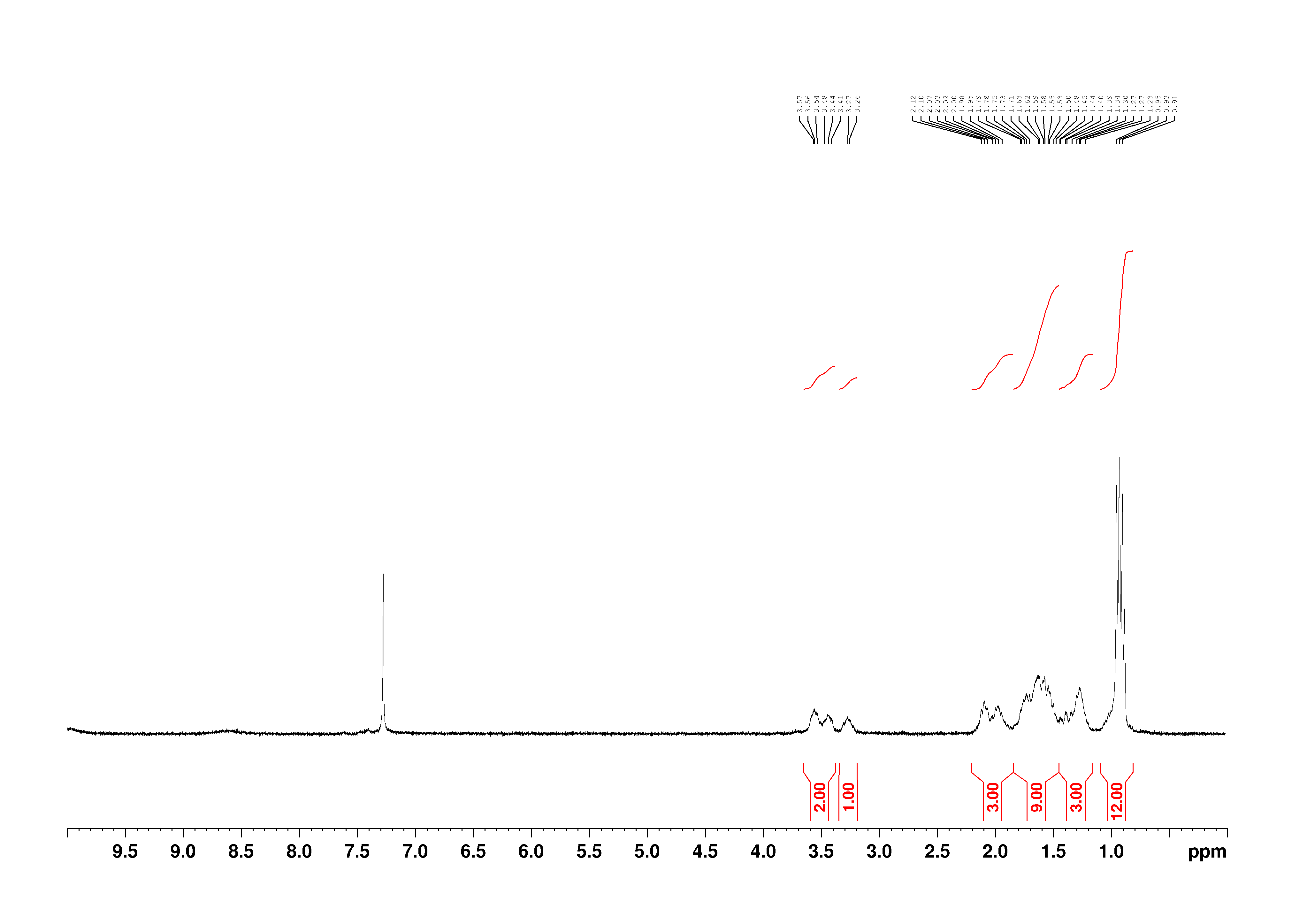


**6f**

^13^C NMR in CDCl_3_ (62.5 MHz)


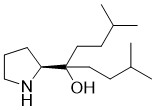


^
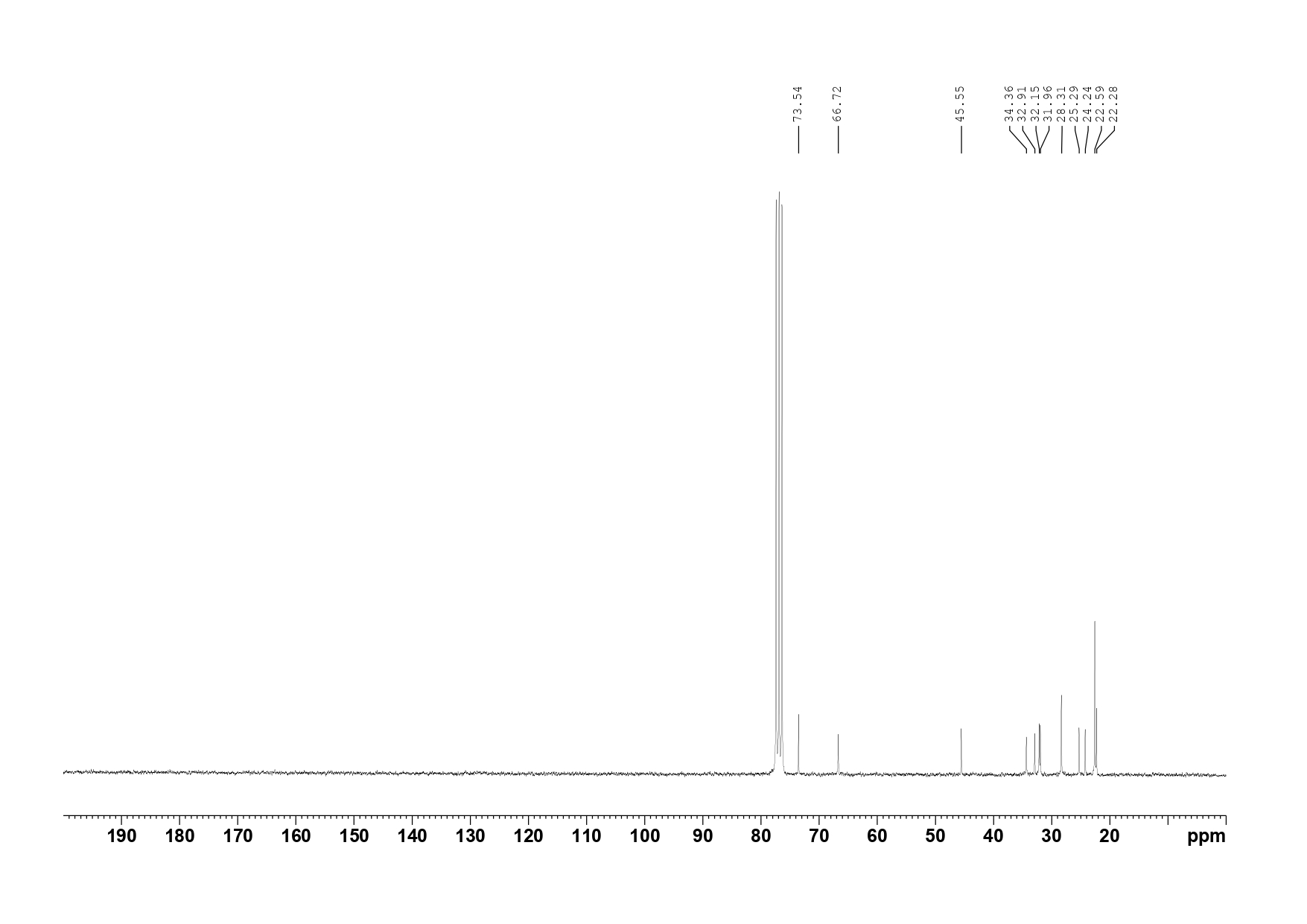
^

**6f**

^1^H NMR in CDCl_3_ (300 MHz)


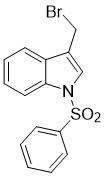
^
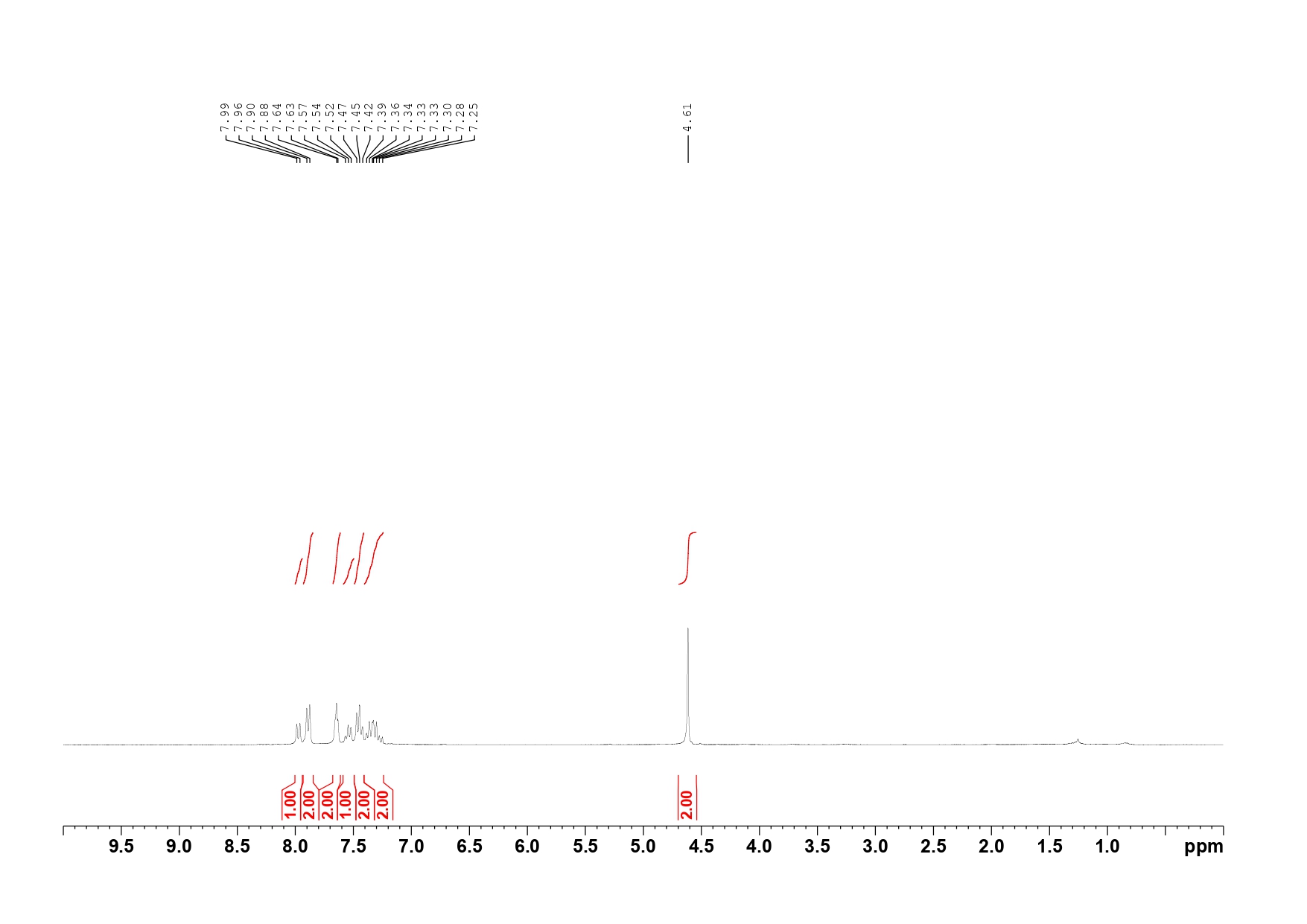
^

**U1**

^13^C NMR in CDCl_3_ (75 MHz)


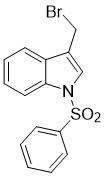
^
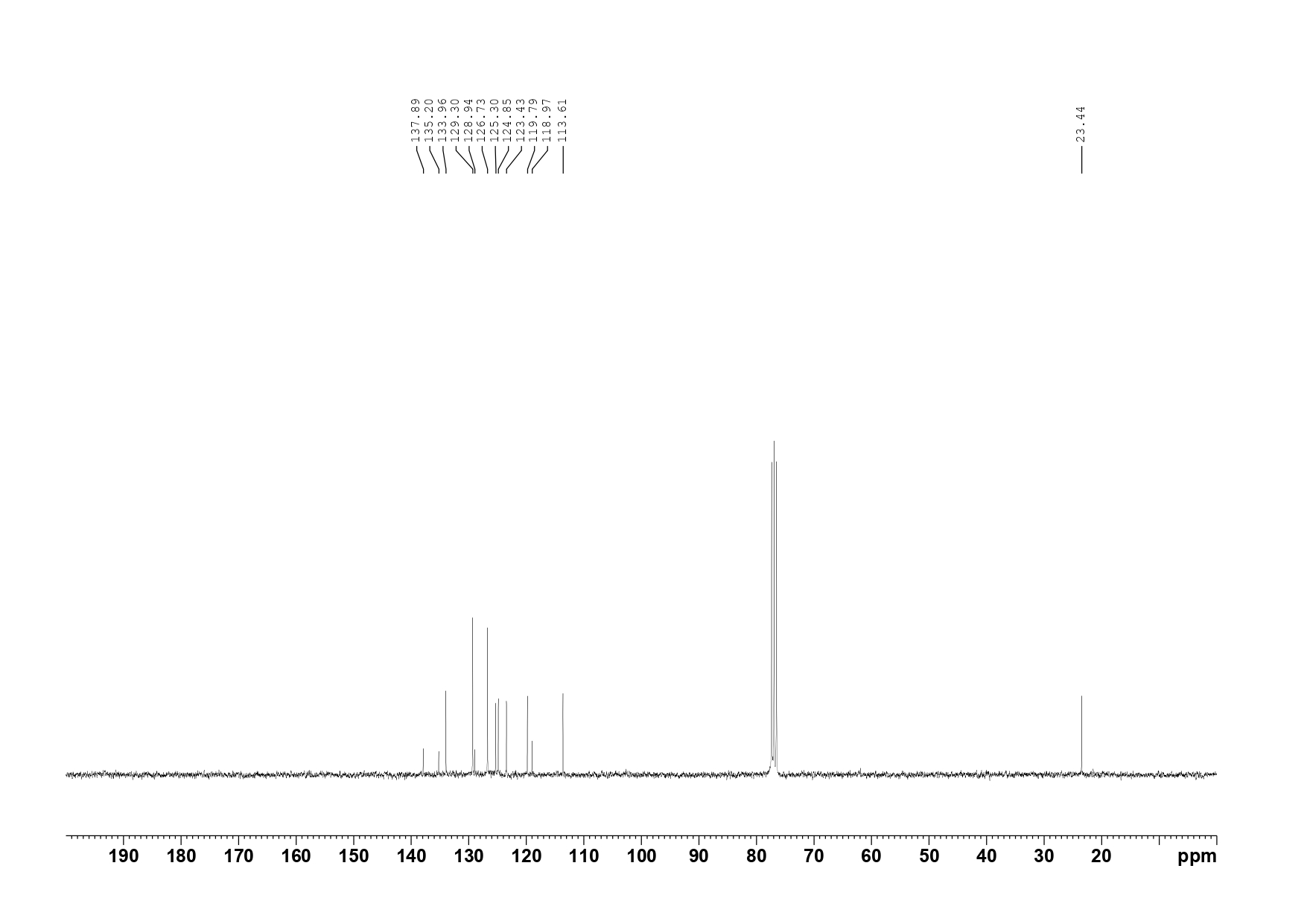
^

**U1**

^1^H NMR in CDCl_3_ (400 MHz)


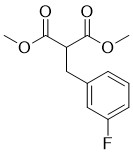

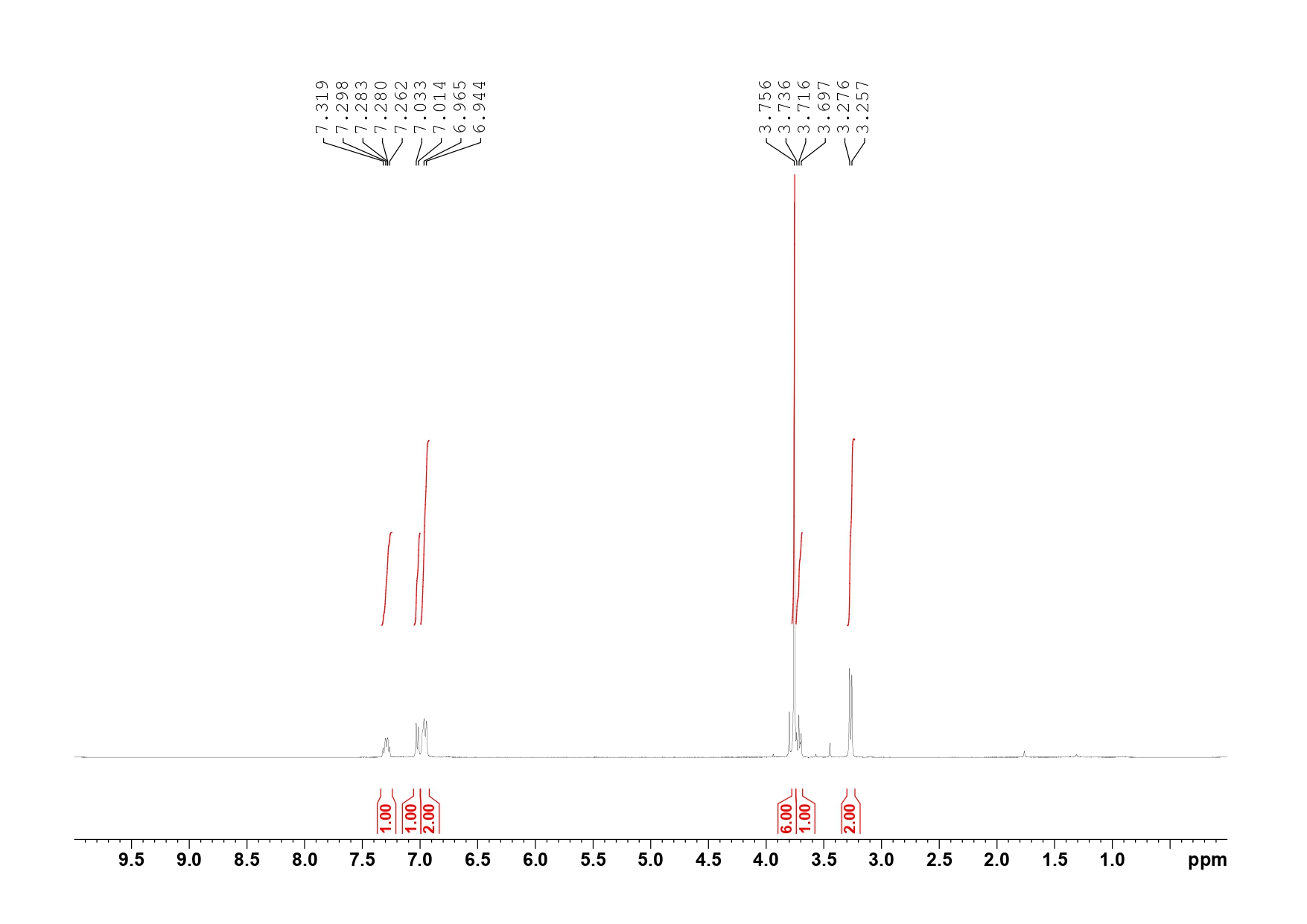


**1e**

**4d**

^13^C NMR in CDCl_3_ (100 MHz)


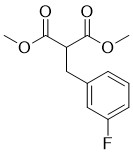
^
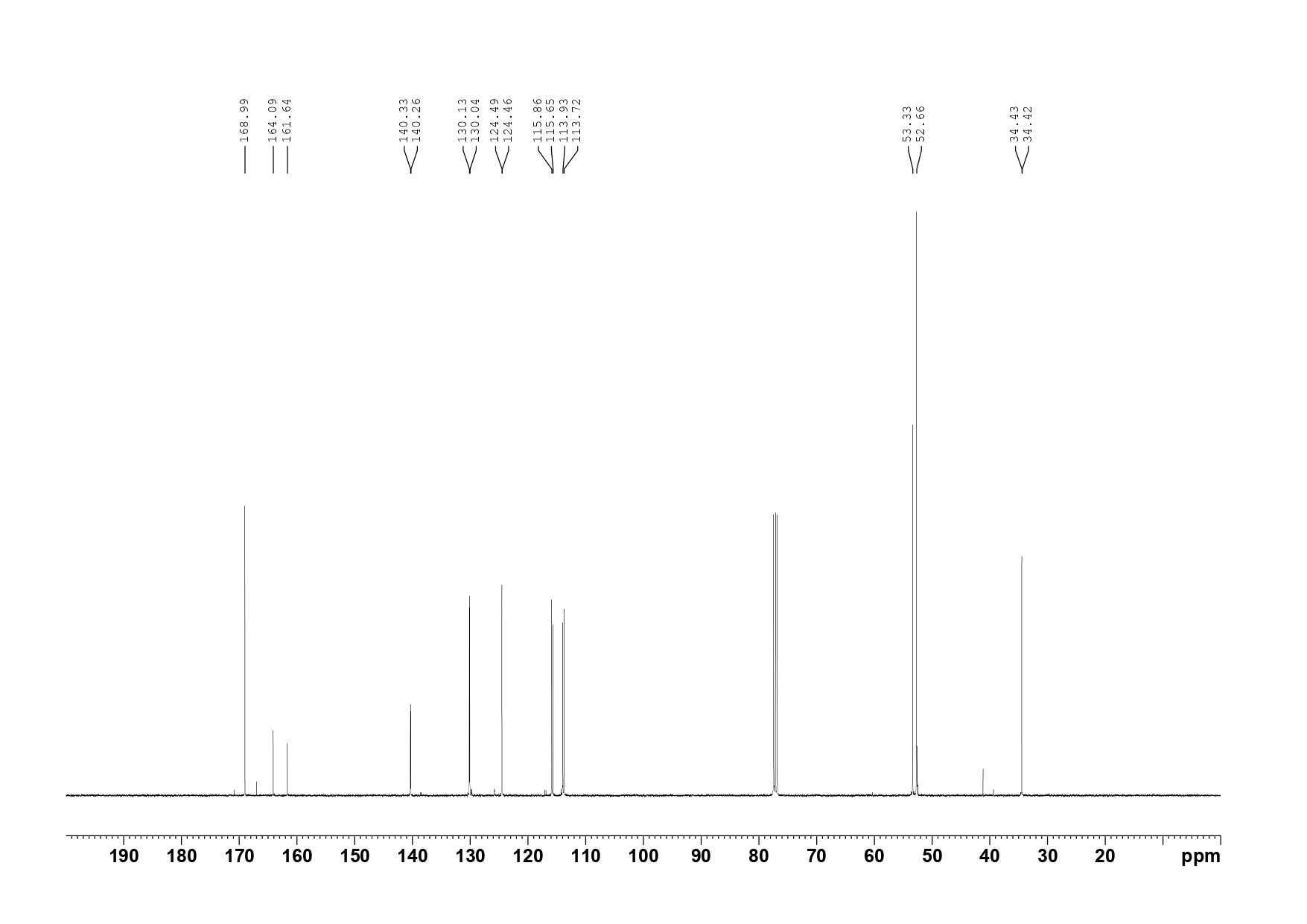
^

**1e**

^19^F NMR in CDCl_3_ (376 MHz)


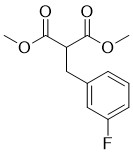


^
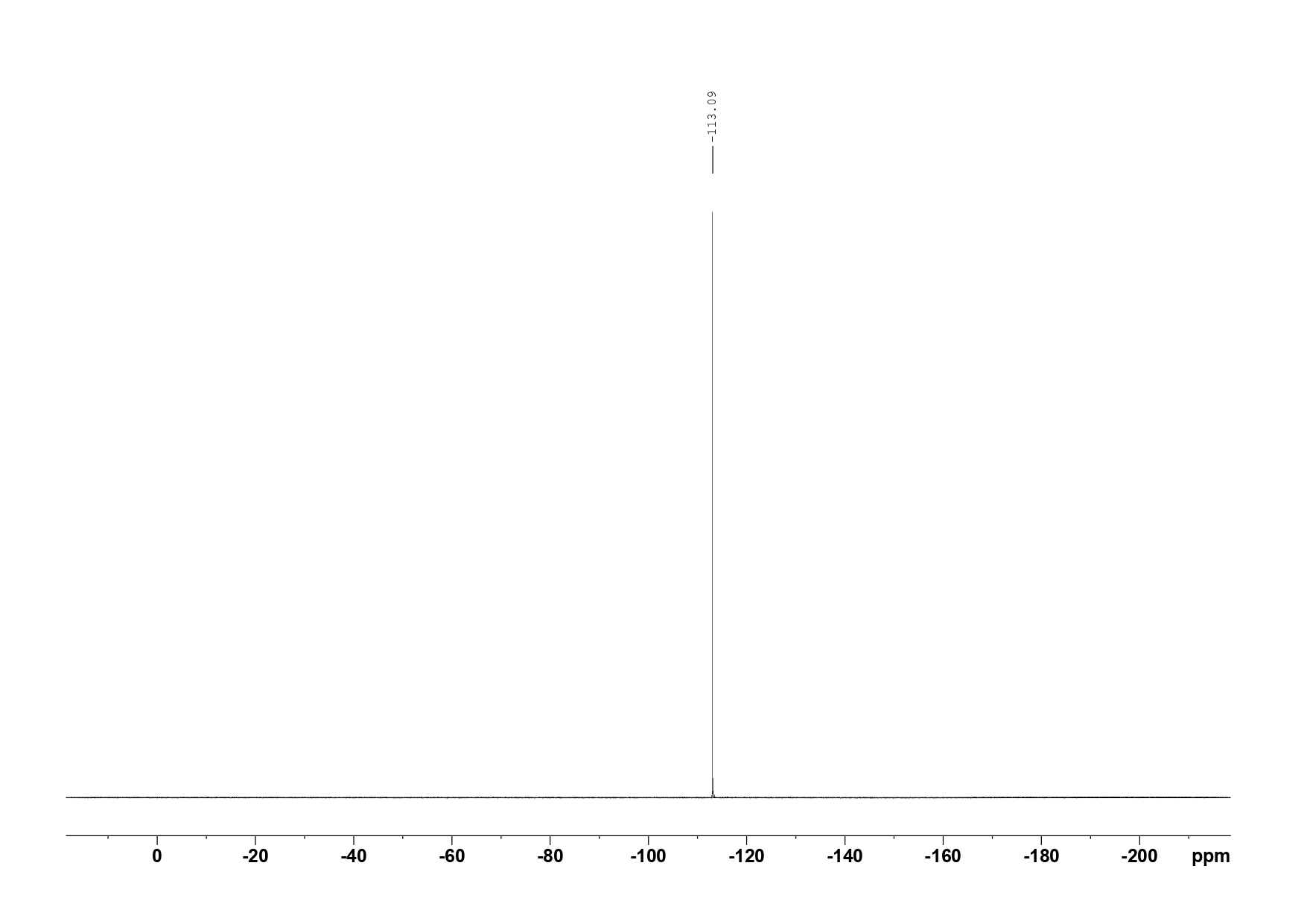
^

**1e**

^1^H NMR in CDCl_3_ (400 MHz)


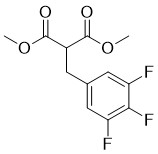
^
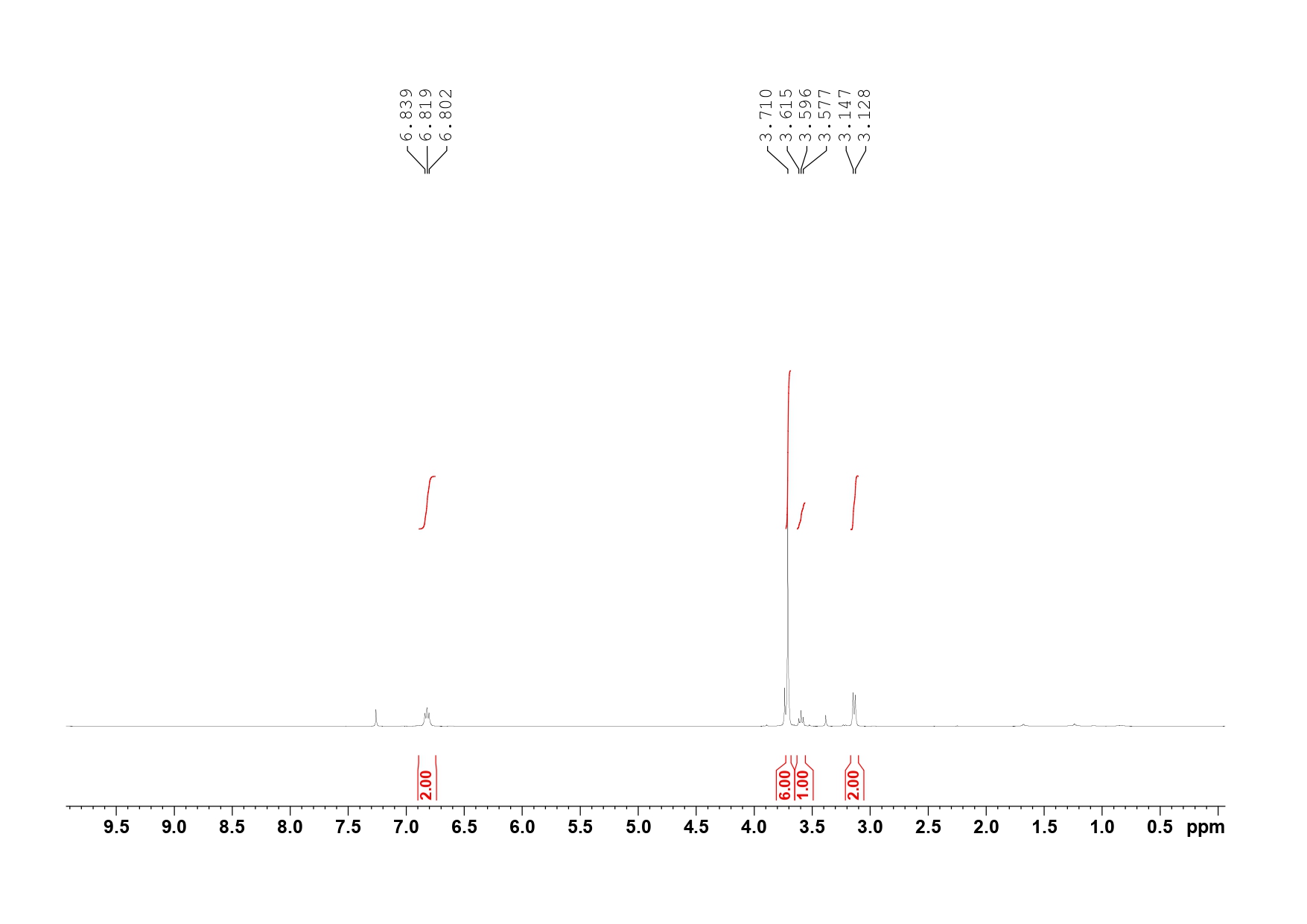
^

**1k**

^13^C NMR in CDCl_3_ (100 MHz)


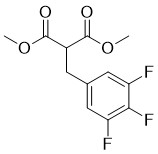
^
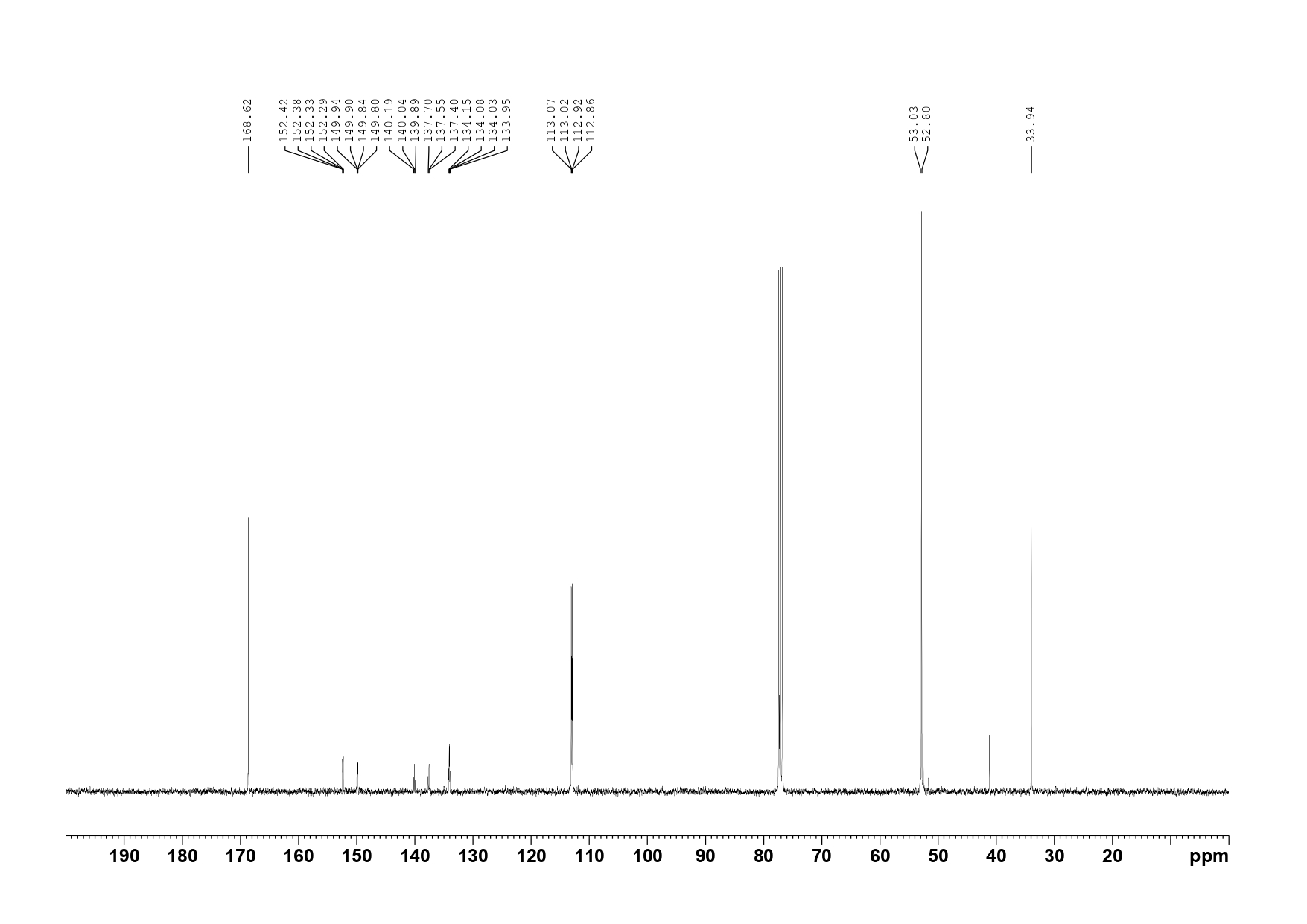
^

**1k**

^19^F NMR in CDCl_3_ (376 MHz)


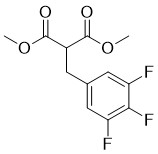
^
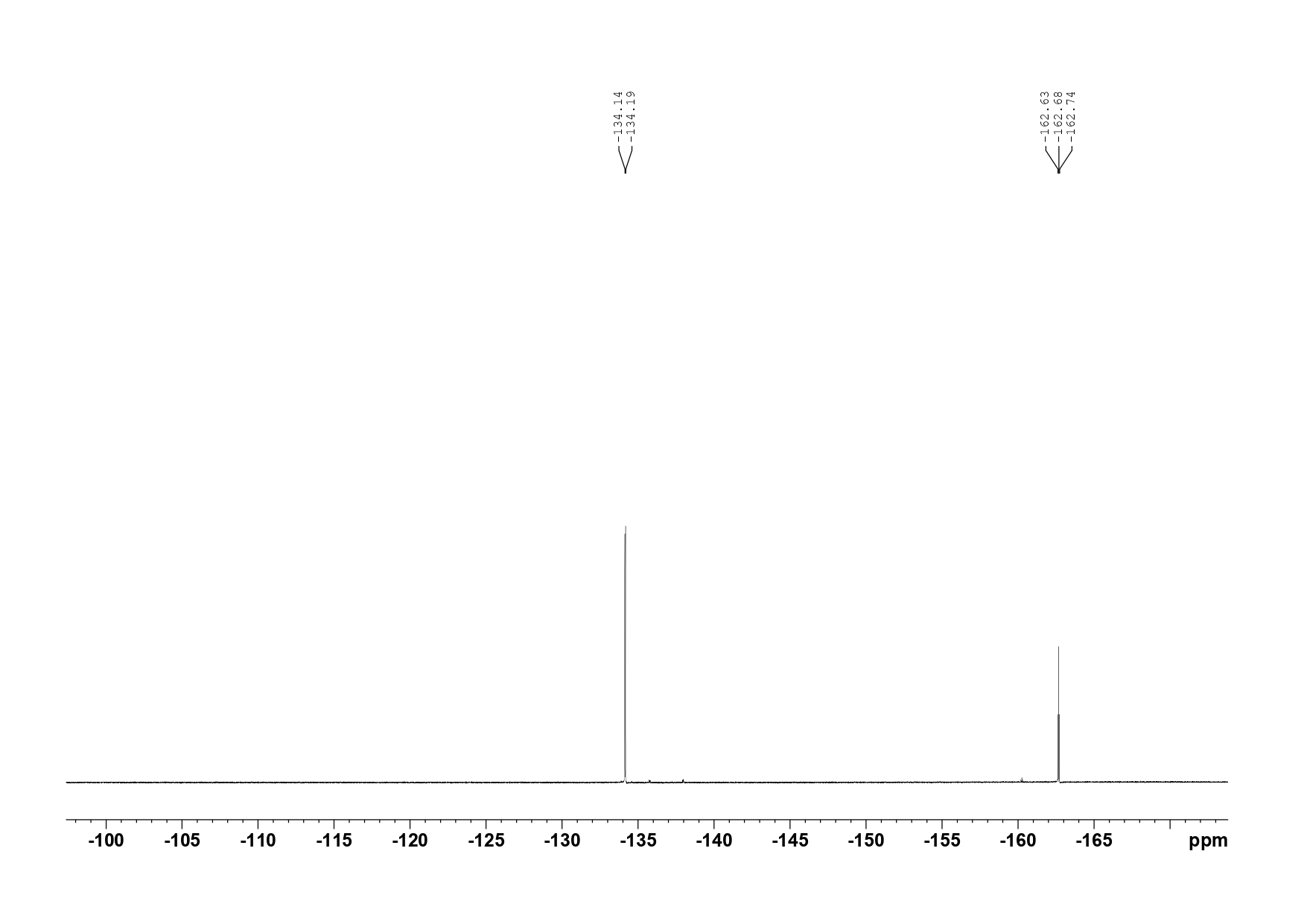
^

**1k**

^1^H NMR in CDCl_3_ (300 MHz)


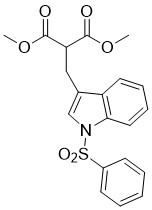


^
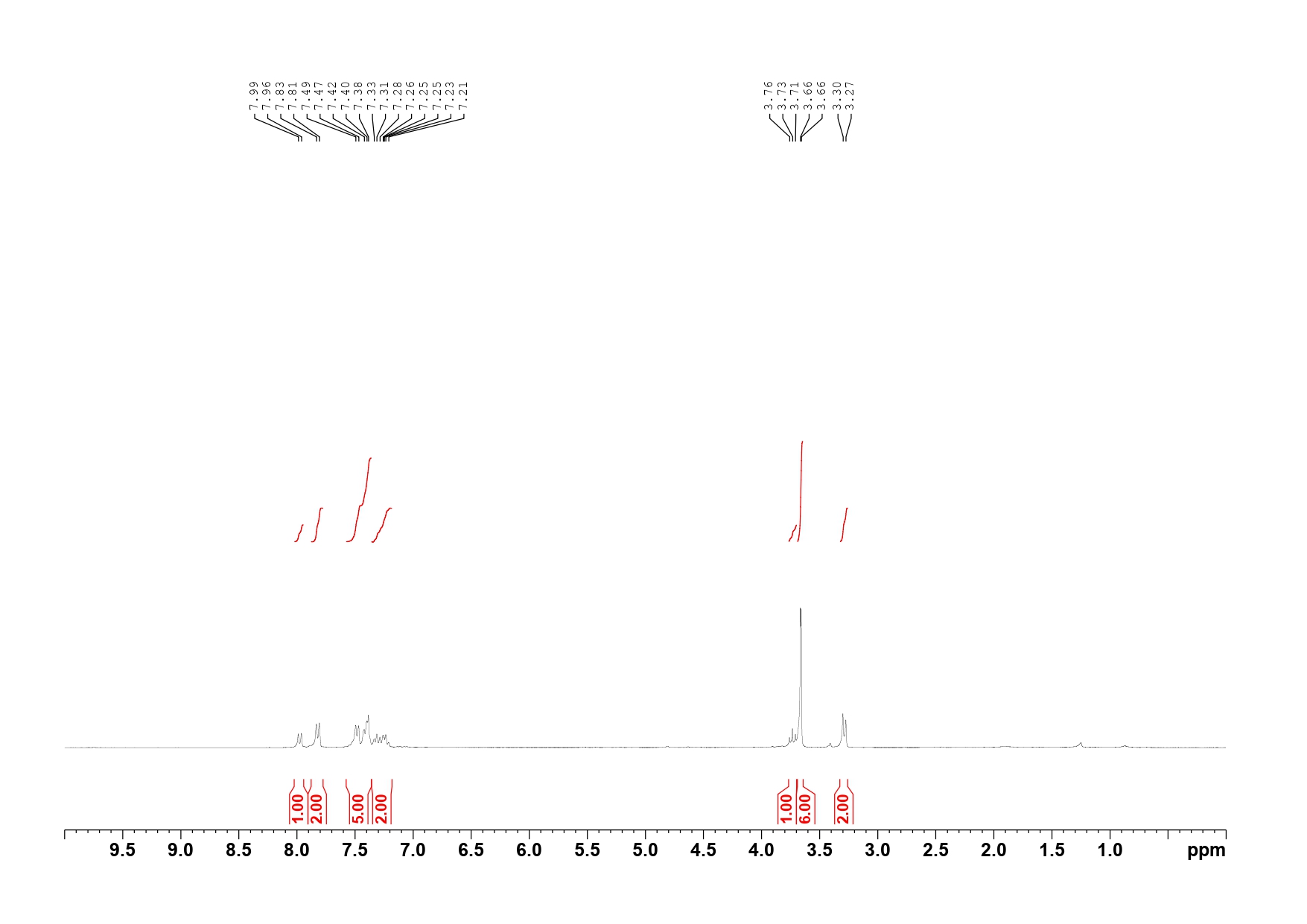
^

**1l**

^13^C NMR in CDCl_3_ (75 MHz)


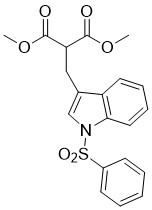
^
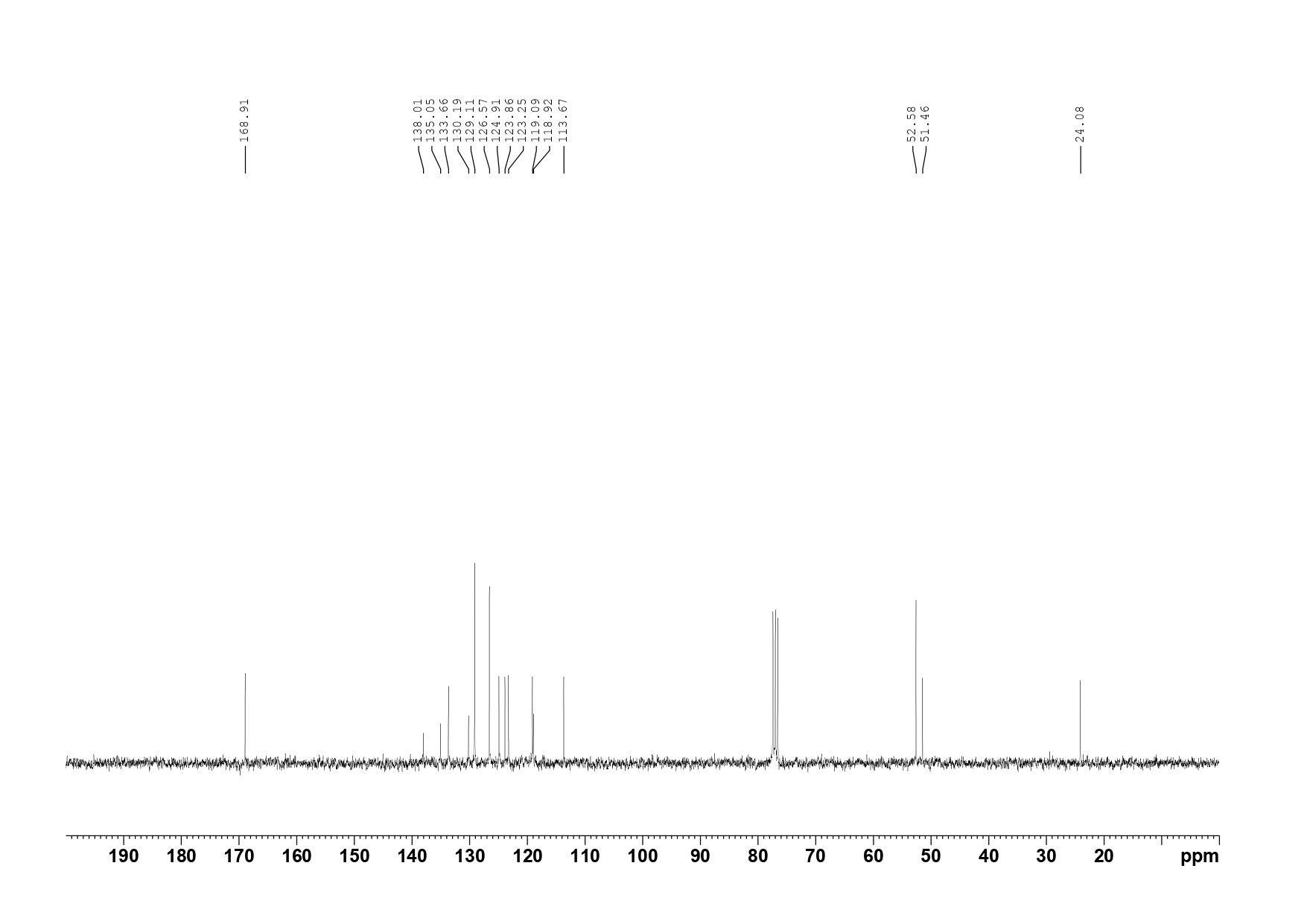
^

**1l**

^1^H NMR in CDCl_3_ (300 MHz)


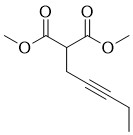
^
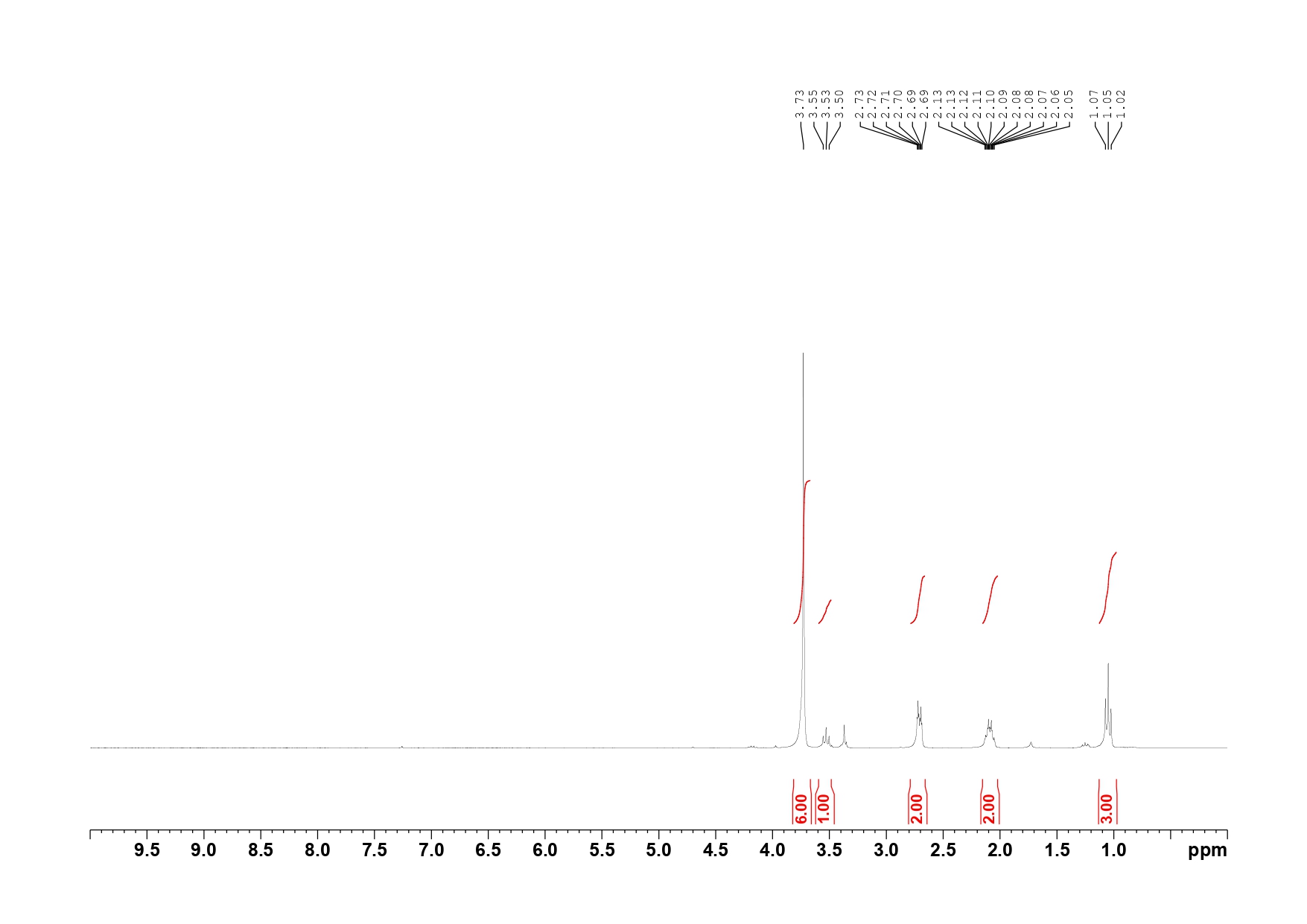
^

**1m**

^13^C NMR in CDCl_3_ (75 MHz)


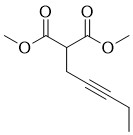


^
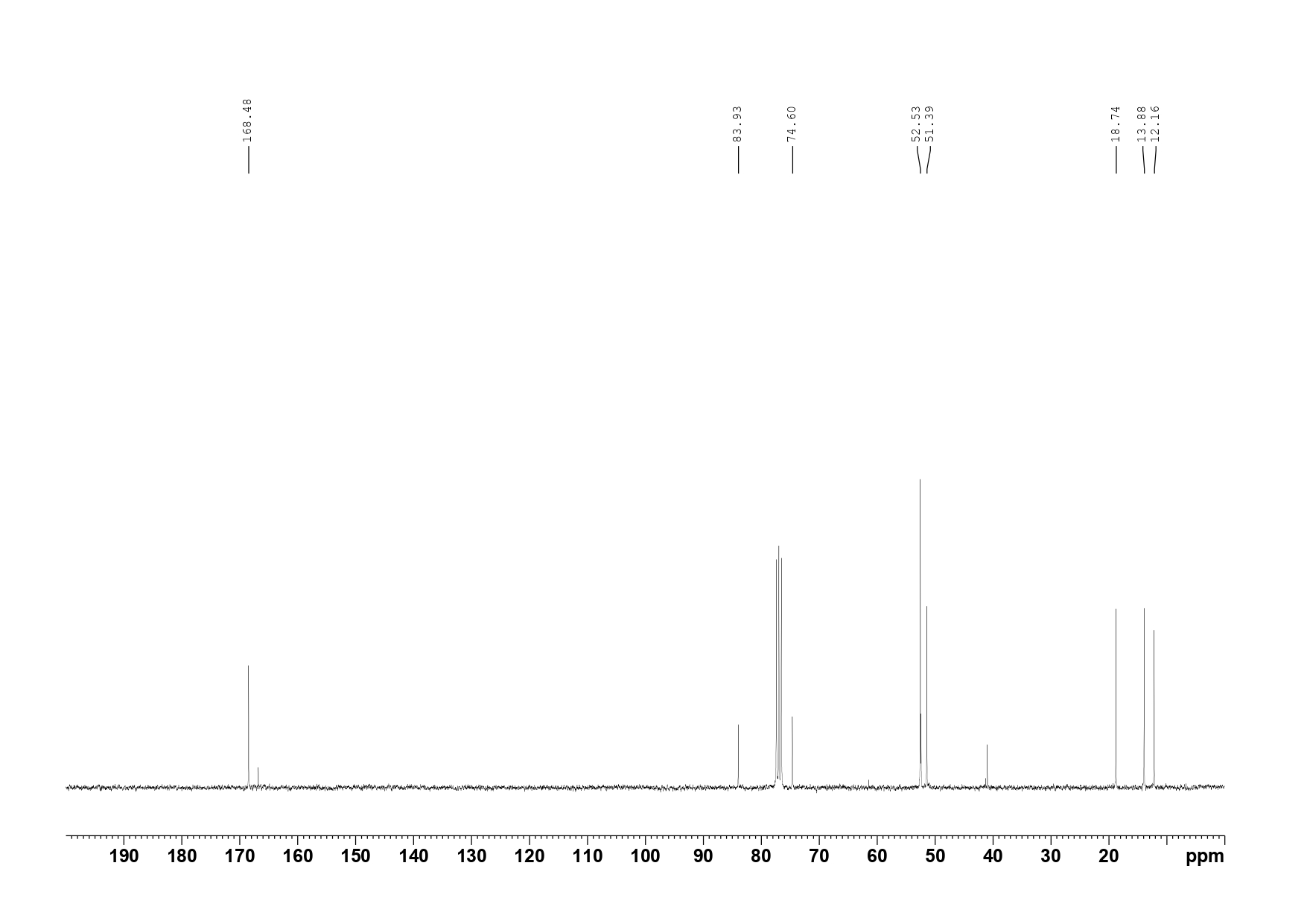
^

**1m**

^1^H NMR in CDCl_3_ (400 MHz)


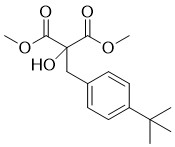
^
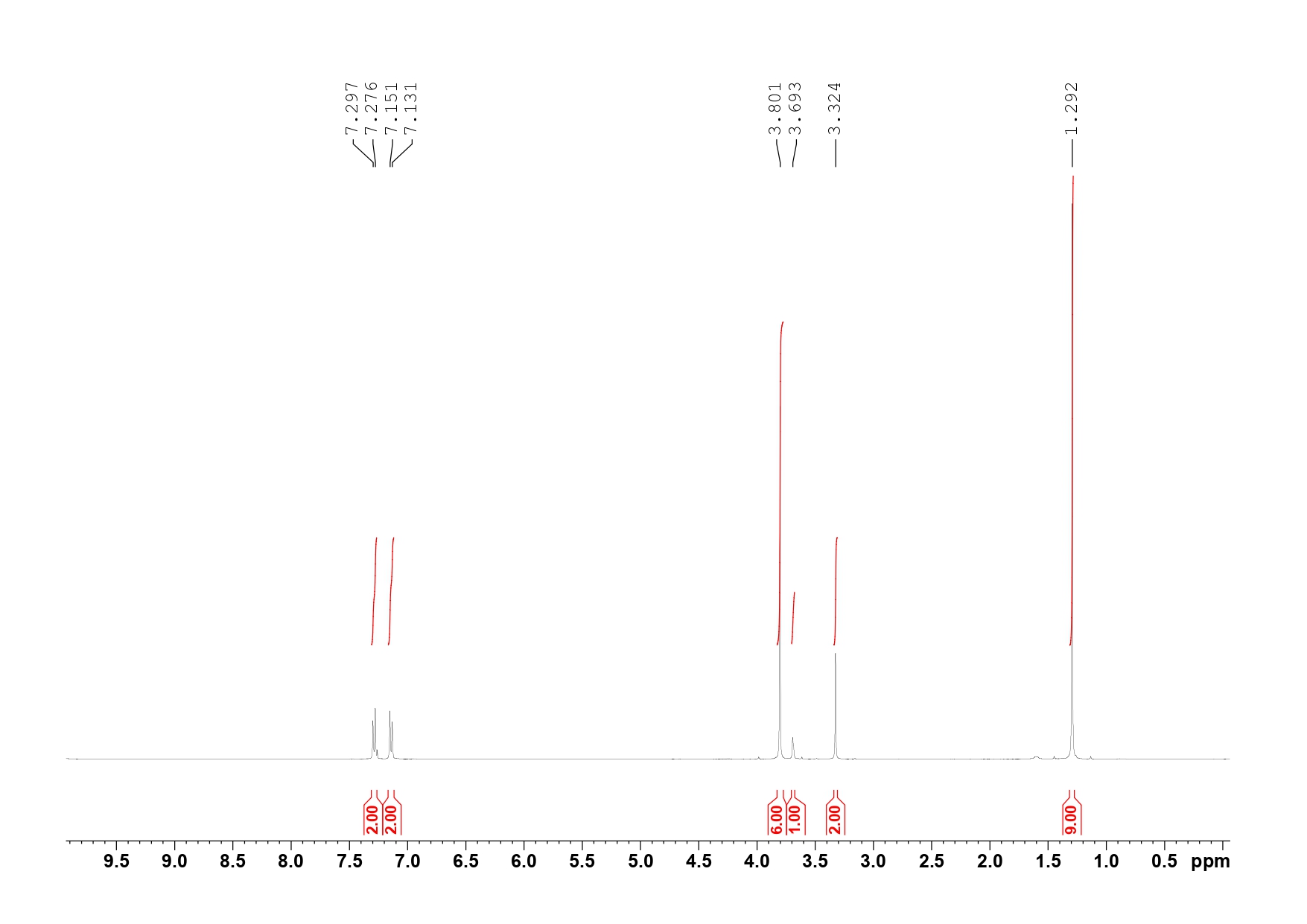
^

**2b**

^13^C NMR in CDCl_3_ (100 MHz)


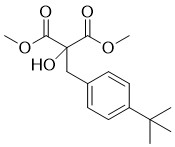

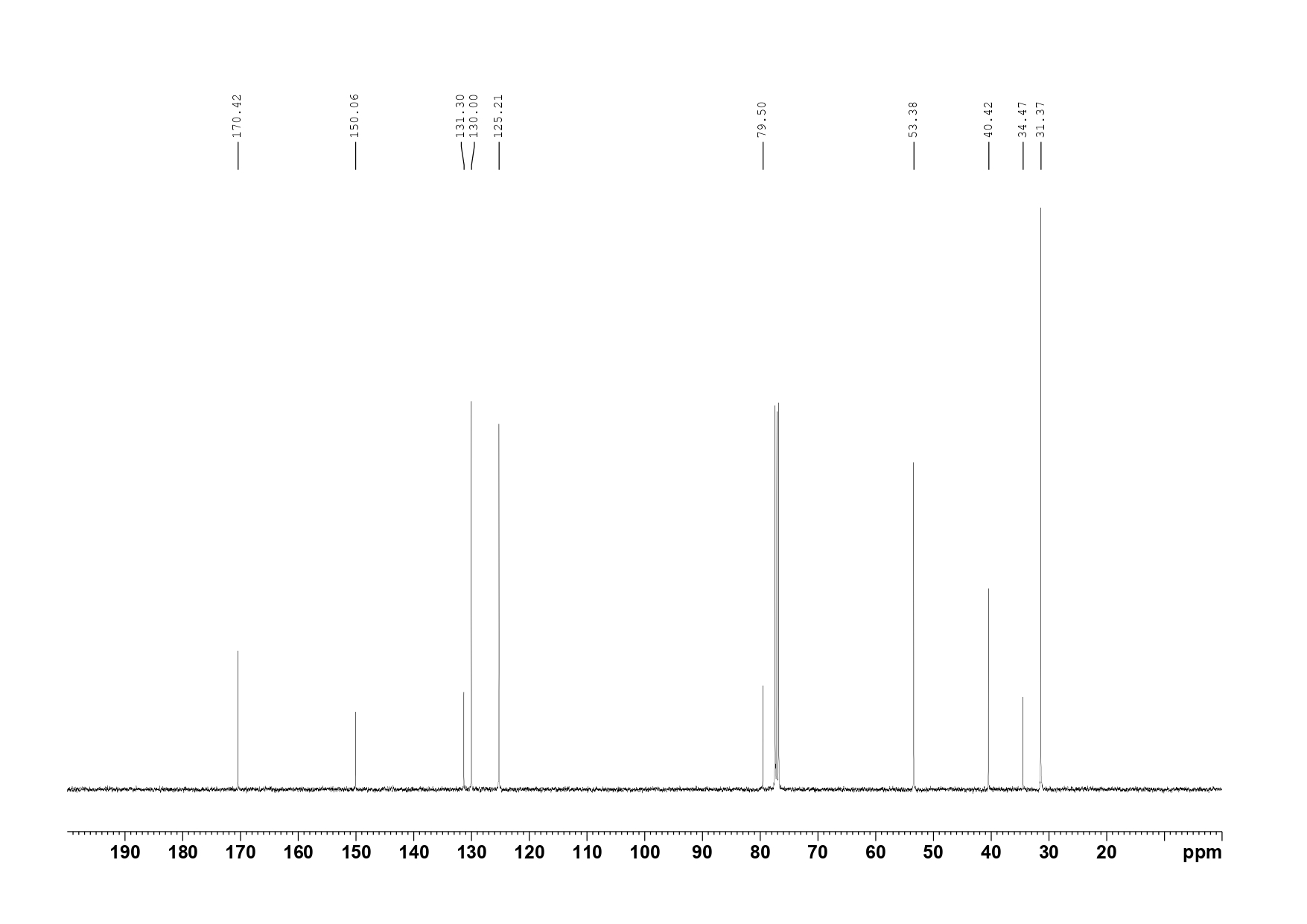


**2b**

^1^H NMR in CDCl_3_ (400 MHz)


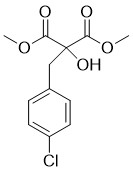


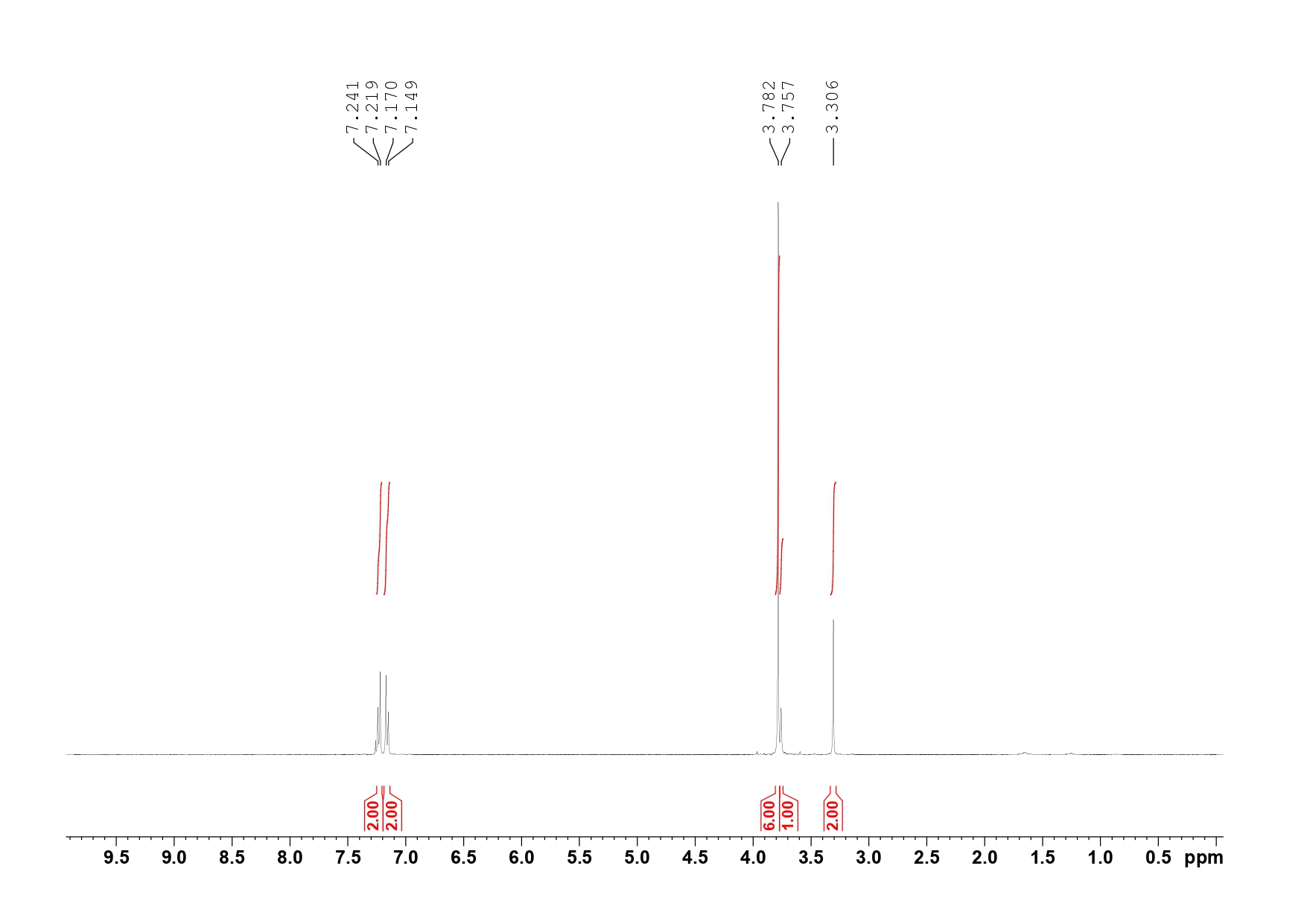


**2c**

^13^C NMR in CDCl_3_ (100 MHz)


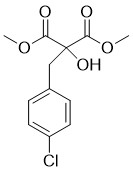

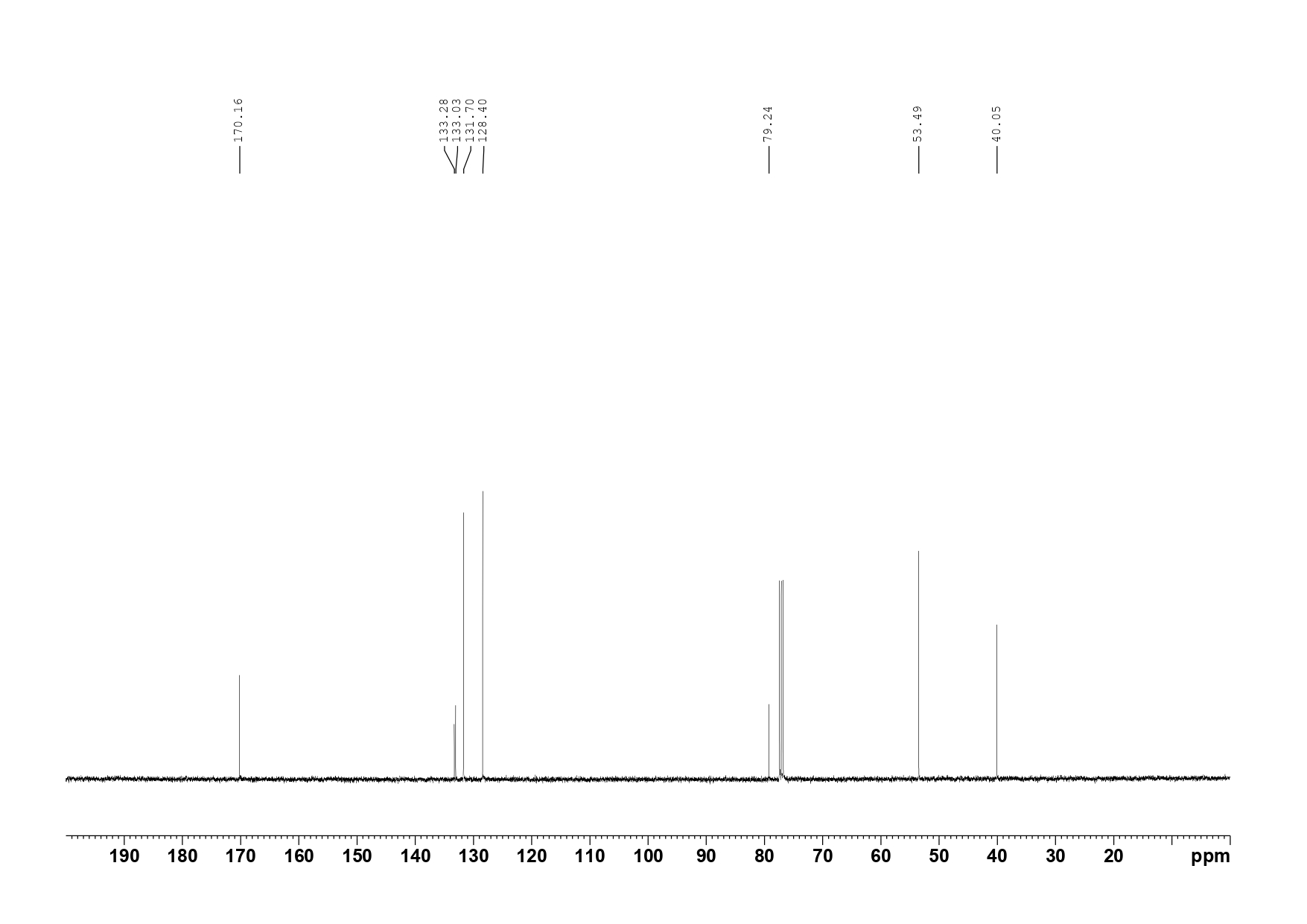


**2c**

^1^H NMR in CDCl_3_ (400 MHz)


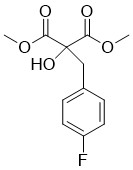

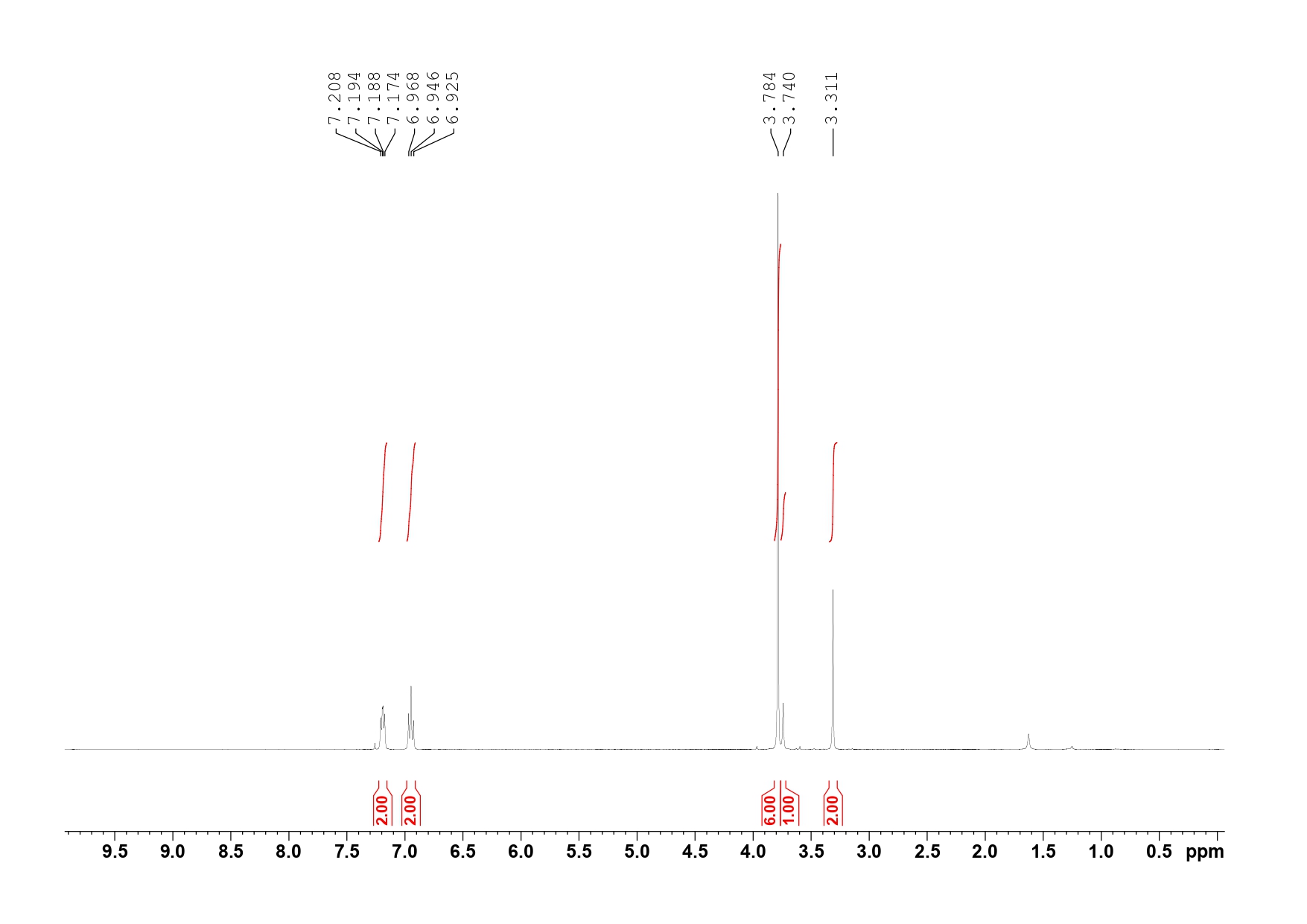


**2d**

^13^C NMR in CDCl_3_ (100 MHz)


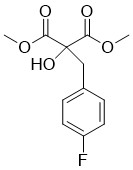
^
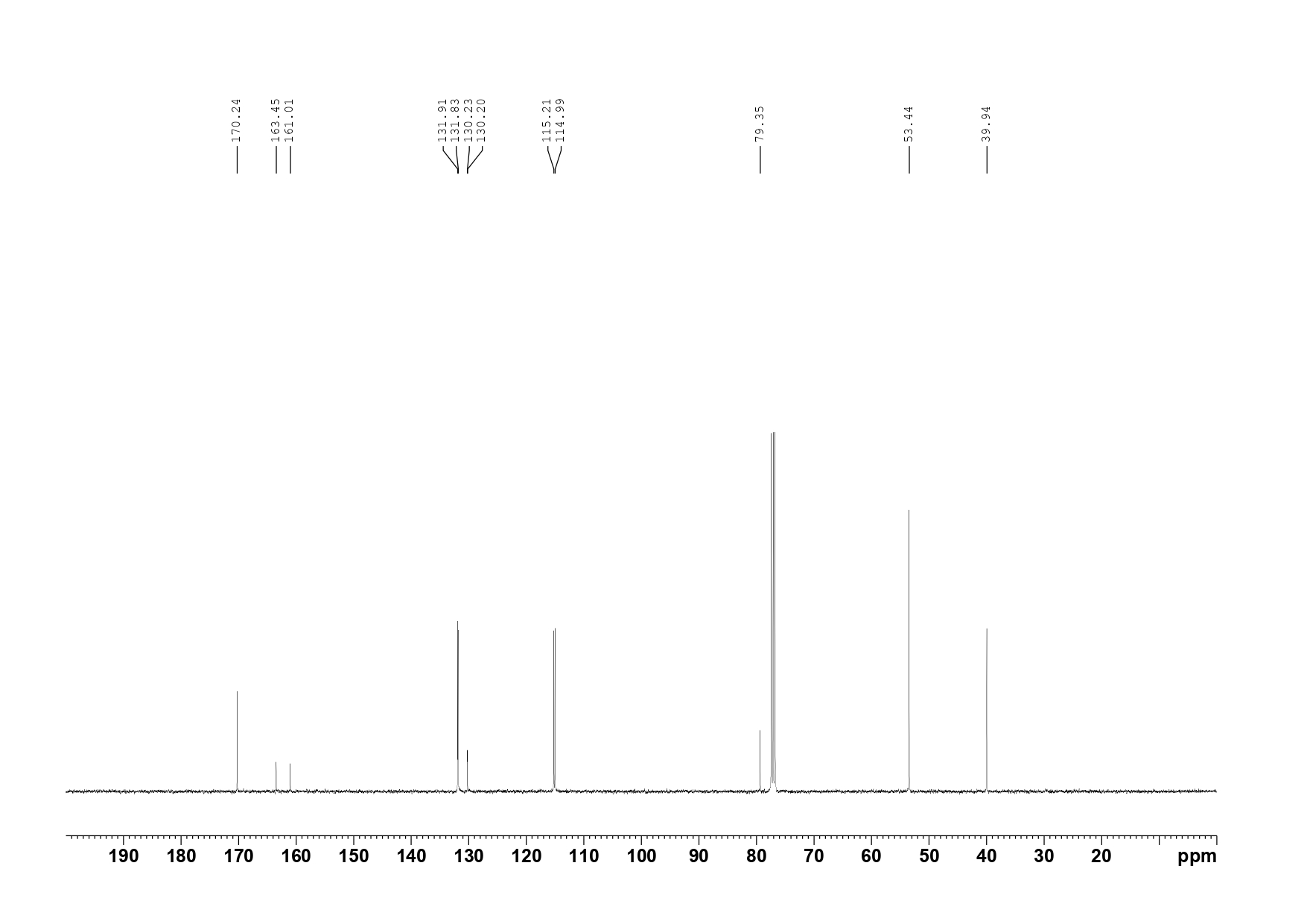
^

**2d**

^19^F NMR in CDCl_3_ (376 MHz)


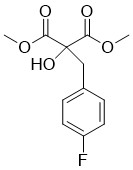
^
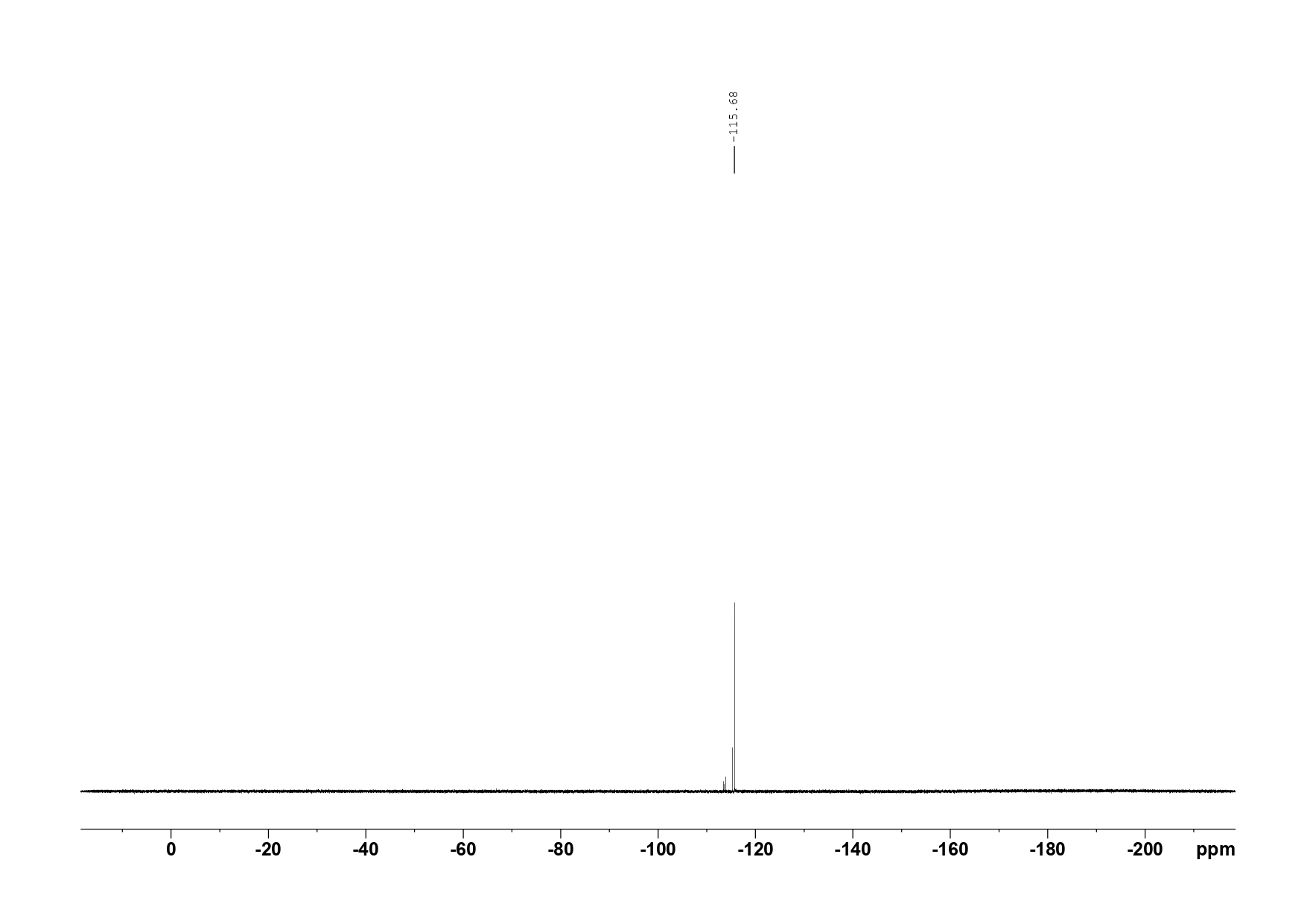
^

**2d**

^1^H NMR in CDCl_3_ (400 MHz)


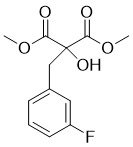
^
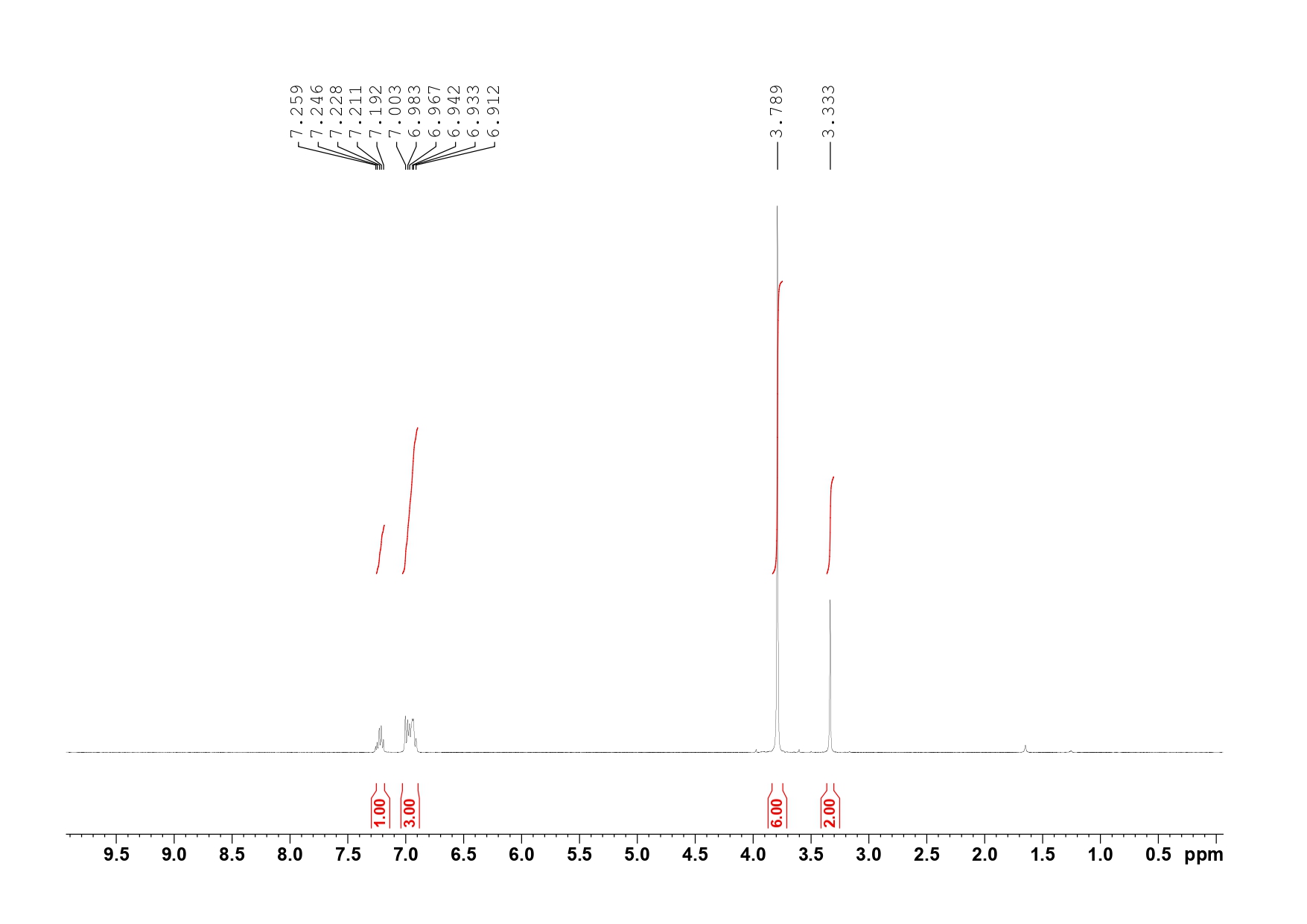
^

**2e**

^13^C NMR in CDCl_3_ (100 MHz)


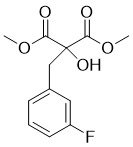

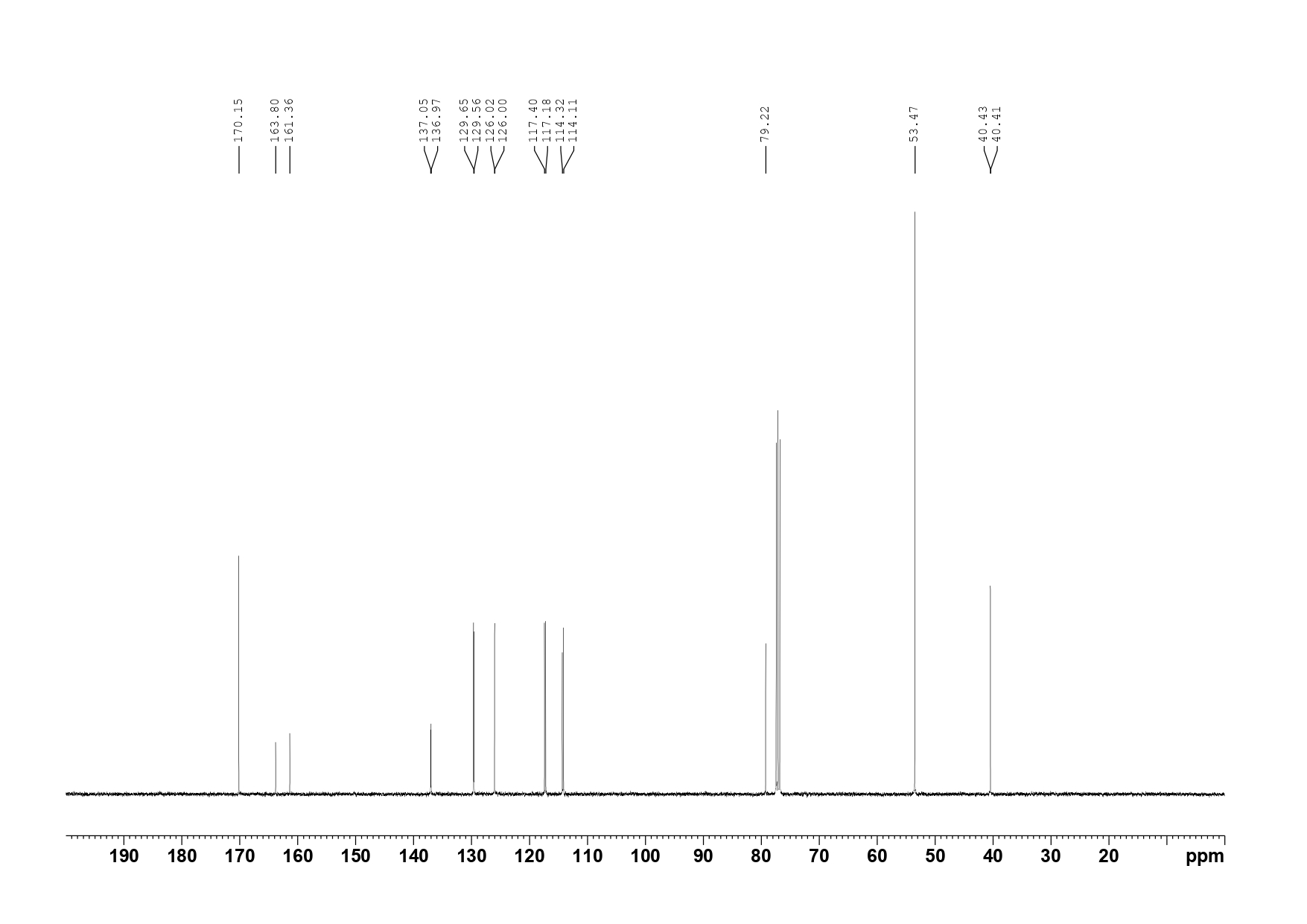


**2e**

^19^F NMR in CDCl_3_ (376 MHz)


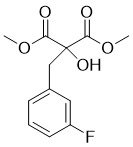


^
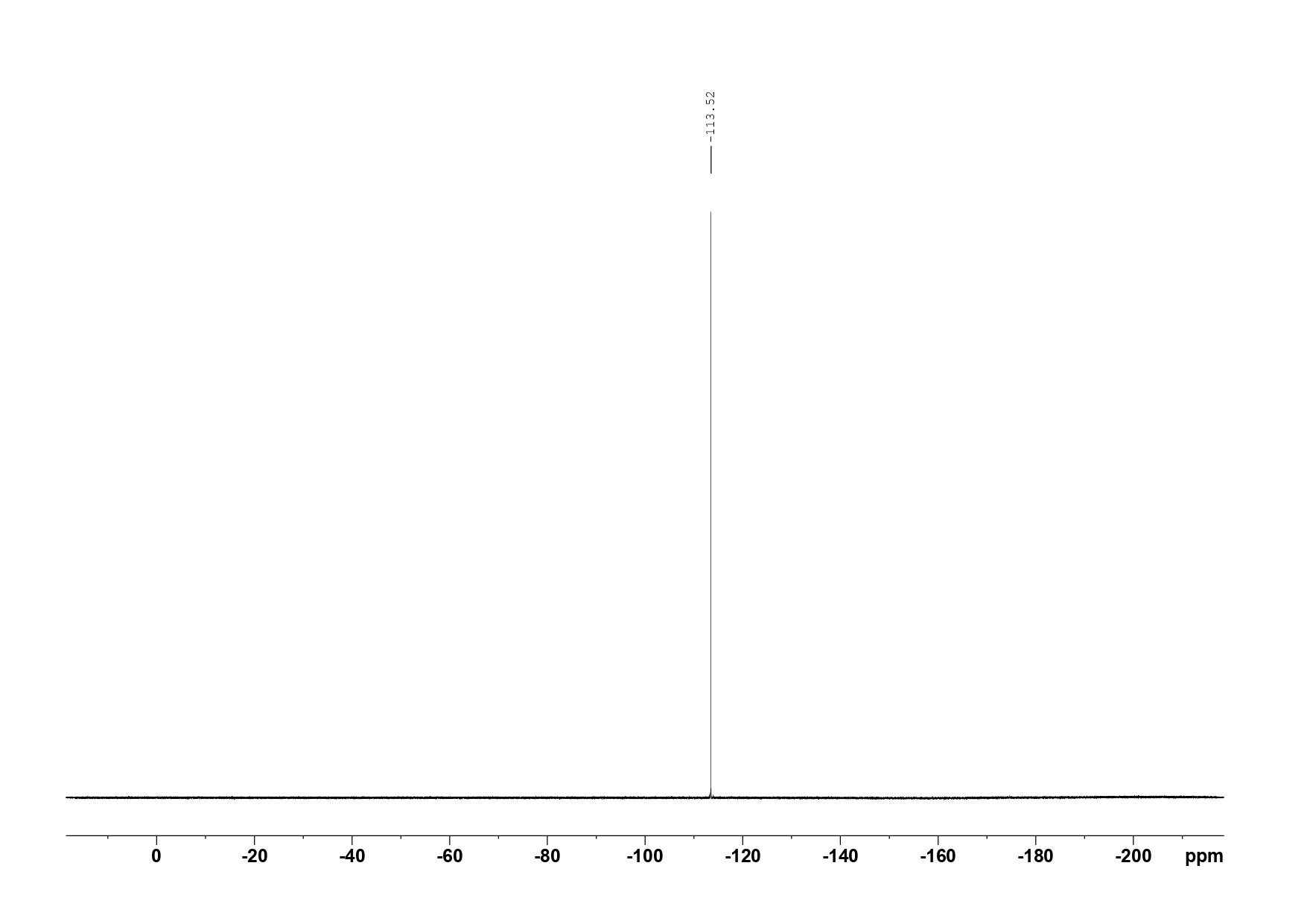
^

**2e**

^1^H NMR in CDCl_3_ (400 MHz)


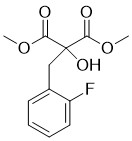

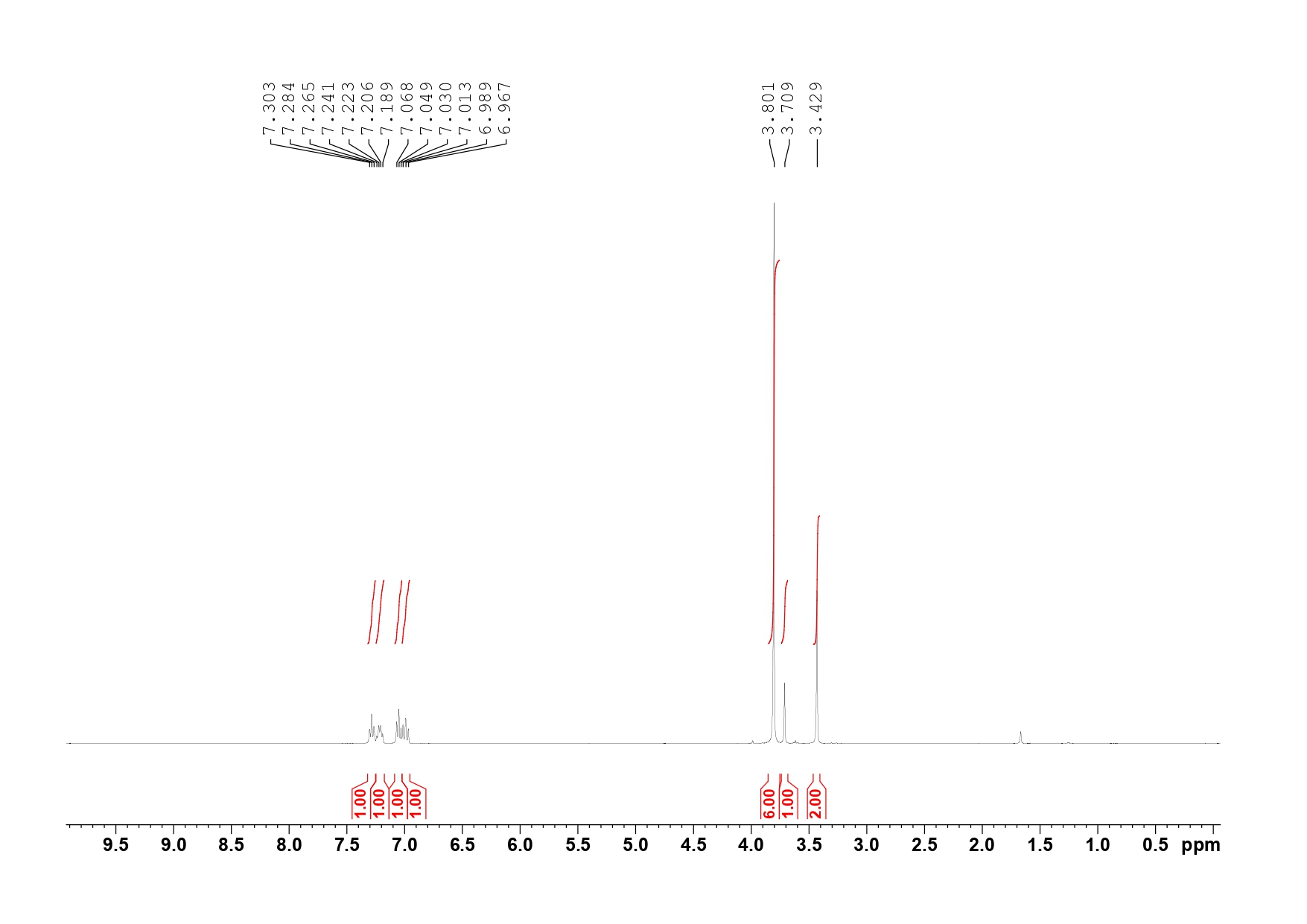


**2f**

^13^C NMR in CDCl_3_ (100 MHz)


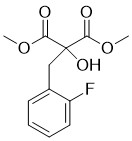

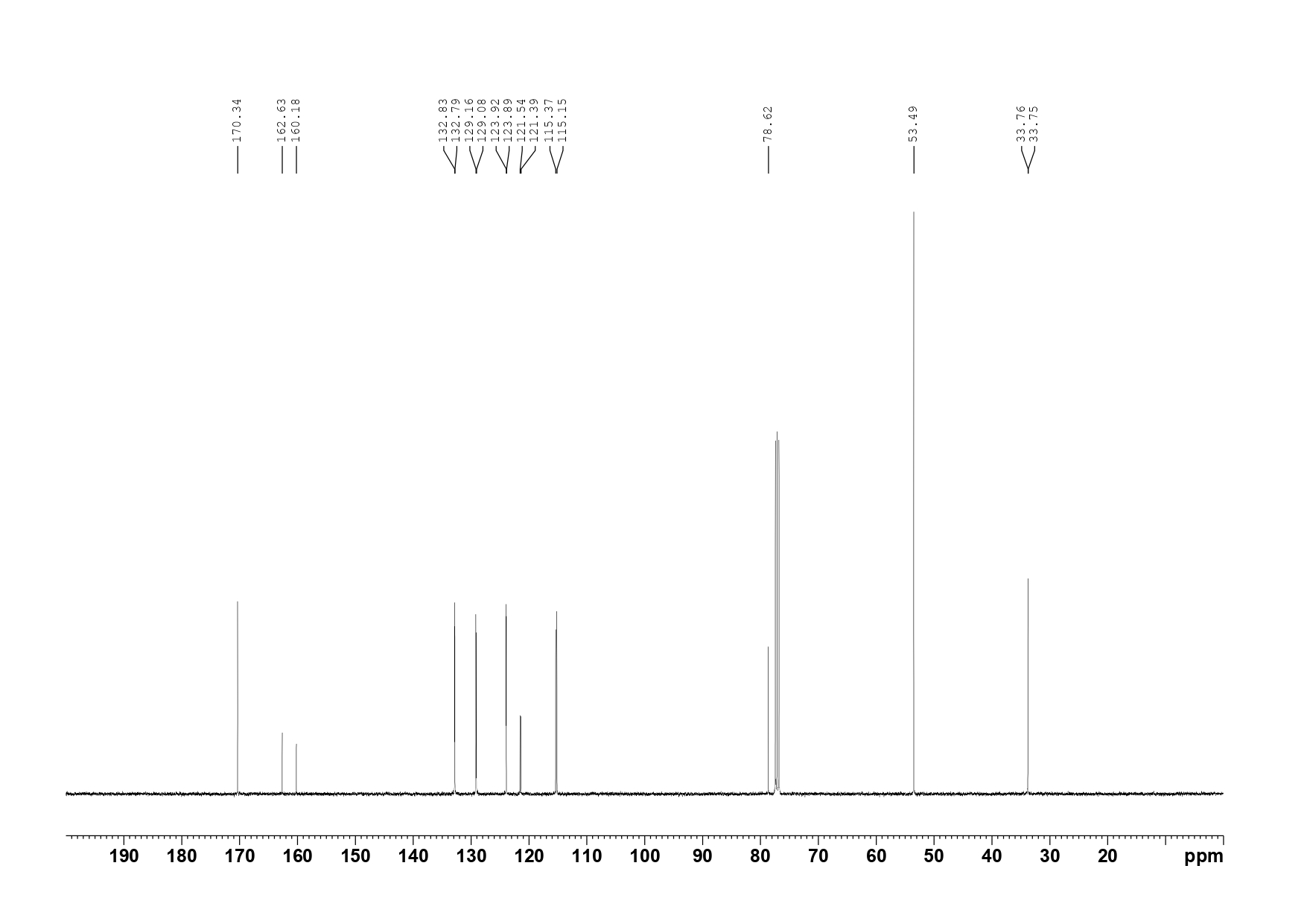


**2f**

^19^F NMR in CDCl_3_ (376 MHz)


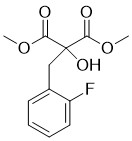


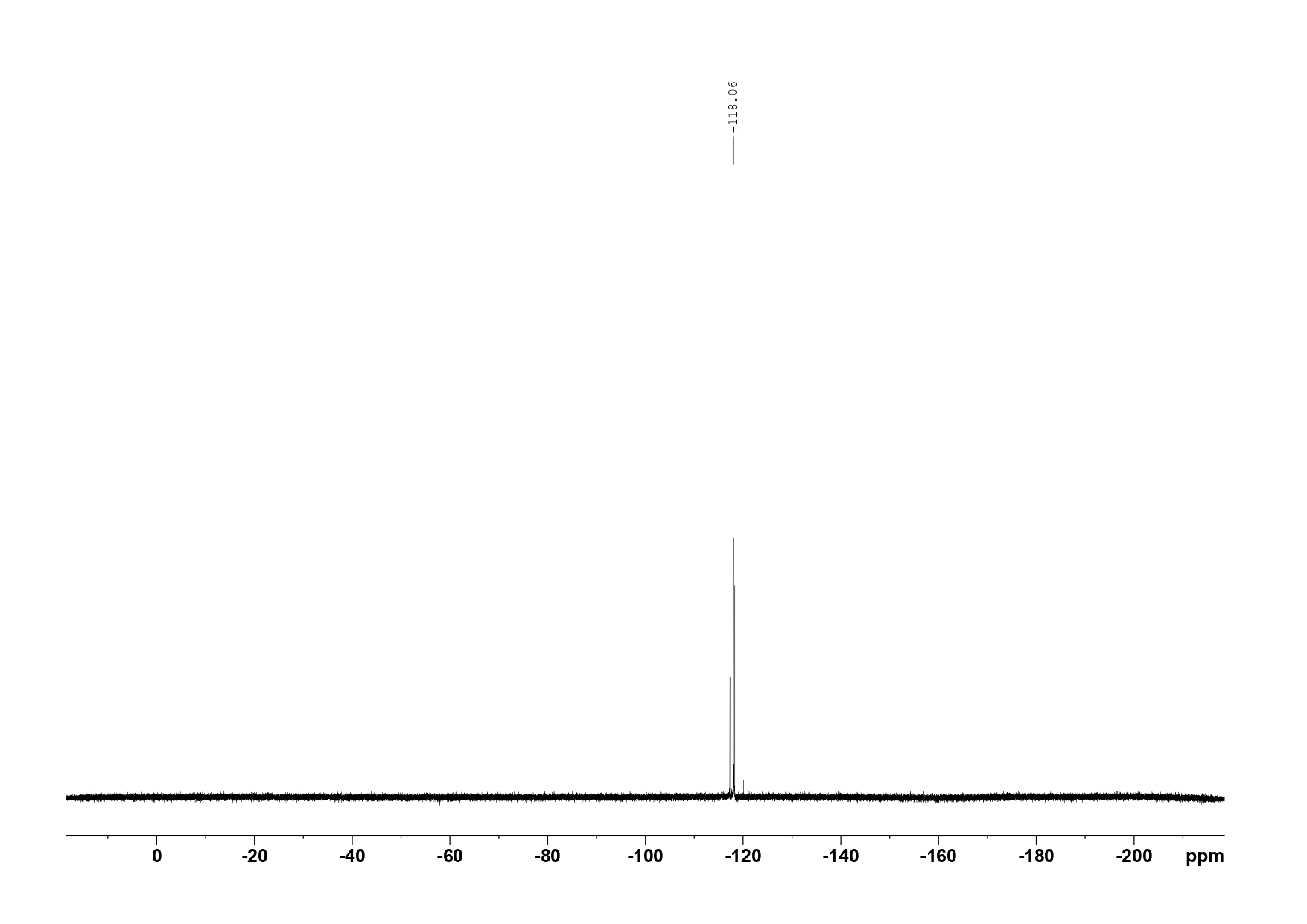


**2f**

^1^H NMR in CDCl_3_ (400 MHz)


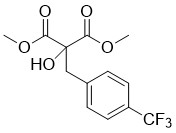
^
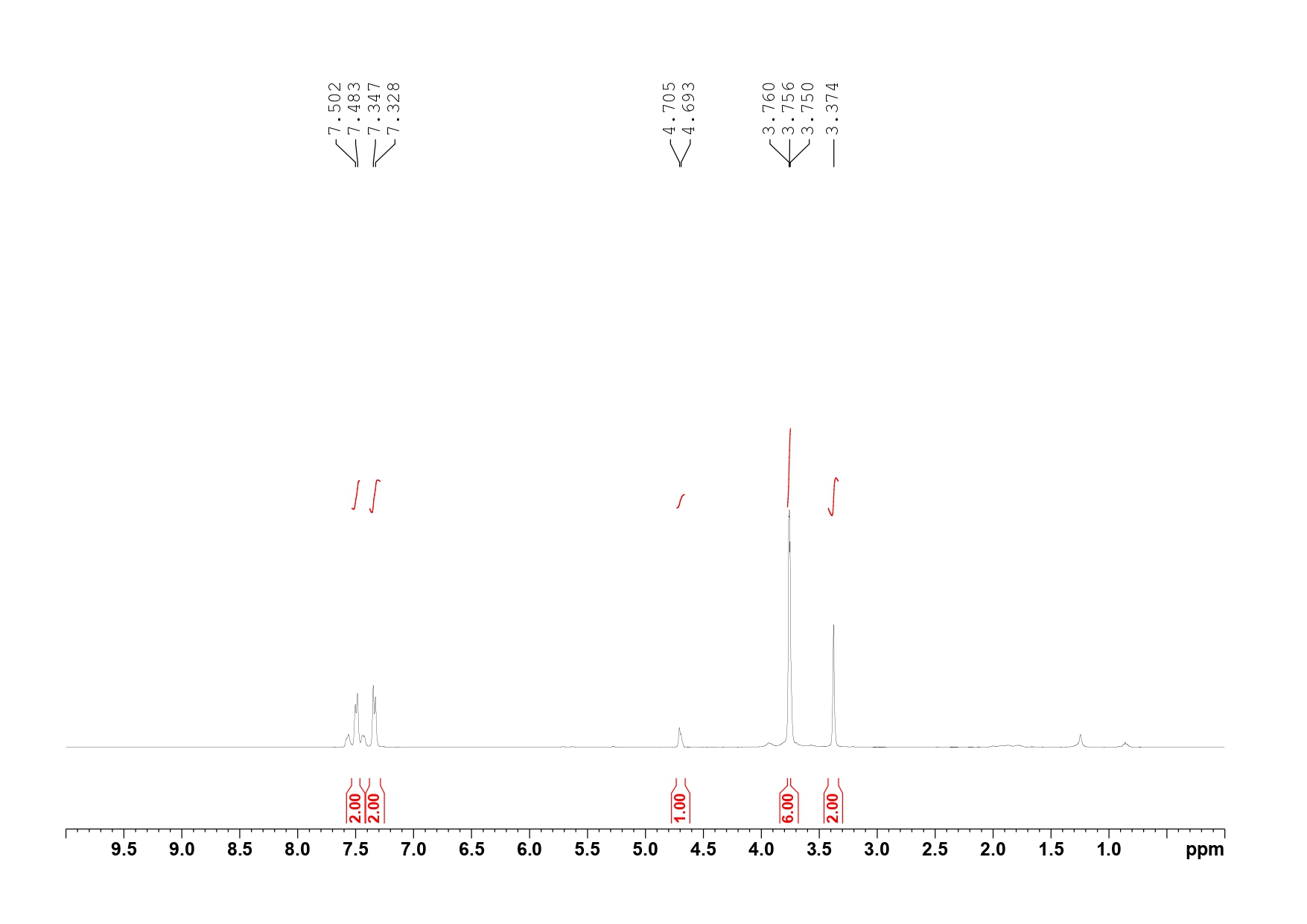
^

**2g**

^13^C NMR in CDCl_3_ (100 MHz)


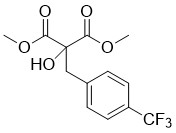


^
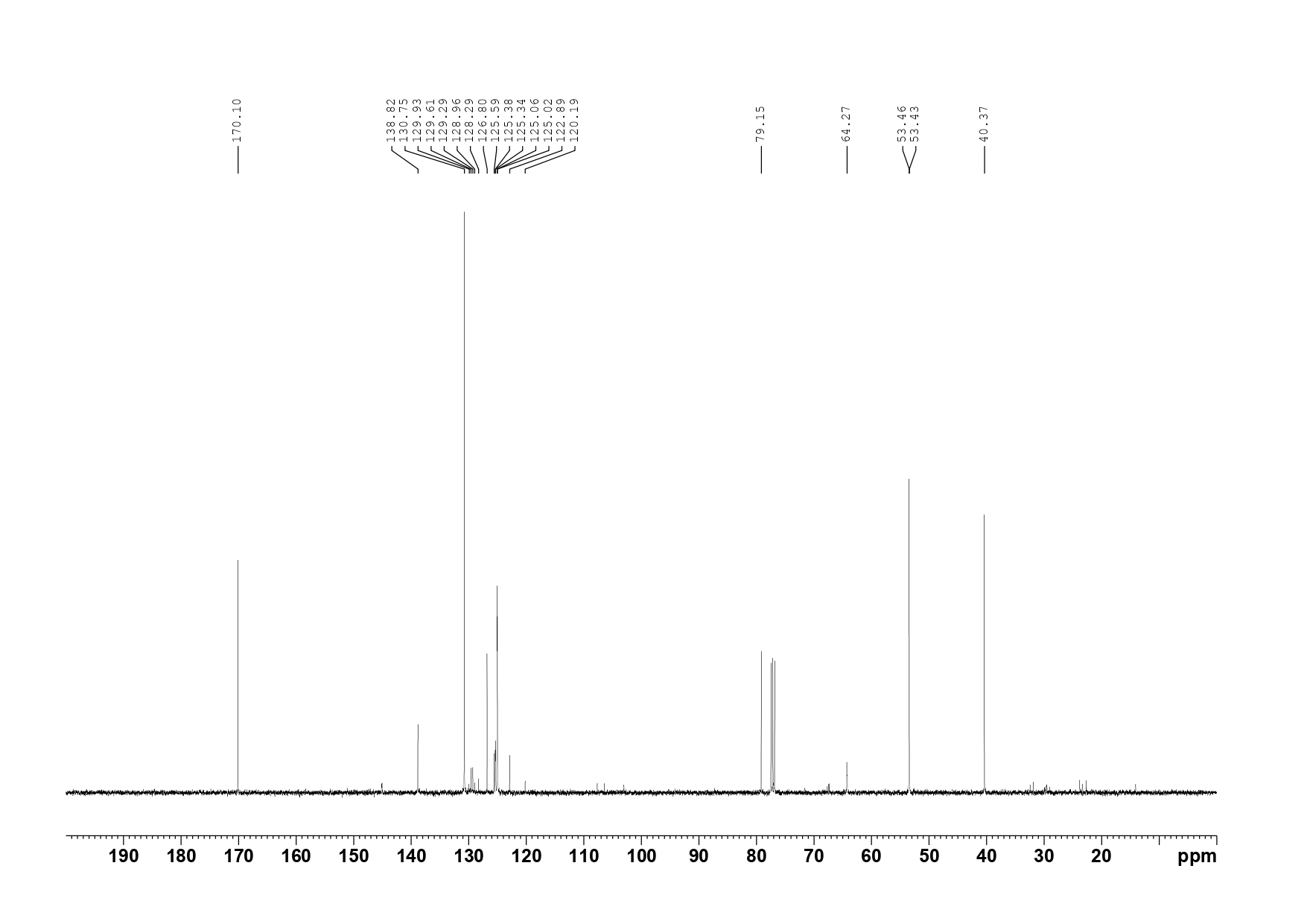
^

**2g**

^19^F NMR in CDCl_3_ (376 MHz)


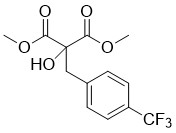


^
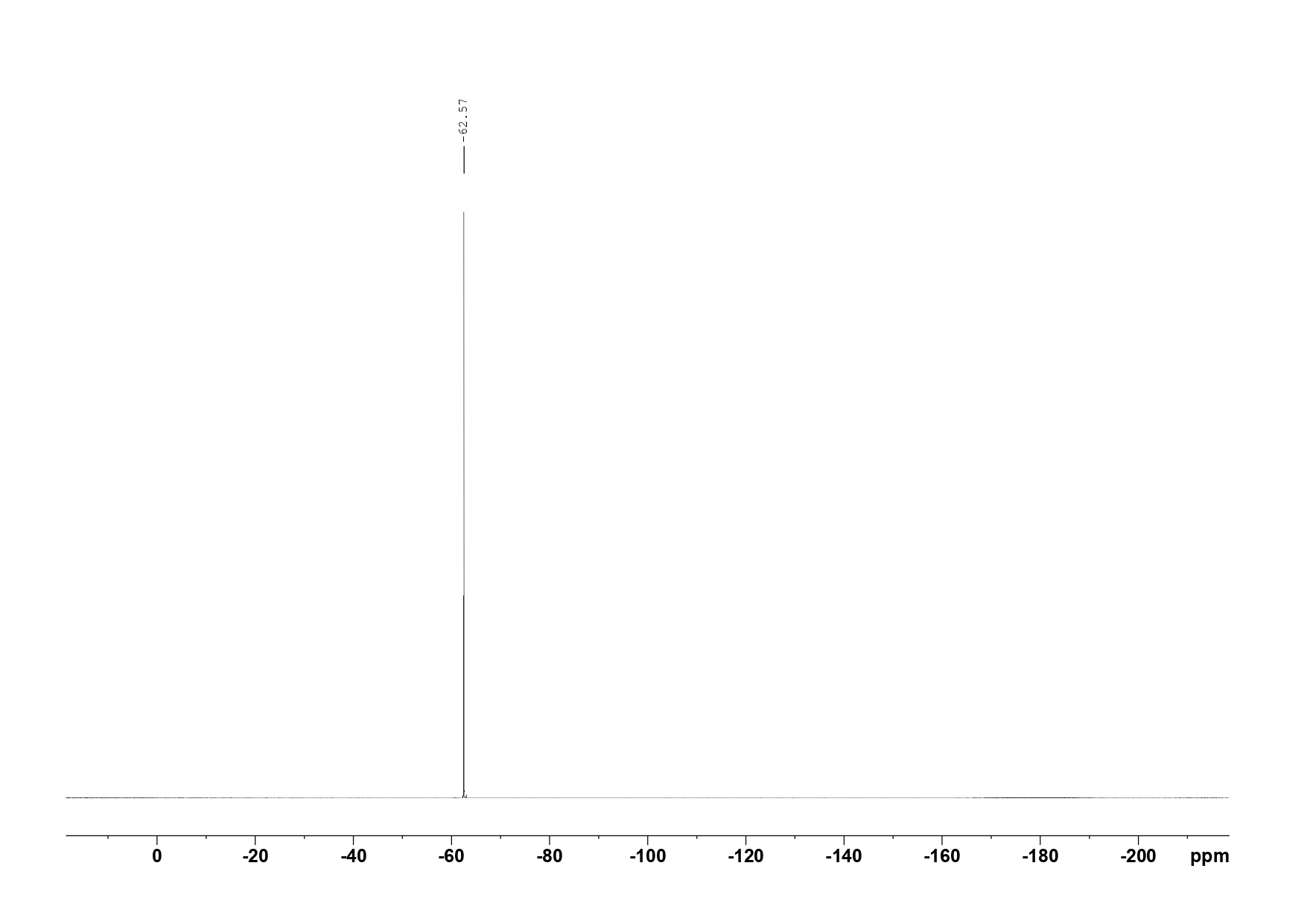
^

**2g**

^1^H NMR in CDCl_3_ (400 MHz)


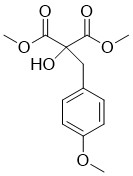

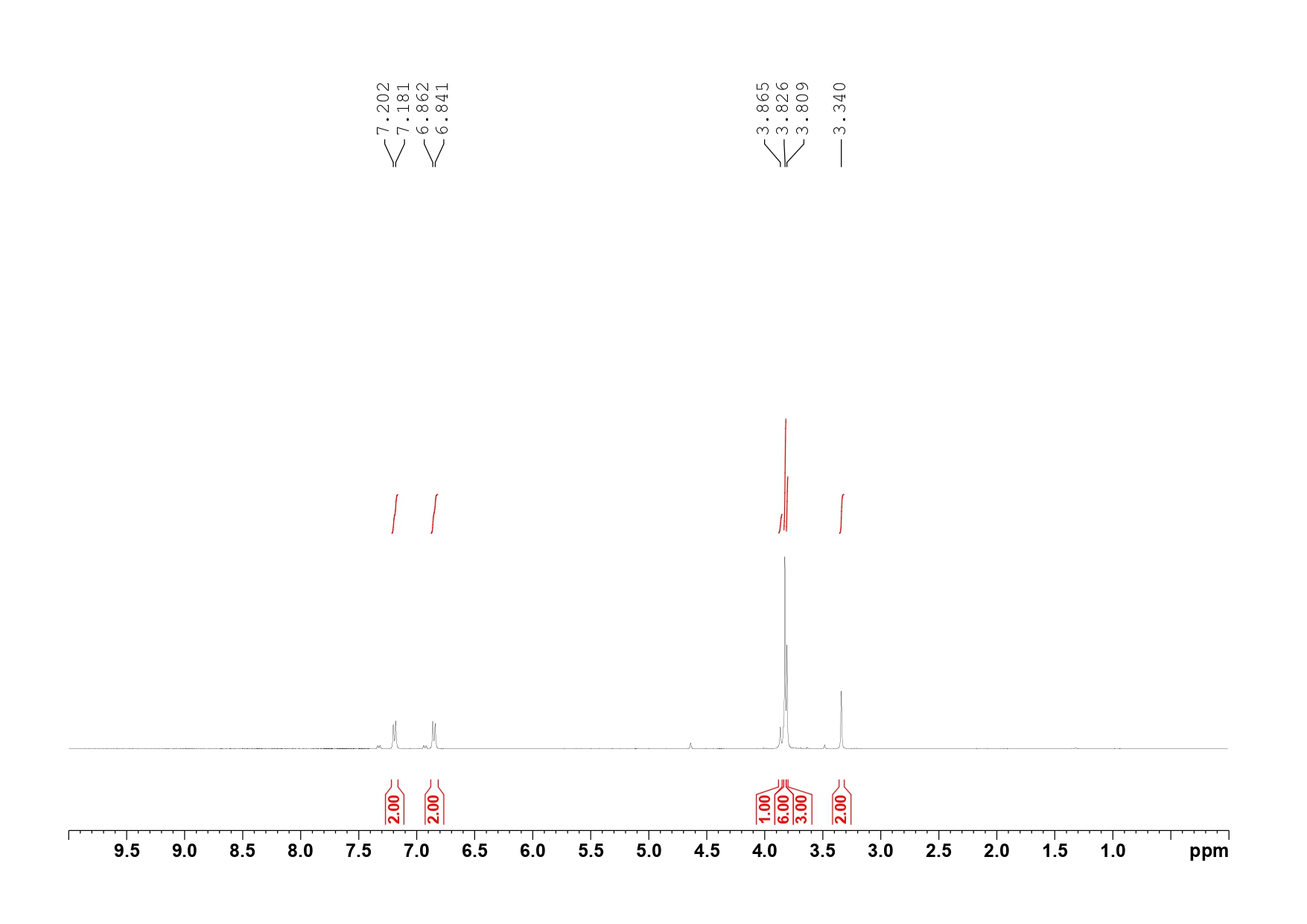


**2hKkkkkkmlkfmdmnkvlrmmkmK**

^13^C NMR in CDCl_3_ (100 MHz)


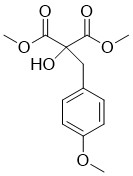
^
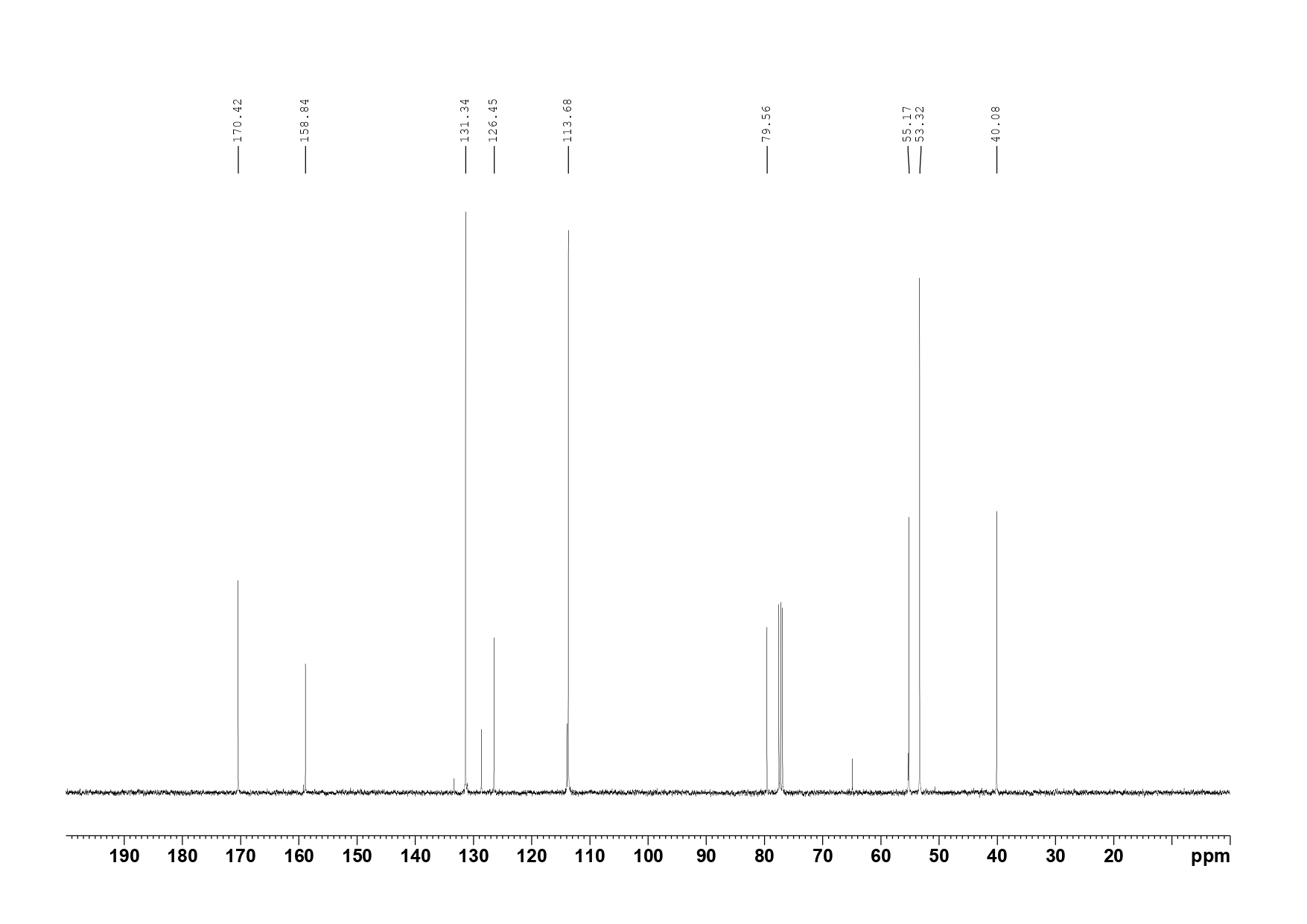
^

**2hKkkkkkmlkfmdmnkvlrmmkmK**

^1^H NMR in CDCl_3_ (300 MHz)


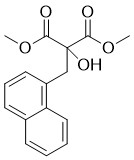
^
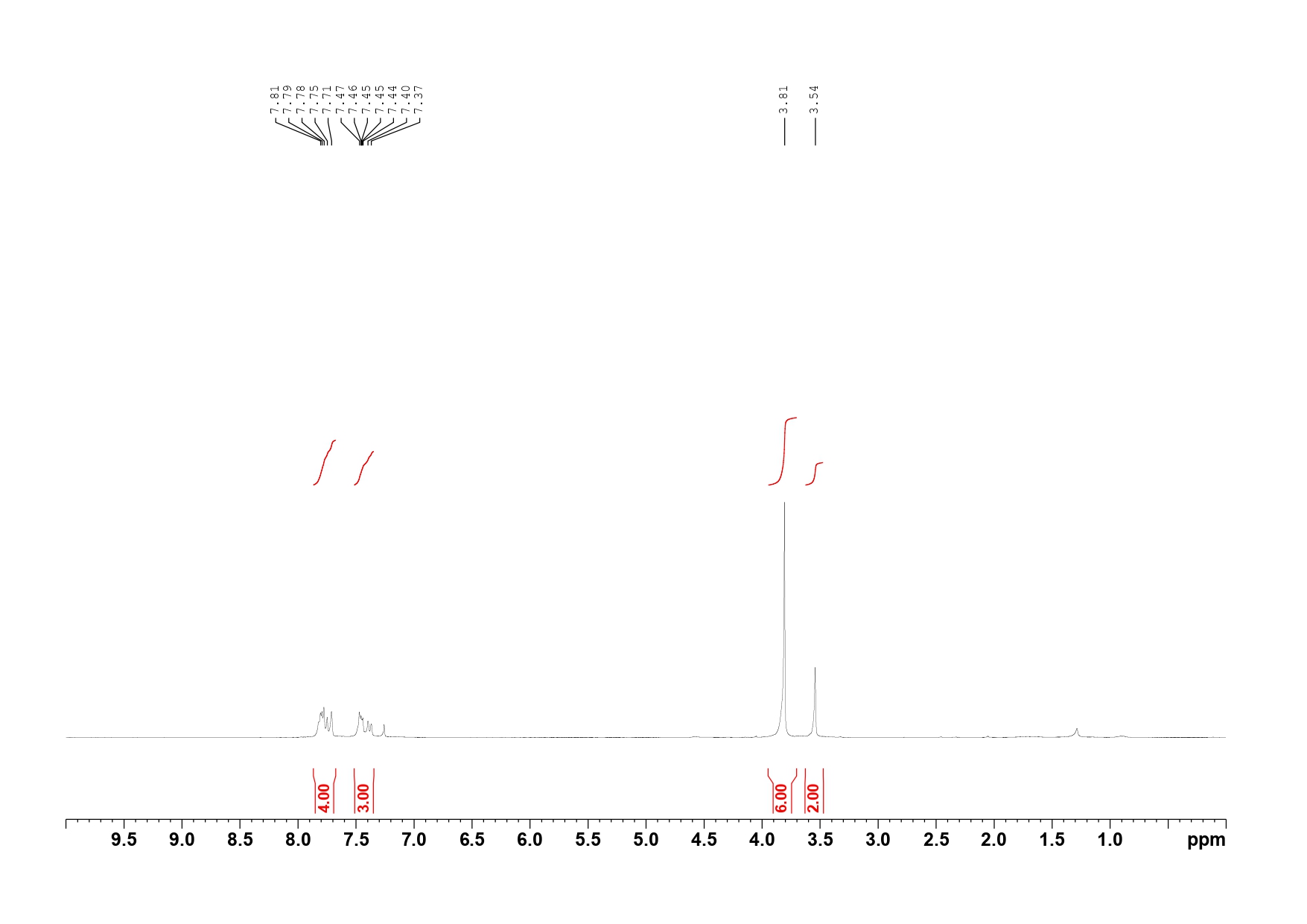
^

**2i**

^13^C NMR in CDCl_3_ (75 MHz)


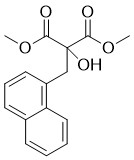
^
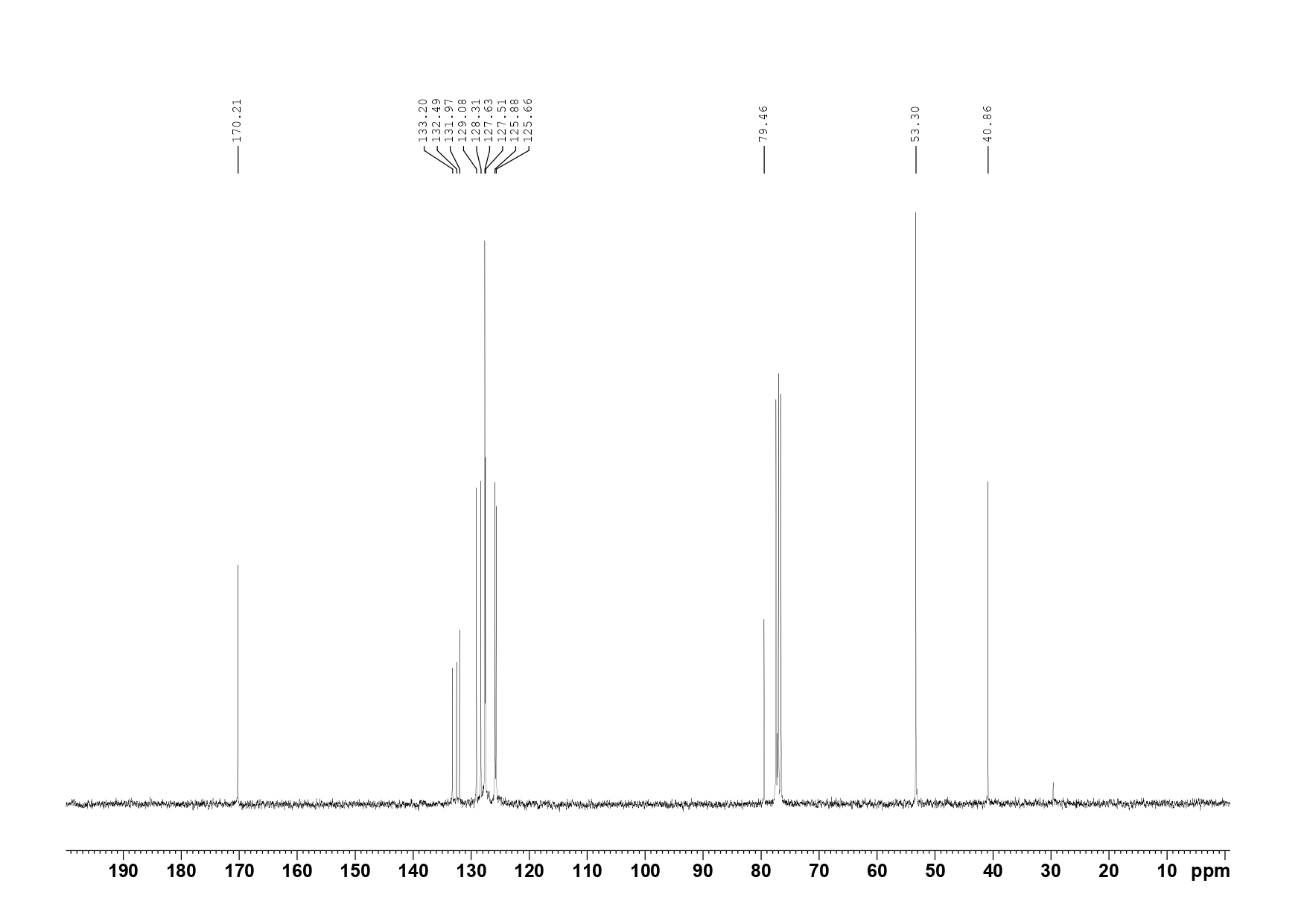
^

**2i**

^1^H NMR in CDCl_3_ (300 MHz)


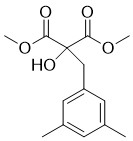
^
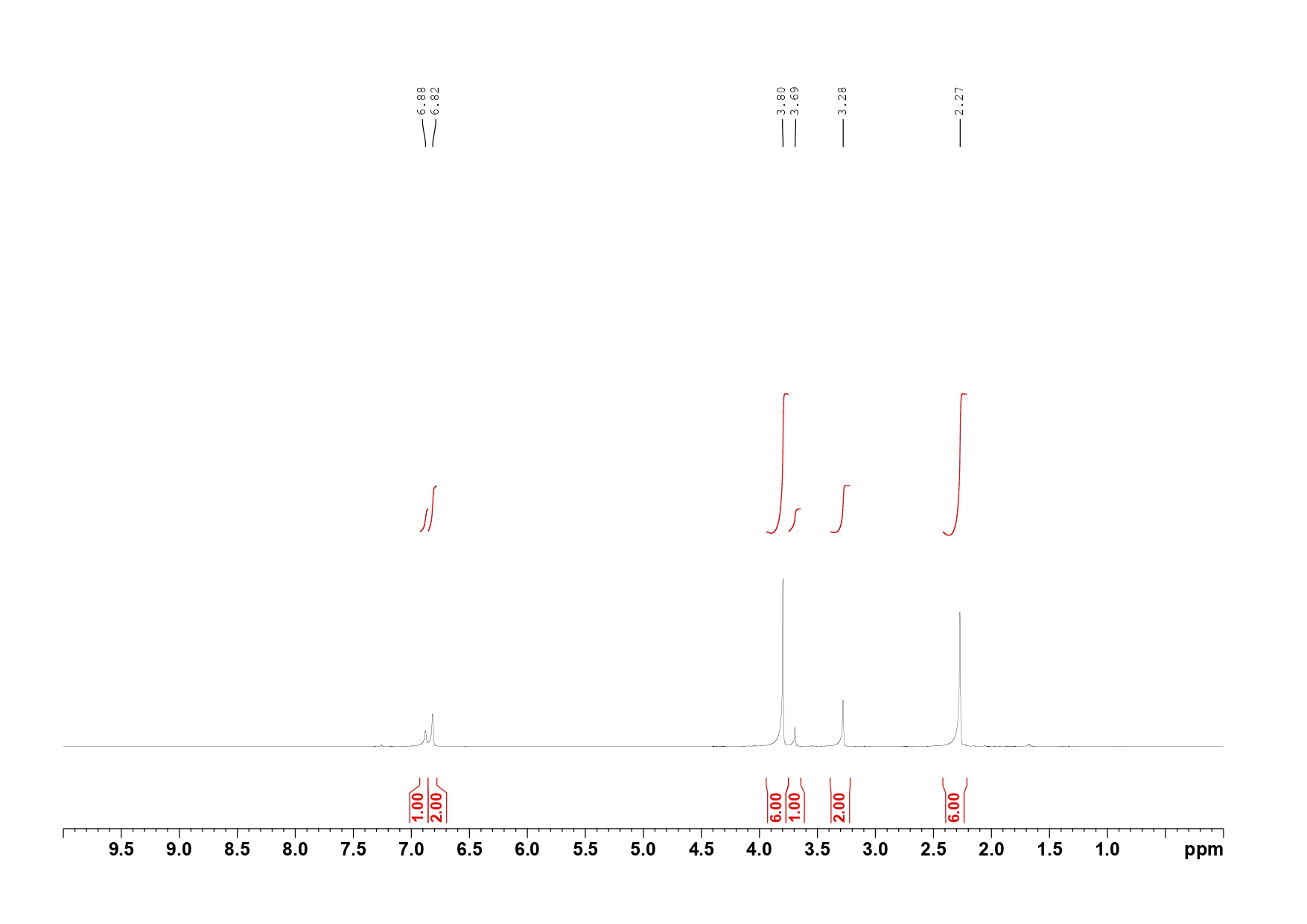
^

**2j**

^13^C NMR in CDCl_3_ (75 MHz)


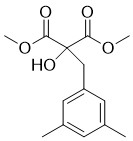

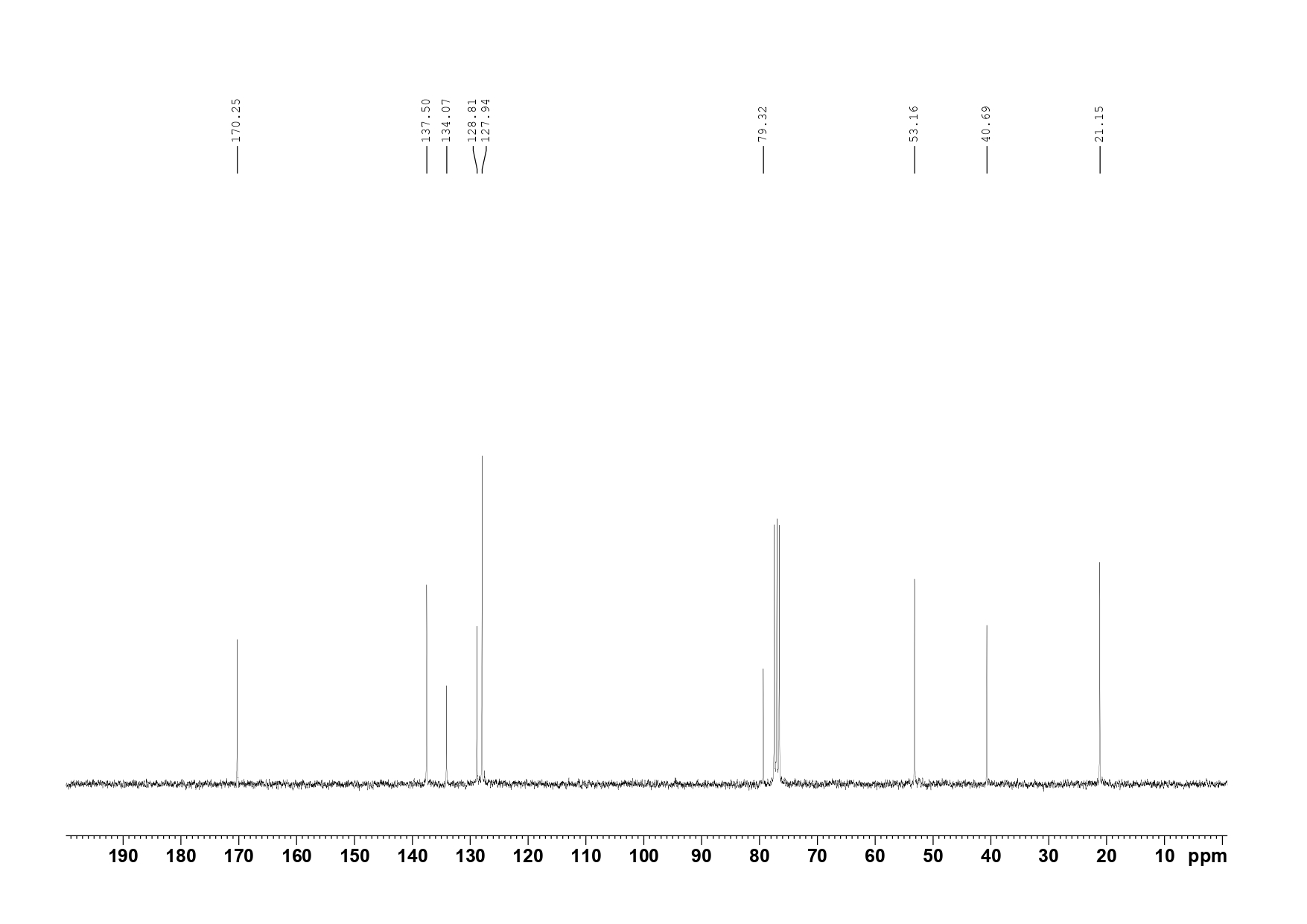


**2j**

^1^H NMR in CDCl_3_ (400 MHz)


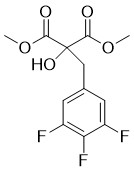
^
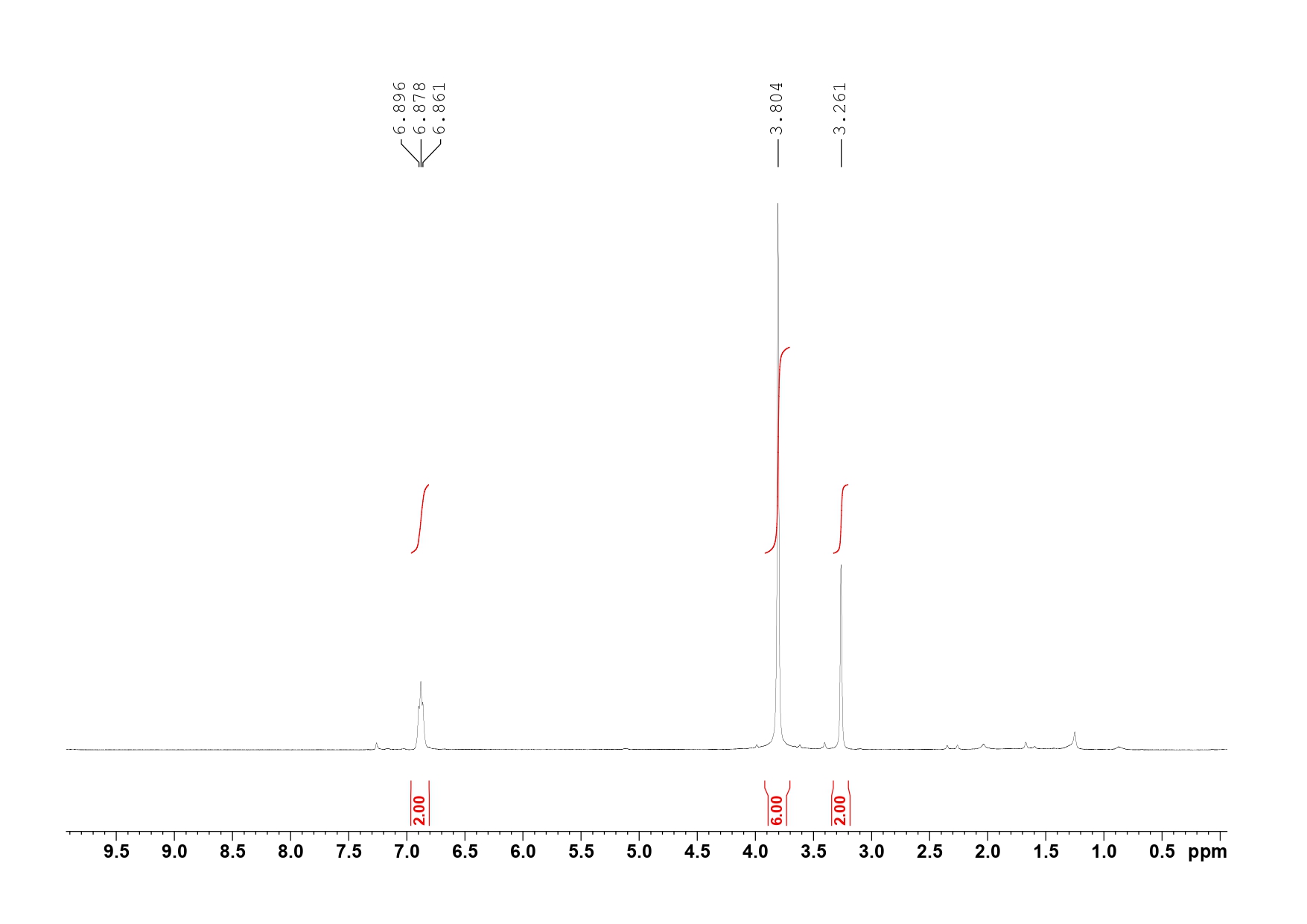
^

**2k**

^13^C NMR in CDCl_3_ (100 MHz)


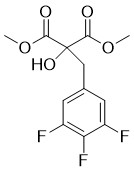


**2k**

^19^F NMR in CDCl_3_ (376 MHz)

**2k**

^1^H NMR in CDCl_3_ (300 MHz)

**2l**

^13^C NMR in CDCl_3_ (75 MHz)

**2l**

^1^H NMR in CDCl_3_ (300 MHz)

**2m**

^13^C NMR in CDCl_3_ (75 MHz)

**2m**

^1^H NMR in CDCl_3_ (400 MHz)

**3o**

^13^C NMR in CDCl_3_ (62.5 MHz)

**3o**

^1^H NMR in CDCl_3_ (600 MHz)

**3p**

^13^C NMR in CDCl_3_ (150 MHz)

**3p**

^19^F NMR in CDCl_3_ (376 MHz)

**3p**

^1^H NMR in CDCl_3_ (300 MHz)

**3q**

**c**

^13^C NMR in CDCl_3_ (62.5 MHz)

**3q**

**c**

^1^H NMR in CDCl_3_ (300 MHz)

**3a**

^13^C NMR in CDCl_3_ (62.5 MHz)

**3a**

^1^H NMR in CDCl_3_ (300 MHz)

**3b**

^13^C NMR in CDCl_3_ (75 MHz)

**3b**

^1^H NMR in CDCl_3_ (400 MHz)

**3c**

^13^C NMR in CDCl_3_ (100 MHz)

**3c**

^1^H NMR in CDCl_3_ (400 MHz)

**3d**

^13^C NMR in CDCl_3_ (100 MHz)

**3d**

^19^F NMR in CDCl_3_ (376 MHz)

**3d**

^1^H NMR in CDCl_3_ (400 MHz)

**3e**

^13^C NMR in CDCl_3_ (100 MHz)

**3e**

^19^F NMR in CDCl_3_ (376 MHz)

**3e**

^1^H NMR in CDCl_3_ (400 MHz)

**3f**

^13^C NMR in CDCl_3_ (100 MHz)

**3f**

^19^F NMR in CDCl_3_ (376 MHz)

**3f**

^1^H NMR in CDCl_3_ (400 MHz)

**3g**

^13^C NMR in CDCl_3_ (100 MHz)

**3g**

^19^F NMR in CDCl_3_ (376 MHz)

**3g**

^1^H NMR in CDCl_3_ (400 MHz)

**3h**

^13^C NMR in CDCl_3_ (100 MHz)

**3h**

^1^H NMR in CDCl_3_ (300 MHz)

**3i**

^13^C NMR in CDCl_3_ (75 MHz)

**3i**

^1^H NMR in CDCl_3_ (300 MHz)

**3j**

^13^C NMR in CDCl_3_ (75 MHz)

**3j**

^1^H NMR in CDCl_3_ (400 MHz)

**3k**

^13^C NMR in CDCl_3_ (100 MHz)

**3k**

^19^F NMR in CDCl_3_ (376 MHz)

**3k**

^1^H NMR in CDCl_3_ (300 MHz)

**3l**

^13^C NMR in CDCl_3_ (75 MHz)

**3l**

^1^H NMR in CDCl_3_ (400 MHz)

**3m**

^13^C NMR in CDCl_3_ (100 MHz)

**3m**

^1^H NMR in CDCl_3_ (400 MHz)

**3n**

^13^C NMR in CDCl_3_ (100 MHz)

**3n**

^1^H NMR in CDCl_3_ (250 MHz)

**4o**

^13^C NMR in CDCl_3_ (62.5 MHz)

**4o**

^1^H NMR in CDCl_3_ (600 MHz)

**4p**

^13^C NMR in CDCl_3_ (150 MHz)

**4p**

^19^F NMR in CDCl_3_ (376 MHz)

**4p**

^1^H NMR in CDCl_3_ (600 MHz)

**4q**

^13^C NMR in CDCl_3_ (150 MHz)

**4q**

^1^H NMR in CDCl_3_ (300 MHz)

**4a**

^13^C NMR in CDCl_3_ (75 MHz)

**4a**

^1^H NMR in CDCl_3_ (400 MHz)

**4b**

^13^C NMR in CDCl_3_ (100 MHz)

**4b**

^1^H NMR in CDCl_3_ (400 MHz)

**4c**

^13^C NMR in CDCl_3_ (62.5 MHz)

**4c**

^1^H NMR in CDCl_3_ (400 MHz)

**4d**

^13^C NMR in CDCl_3_ (100 MHz)

**4d**

^19^F NMR in CDCl_3_ (376 MHz)

**4d**

^1^H NMR in CDCl_3_ (400 MHz)

**4e**

^13^C NMR in CDCl_3_ (100 MHz)

**4e**

^19^F NMR in CDCl_3_ (376 MHz)

**4e**

^1^H NMR in CDCl_3_ (400 MHz)

**4f**

^13^C NMR in CDCl_3_ (100 MHz)

**4f**

^19^F NMR in CDCl_3_ (376 MHz)

**4f**

^1^H NMR in CDCl_3_ (400 MHz)

**4g**

^13^C NMR in CDCl_3_ (100 MHz)

**4g**

^19^F NMR in CDCl_3_ (376 MHz)

**4g**

^1^H NMR in CDCl_3_ (400 MHz)

**4i**

^13^C NMR in CDCl_3_ (62.5 MHz)

**4i**

^1^H NMR in CDCl_3_ (300 MHz)

**4j**

^13^C NMR in CDCl_3_ (75 MHz)

**4j**

^1^H NMR in CDCl_3_ (600 MHz)

**4k**

^13^C NMR in CDCl_3_ (150 MHz)

**4k**

^19^F NMR in CDCl_3_ (564 MHz)

**4k**

^1^H NMR in CDCl_3_ (250 MHz)

**4l**

^13^C NMR in CDCl_3_ (62.5 MHz)

**4l**

^1^H NMR in CDCl_3_ (300 MHz)

**4m**

^13^C NMR in CDCl_3_ (75 MHz)

**4m**

^1^H NMR in CDCl_3_ (300 MHz)

**4n**

^13^C NMR in CDCl_3_ (62.5 MHz)

**4n**

^1^H NMR in CDCl_3_ (600 MHz)

**3a’**

^13^C NMR in CDCl_3_ (150 MHz)

**3a’**

^1^H NMR in CDCl_3_ (400 MHz)

**14**

^13^C NMR in CDCl_3_ (100 MHz)

**14**

^1^H NMR in CDCl_3_ (400 MHz)

**15**

^13^C NMR in CDCl_3_ (100 MHz)

**15**

^1^H NMR in CDCl_3_ (300 MHz)

**16**

^13^C NMR in CDCl_3_ (75 MHz)

**16**

^1^H NMR in CDCl_3_ (400 MHz)

**17**

^13^C NMR in CDCl_3_ (62.5 MHz)

**17**

^1^H NMR in CDCl_3_ (400 MHz)

**18**

^13^C NMR in CDCl_3_ (62.5 MHz)

**18**

# HPLC Chromatograms

**4o**

**4p**

**4q**

**4a**

**(*R*)-4b**

**(*S*)-4b**

**4c**

**4d**

**4e**

**4f**

**(*R*)-4g**

**(*S*)-4g**

**4i**

**4j**

**4k**

**4l**

**4m**

**4n**

**14**

**15**

1. J. Dong, Q. Xia, X. Lv, C. Yan, H. Song, Y. Liu, Q. Wang *Org. Lett.* **2018**, *20*, 5661-5665. [↑](#footnote-ref-1)
2. A.M. Deobald, A.G. Correa, D.G. Rivera, M.W. Paixao *Org. Biomol. Chem.* **2012**,*10*, 7681-7684. [↑](#footnote-ref-2)
3. E. Gòmez-Bengoa, J. Jiménez, I. Lapuerta, A. Mielgo, M. Oiarbide, I. Otazo, I. Velilla, S. Vera, C. Palomo *Chem. Sci.* **2012**, *3*, 2949-2957 [↑](#footnote-ref-3)
4. G. Sirvinskaite, C.S. Nardo, P. Muller, A.C. Gasser, B. Morandi, *Chem. Eur. J.* **2023**, *29*, e202301978. [↑](#footnote-ref-4)
5. C. Guyon, M.C. Duclos, M. Sutter, E. Métay, M. Lemaire, *Org. Biomol. Chem.* **2015**, *13*, 7067-7075. [↑](#footnote-ref-5)
6. L. Li, Z. Hou, P. Li, L. Wang, *J.* *Org. Chem.* **2022**, *87*, 8697-8708. [↑](#footnote-ref-6)
7. K.L. Ivanov, M.Ya. Melnikov, E.M. Budynina, *Synthesis* **2021**, *53*, 1285-1291. [↑](#footnote-ref-7)
8. T. Takuwa, T. Minowa, H. Fujisawa, T. Mukaiyama, *Chem.* *Pharm. Bull.* **2005**, *53*, 476-480. [↑](#footnote-ref-8)
9. E.C. Swift, T.M. Williams, C.R.J. Stephenson *Synlett* **2016**, *27*, 754-758. [↑](#footnote-ref-9)
10. X. Cao, Y. Zhang, *Green Chem.* **2016**, *18*, 2638-2641. [↑](#footnote-ref-10)
11. M.P. Plesniak, M.H. Garduno-Castro, P. Lenz, X. Just-Baringo, D.J. Procter, *Nat. Commun.* **2018**, *9*, 4802-4810. [↑](#footnote-ref-11)
12. S. Meninno, R. Villano, A. Lattanzi, *Eur. J. Org. Chem.* **2021**, *11*, 1758-1762. [↑](#footnote-ref-12)
13. A. Mattarei et al., *Eur. J. Med. Chem.* **2017**, *135*, 77-88. [↑](#footnote-ref-13)
14. B. Jung, M.S. Hong, S.H. Kang, *Angew. Chem. Int. Ed.* **2007**, *46*, 2616-2618. [↑](#footnote-ref-14)
15. L. Deiana, P. Dziedzic, G. Zhao, J. Vesely, I. Ibrahem, R. Rios, J. Sun, A. Còrdova *Chem. Eur. J.* **2011**, *17*, 7904-7917. [↑](#footnote-ref-15)
16. B.P. Bondziac, T. Urushima, H. Ishikawa, Y. Hayashi *Org. Lett.* **2010**, *23*, 5434-5437. [↑](#footnote-ref-16)
17. A.J. Das, H. Borgohain, B. Sarma, S.K. Das *Org.. Biomol. Chem.* **2020**, *18*, 441-449. [↑](#footnote-ref-17)
18. T. Kawasaki, Y. Matsumara, T. Tsutsumi, K. Suzuki, M. Ito, K. Soai *Science* **2009**, *324*, 492-495. [↑](#footnote-ref-18)
